# Metadynamics simulations reveal mechanisms of Na^+^ and Ca^2+^ transport in two open states of the channelrhodopsin chimera, C1C2

**DOI:** 10.1371/journal.pone.0309553

**Published:** 2024-09-06

**Authors:** Lindsey A. Prignano, Mark J. Stevens, Juan M. Vanegas, Susan B. Rempe, Robert E. Dempski

**Affiliations:** 1 Department of Chemistry & Biochemistry, Worcester Polytechnic Institute, Worcester, Massachusetts, United States of America; 2 Sandia National Laboratories, Albuquerque, New Mexico, United States of America; 3 Department of Biochemistry & Biophysics, Oregon State University, Corvallis, Oregon, United States of America; Shiv Nadar University, INDIA

## Abstract

Cation conducting channelrhodopsins (ChRs) are a popular tool used in optogenetics to control the activity of excitable cells and tissues using light. ChRs with altered ion selectivity are in high demand for use in different cell types and for other specialized applications. However, a detailed mechanism of ion permeation in ChRs is not fully resolved. Here, we use complementary experimental and computational methods to uncover the mechanisms of cation transport and valence selectivity through the channelrhodopsin chimera, C1C2, in the high- and low-conducting open states. Electrophysiology measurements identified a single-residue substitution within the central gate, N297D, that increased Ca^2+^ permeability vs. Na^+^ by nearly two-fold at peak current, but less so at stationary current. We then developed molecular models of dimeric wild-type C1C2 and N297D mutant channels in both open states and calculated the PMF profiles for Na^+^ and Ca^2+^ permeation through each protein using well-tempered/multiple-walker metadynamics. Results of these studies agree well with experimental measurements and demonstrate that the pore entrance on the extracellular side differs from original predictions and is actually located in a gap between helices I and II. Cation transport occurs via a relay mechanism where cations are passed between flexible carboxylate sidechains lining the full length of the pore by sidechain swinging, like a monkey swinging on vines. In the mutant channel, residue D297 enhances Ca^2+^ permeability by mediating the handoff between the central and cytosolic binding sites via direct coordination and sidechain swinging. We also found that altered cation binding affinities at both the extracellular entrance and central binding sites underly the distinct transport properties of the low-conducting open state. This work significantly advances our understanding of ion selectivity and permeation in cation channelrhodopsins and provides the insights needed for successful development of new ion-selective optogenetic tools.

## Introduction

In the free-swimming green alga *Chlamydomonas reinhardtii*, two light-gated ion channels, channelrhodopsin-1 (ChR1) and -2 (ChR2), serve as eyespot photosensors to facilitate phototaxis [1]. Activation of ChRs by illumination with blue light leads to the formation of an aqueous pore that conducts mono- and divalent cations across the membrane down their electrochemical gradients. In alga, the resulting rapid depolarization of the membrane initiates a signaling cascade that ultimately directs the mode of flagellar movement, enabling the organism to swim toward more favorable lighting conditions [2]. Due to their unique function, ChRs are widely used in the field of optogenetics as a less invasive means of controlling the activity of neurons and other excitable cells using light. By genetically encoding ChR proteins in cells, researchers can target a single cell or group of cells within a mixture selectively to study synaptic transmission [3], roles of specific cell types [4], vision restoration [5], and other physiological processes with unmatched temporal precision [6, 7]. The technique can be applied in cultured tissues and in freely moving animals [8–10].

Considering that ChRs have small unitary conductance (~40 fS for ChR2 [11] vs. ~20 pS for typical voltage-gated Na^+^ and K^+^ channels [12]) engineering new, custom-designed variants with optimized biophysical properties would broaden ChR’s potential utility in neuroscientific research and industrial applications such as bioproduct reactors and ion-selective materials [13–15]. However, since most cation ChRs are highly permeable to protons, increasing the single-channel conductance also increases the risk of intracellular acidification due to the high influx of protons with repeated activation and may compromise cell health [16]. The generation of new ChRs that select for only one or two ions of interest would be highly beneficial for use in cell type-specific applications.

Previously, fortuitous experiments have resulted in altered selectively. For example, ChR2-L132C-T159C (CatCh) shows enhanced Ca^2+^ selectivity [17, 18]. ChR2-E90Q shows 10 times higher selectivity toward Na^+^ than H^+^ as compared with ChR2-WT [19]. Interestingly, in ChR2, replacement of a glutamic acid at position 90 with lysine produced a chloride-conducting ChR that enabled inhibition of neural activity [20]. However, the molecular mechanism for cation transport in ChRs has not been resolved fully yet, and a better understanding of that mechanism can be expected to advance the development of new ChR-based tools.

**Fig 1:**
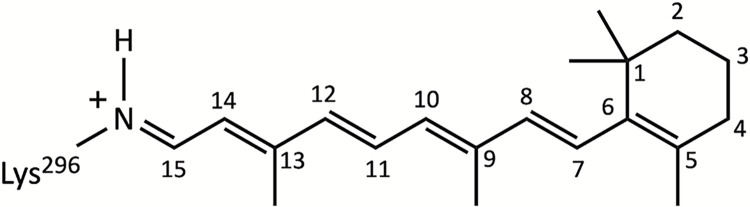
Structure of all-*trans* retinal. Chemical structure and carbon numbering scheme of the retinal chromophore in C1C2 in the all*-trans*, 15-*anti* configuration. The retinal is covalently bound to lysine 296 on helix VII via protonated Schiff base linkage.

A synthetic channelrhodopsin chimera containing helices I-V of ChR1 and helices VI-VII of ChR2, called C1C2, in the fully dark-adapted closed state became the first channelrhodopsin protein to have its crystal structure solved in 2012 (PDBID: 3UG9) [21], and provided critical insights relevant to ion permeation and the gating mechanisms of ChRs. The structure is composed of two identical protomers arranged in a tightly associating dimer. Each protomer consists of a seven-transmembrane helical bundle with an all-*trans*, 15-*anti* retinal chromophore (Fig 1) covalently bound to a conserved lysine residue (K296) at the bundle’s center and forms its own independent ion-conducting pore.

**Fig 2:**
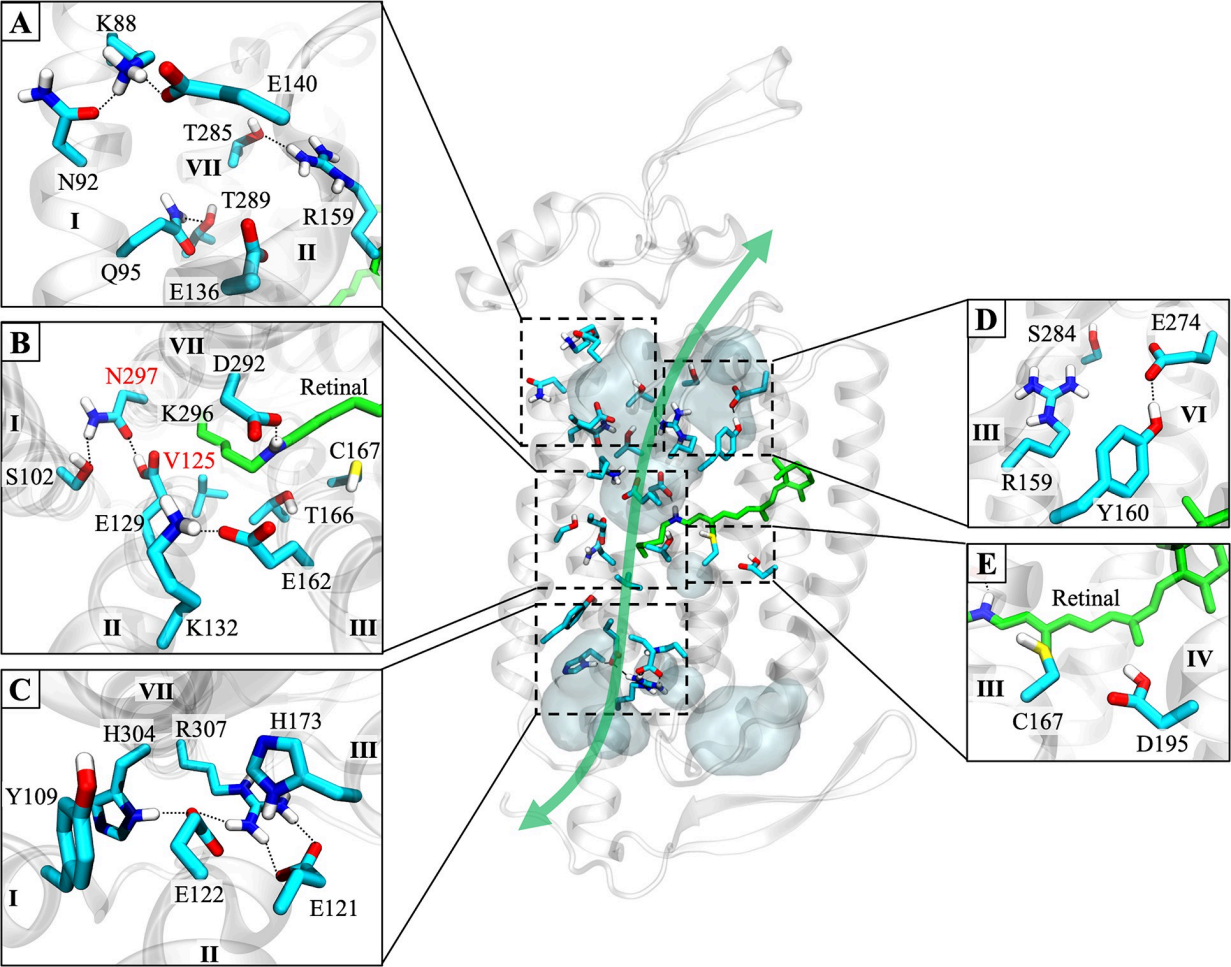
Key structural features involved in ion permeation and gating in C1C2. One protomer of our dimeric C1C2 wild-type model in the dark-adapted closed state is shown. The putative permeation pathway predicted by the crystal structure of closed state C1C2 (PDBID: 3UG9) is marked by a green double arrow. The pathway can be divided into three regions (viewed from the extracellular side): **(A)** The extracellular vestibule located between the tops of helices I, II, III, and VII forms a water-filled access channel that extends down to the central gate. **(B)** The central gate, including the protonated Schiff base of the retinal and its primary proton acceptor, D292. Hydrogen bonds between residues S102, E129, and N297 block the passage of water and ions from the extracellular side. Residues studied by mutational analysis in this work are N297 and V125 (labeled with red text). **(C)** The intracellular gate as seen from the central gate. Hydrogen bonds among these residues link helices II and VII and occlude the pore on the cytosolic side. Regions outside the permeation pathway that are involved in pore formation and cation conductance are (side views): **(D)** A highly conserved cluster of residues that borders the predicted channel entrance in cation ChRs. **(E)** The “DC-gate” formed by C167 and D195. Mutations at or near these two residues severely affect gating kinetics and ion selectivity. In all parts of the figure, the protein backbone is shown in transparent gray cartoon. Individual residues are shown as sticks in cyan, and retinal is green. Water inside the protein is displayed as transparent light blue-gray surfaces. Black dotted lines connecting sidechains represent hydrogen bonds. The other protomer, membrane lipids, bulk solvent, and nonpolar protons are hidden for clarity.

Based on the distribution of co-crystallized water molecules, permeating cations were predicted to traverse the channel via the path indicated by the green double arrow in Fig 2. The putative pathway closely follows a series of seven carboxylate residues lining the length of the pore wall along helix II that create a highly electronegative surface to attract cations. The bulk of this route is strongly supported by multiple pieces of evidence from the literature that show helices I-III and VII become hydrated just prior to the onset of light-induced currents [22, 23], and that point mutations made at polar or charged residues along this pathway significantly alter ion selectivity [20, 24, 25].

Yet, uncertainties regarding the pathway remain. In contrast to X-ray structures of tetrameric ion channels like KcsA [26] and NaK [27], the C1C2 crystal structure did not capture any permeating cations bound inside the channel nor bound to any site on the protein’s surface that would indicate a possible pore entrance or exit. Initially, a small water-filled tunnel between two extracellular loops (ECL1 and ECL3) that runs adjacent to a highly conserved cluster of four residues (Fig 2D) was identified as the most likely channel entrance [21]. However, results from MD simulations show that the water distribution within the channel changes substantially in the open state [28, 29] and the primary entrance actually may be through a cleft between the extracellular ends of helices I and II (Fig 2A) [30], as is the case for anion ChRs [31]. On the intracellular side, residues of the intracellular gate (ICG; Fig 2C) occlude the pore and mark the boundary for cytosolic water and ions in the crystal structure and in simulations [21, 28, 29, 32]. The channel’s exit most likely resides within the ICG, but the ICG spans a wide area where multiple paths may be possible in the open state. Thus, the exact locations of the channel’s entrance and exit for cations are not easily identifiable from the closed-state structure alone.

Curiously, several residues in other areas of the protein were shown to have a dramatic impact on ion selectivity even though they are located outside the proposed route and are not expected to interact directly with transiting cations. For example, the L132C mutation in ChR2 (L171 in C1C2) forms the calcium-permeable variant CatCh mentioned earlier [17], but L132 is located on the side of helix III facing away from the putative pore exactly one helix-turn below C128 of the DC gate (C167 in C1C2, Fig 2E). The mechanism by which the L132C mutation improves Ca^2+^ transport remains elusive, but some molecular dynamics (MD) studies speculate that cations may follow alternate routes through the channel that would partly explain the observed phenomena [29, 30, 33].

**Fig 3:**
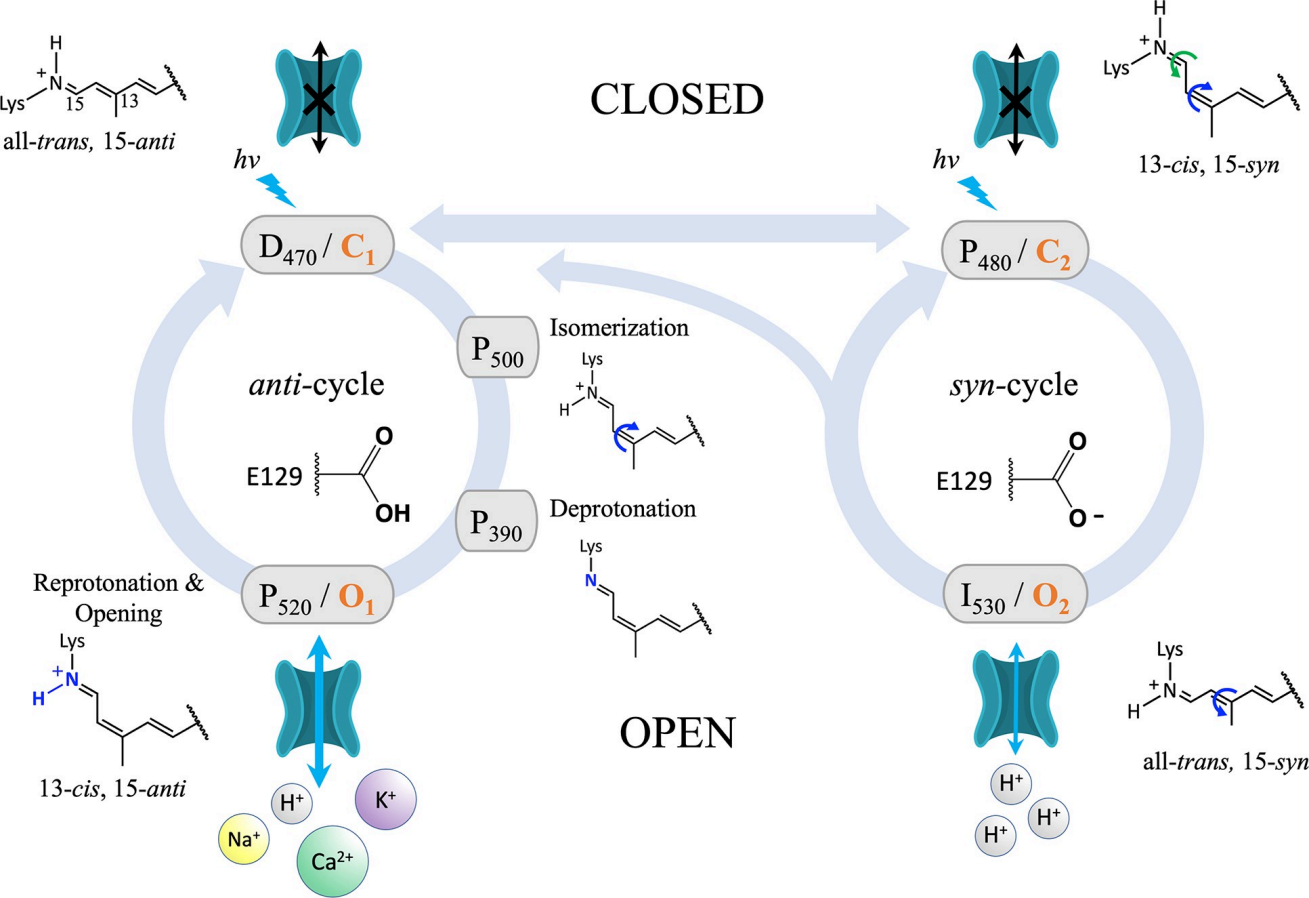
Photocycle of C1C2. The branched double photocycle model applied in this work, proposed by Kuhne et al. and features two parallel reaction cycles. The conformation of the retinal Schiff base (RSB) in each state is shown. Blue lightning bolts indicate light-activated transitions. Subscripts are the wavelength of maximum absorption characteristic of each photointermediate state. Orange text indicates the associated kinetic state from the four-state model used in previous works. For a population of channels on a cell membrane in the dark, all channels start in the dark-adapted first closed state D_470_/C_1_ with residues E129 and D195 protonated, retinal in the all-*trans*, 15-*anti* conformation and a protonated Schiff base. This state is also referred to as the ground state. Under single-turnover conditions, where the channels are activated with a very brief laser flash, the first reaction pathway is followed (*anti*-cycle). Residue E129 remains protonated throughout the *anti*-cycle. Photoisomerization of retinal from all-*trans*, 15-*anti* → 13-*cis*, 15-*anti* forms the first photointermediate state, P_500_. Next, proton transfer from the RSBH^+^ to the primary proton acceptor D292 forms the second photointermediate, the “pre-open” P_390_ state. Reprotonation of the RSB leads to the high-conducting first open state, P_520_/O_1_, that generates a transient peak current before decaying exponentially back to the baseline. Under continuous illumination, the photocycle branches from the ground state such that about half of the channels follow the *anti*-cycle reaction pathway while the other half follow a second reaction pathway, called the *syn*-cycle. In the *syn*-cycle, a double photoisomerization from all-*trans*, 15-*anti* → 13-*cis*, 15-*syn* retinal and deprotonation of E129 leads to formation of the light-adapted/desensitized second closed state P_480_/C_2_. Residue E129 stays deprotonated throughout the *syn*-cycle. Photoactivation of the P_480_/C_2_ state with 480 nm blue light triggers the third and final isomerization reaction from 13-*cis*, 15-*syn* → all-*trans*, 15-*syn* retinal that leads to the low-conducting or “inactivated” second open state, I_530_/O_2_. The two open states reach an equilibrium that generates a steady-state current until the light is turned off, at which point the current decays exponentially back to the baseline.

Furthermore, functional studies show that, like ChR2, photoexcitation of the all-*trans* retinal in C1C2 with 470 nm blue light initiates two parallel reaction cycles that each lead to channel opening, shown in Fig 3. Both photocycles generate a spectroscopically distinct open state with different photocurrent characteristics: the high-conducting dark-adapted P_520_/O_1_ open state of the *anti*-cycle, and the low-conducting light-adapted I_530_/O_2_ open state of the *syn*-cycle [34–36]. Both states conduct cations but with different relative selectivities that point to non-negligible differences in pore conformation [18, 24]. However, the specific structural determinants underlying the selectivity differences between these two open states have not been explored fully in the literature. This omission is partly because no high-resolution crystal structure of a fully open ChR in either state has been solved yet.

A recent study using time-resolved serial femtosecond X-ray crystallography (TR-SFX) was able to capture multiple structures of C1C2 during the early stages of channel opening [37]. While the formation of the fully open P_520_/O_1_ state was hindered in the crystal [37], these structures provide an abundance of information to help guide the development of atomistic models of C1C2 open states that accurately reflect their true forms. Since attaining an accurate open state model with a fully hydrated open pore is vital to the success of ion permeation studies with MD, this makes C1C2 an ideal choice for the study of cation transport mechanisms in ChRs.

In the present study, we elucidate the molecular mechanism of ion selectivity and transport through C1C2 by utilizing a combination of experimental and theoretical approaches. First, the impact of single-residue mutations on the channel’s preference for H^+^, K^+^, and Ca^2+^ transport relative to Na^+^ was assessed experimentally by measuring reversal potentials of the wild-type C1C2 protein and three previously generated central gate mutants V125L, N297D, and N297V [28] expressed in *Xenopus laevis* oocytes. Previous studies have demonstrated that the global electrostatic environment within the pore is a major determinant of cation vs. anion selectivity in channelrhodopsins [20, 25, 38, 39]. Since residue N297 is highly conserved among ChRs and is located along the putative permeation pathway in the central region of the channel, we hypothesized that introducing a negative charge at this position will increase the local electronegative surface potential and hydrophilicity in the pore and lead to enhanced relative Ca^2+^ permeability.

Our results show that this hypothesis was correct. All three mutants exhibited altered cation selectivity, but the most significant change was seen for the N297D mutant, which showed a two-fold increase in Ca^2+^ permeability relative to Na^+^ against the wild-type channel. Therefore, to elucidate the role of this highly conserved central gate residue in cation transport, and to uncover the chemical and mechanistic basis for valence selectivity in C1C2, the N297D mutant was chosen for further investigation in molecular dynamics studies alongside the wild-type channel for comparison.

Next, since obtaining meaningful results from ion permeation studies with MD requires accurate open state models with fully open and water-filled pores, we determined representative model structures of the dimeric C1C2 channel in the high-conducting P_520_/O_1_ and low-conducting I_530_/O_2_ open states for both the wild-type channel and N297D mutant. This was done by modeling all photointermediate states in series for both the *syn*- and *anti*-cycles to approximate the process of channel opening in a step-wise manner using classical MD. Complete channel opening and successful formation of continuous water-filled pores was achieved for both protomers of all four open state structures. A thorough analysis of the results show that our models are in excellent agreement with the experimentally observed changes to protein conformation and helix hydration associated with the onset of cation conductance in C1C2 [34, 35, 37].

Having attained open state models suitable for ion permeation studies, we next sought to identify the specific ion-protein interactions that drive (or limit) cation conduction in C1C2. We were also interested in determining the structural factors underlying the different transport properties between the two open states, as well as elucidating the mechanism by which the N297D mutation improves Ca^2+^ permeability. To address these questions, we performed multiple-walker/well-tempered metadynamics (MWWT-MetaD) simulations to calculate the one-dimensional potential of mean force (PMF) profiles for single-ion Na^+^ and Ca^2+^ permeation along the lowest free energy path through the high-conducting P_520_/O_1_ and low-conducting I_530_/O_2_ open states of the wild-type C1C2 and N297D mutant channels.

Results of these studies demonstrate that the extracellular entrance to the pore for cations is located through a gap between helices I and II and not through the entrance originally predicted from the closed state crystal structure of C1C2. Cations traverse the pore in a partially dehydrated state using a relay mechanism whereby cations are passed between adjacent carboxylate residues on helices II and VII via sidechain swinging in a manner reminiscent of a monkey swinging on vines. We also found that a local distortion of helix II that occurs during channel opening in the *syn*-cycle and the presence of negatively charged E129 and D292 along the pathway in the I_530_/O_2_ state underly the differential selectivity and conductance properties between the high- and low-conducting open states.

Simulations in the N297D mutant channel show that the N→D mutation increases both Na^+^ and Ca^2+^ permeability by assisting the handoff of cations between the central and cytosolic binding sites via the direct coordination of cations by D297 and sidechain swinging. This reduces the maximum free energy barrier for both cations, but the effect is greater for Ca^2+^ due to its +2 charge and higher free energy of hydration versus Na^+^, leading to increased Ca^2+^ permeability relative to Na^+^ in the mutant channel. Results of these studies show that Na^+^ and Ca^2+^ permeability is governed by the electronegativity and charge distribution of a series of low-affinity asymmetrical cation binding sites with flexible sidechains lining the full length of the pore in C1C2. Overall, the trends in the relative transport properties for Na^+^ and Ca^2+^ permeation across all four open state models developed here match the trends in cation selectivity observed from experimental measurements of wild-type C1C2 and N297D mutant channel photocurrents.

## Materials and methods

### Ethics statement

Animal care and handling was performed in strict accordance with the standards established by the Guide for the Care and Use of Laboratory Animals of the National Institutes of Health. Oocytes used in electrophysiology experiments were harvested from healthy adult *Xenopus laevis* frogs via partial ovariectomy using the anesthetic tricaine to minimize animal suffering. Written approval of the surgical protocol was granted by WPI’s Institutional Animal Care and Use Committee (IACUC).

### Molecular biology and expression of constructs in *Xenopus laevis* oocytes and electrophysiology

A plasmid containing the gene for C1C2 was obtained from Addgene (Addgene plasmid # 35519) [21]. The cDNA of the C1C2 chimera (residues 1–356) and C-terminal eYFP was subcloned into the vector pTLN as previously described [40–42]. Point mutations were introduced into the C1C2 gene using PCR-based site-directed mutagenesis [24] to generate three single-substitution variants of the encoded protein: V125L, N297D, and N297V. Successful mutation was confirmed by sequencing the entire gene. Synthesis of mRNA for wild-type and mutant C1C2 was done using the AmpliCap™ SP6 High Yield Message Maker Kit according to the manufacturer’s instructions (CELLSCRIPT, Madison, Wisconsin).

Oocytes from healthy adult *Xenopus laevis* frogs were collected via partial ovariectomy. Healthy, defolliculated stage V—VI oocytes were selected for injection with mRNA of wild-type and mutant C1C2 for two-electrode voltage clamp experiments. Oocytes were injected with 50 nL of 1 ng/nL mRNA in diethyl pyrocarbonate (DEPC)-treated water, then incubated at 17°C in oocyte Ringer’s isotonic buffer with Ca^2+^ (ORI+) supplemented with 1.5 uM all-*trans* retinal and 1 mg/mL gentamycin for 48–72 hours in the dark to allow for adequate protein expression at the membrane. For negative controls, only DEPC-treated water was injected, and no photo-induced currents were observed.

For calcium selectivity studies, activation of the oocyte’s endogenous Ca^2+^-activated Cl^-^ channels was suppressed by buffering intracellular calcium levels with the cell-permeant selective calcium chelator 1,2-*bis*(o-aminophenoxy)ethane-N,N,N’,N’-tetraacetic acid tetrakis(acetoxymethyl ester) (BAPTA-AM) as previously described [43]. On the day of electrophysiology experiments to evaluate Ca^2+^ selectivity, 10–15 mRNA-injected oocytes were transferred to a 35 mm culture dish filled with 5 mL of oocyte Ringer’s isotonic buffer *without* Ca^2+^ (ORI-) supplemented with 1 mg/mL gentamycin. Then, 16.5 μL of 30 mM BAPTA-AM stock in DMSO was added to the oocyte medium (final concentration = 100 μM) and incubated at 17°C for 1–2 hours in the dark just prior to two-electrode voltage clamp (TEVC) experiments.

Current-voltage relationship for each construct was determined from photocurrent traces recorded using the voltage step protocol (from membrane potential, *V*_m_, of -100 mV to +40 mV in +20 mV steps with 1.5 s illumination period after each step and a 10 s dark interval between light pulses) in Na^+^ or K^+^ bath solution at pH 7.0 or pH 9.0 (115 mM *X*Cl, 2 mM BaCl_2_, 1 mM MgCl_2_, 10 mM HEPES (pH 7.0) or 10 mM Tris (pH 9.0), where *X* = Na^+^ or K^+^). For calcium selectivity studies, 115 mM *X*Cl was replaced by 76.7 mM CaCl_2_ and adjusted to pH 7.0 (76.7 mM CaCl_2_, 2 mM BaCl_2_, 1 mM MgCl_2_, 10 mM HEPES (pH 7.0)). The reversal potential, *E*_rev_, was determined from the plot of photocurrent vs. membrane potential by linear interpolation, as previously described [40–42].

Ca^2+^ permeability ratios were calculated using reversal potentials measured from BAPTA-AM-treated oocytes in Ca^2+^ and Na^+^ bath solution at pH 7.0 using Eq (1) [44, 45],

$$E_{rev,Ca}-E_{rev,Na}=\Delta E_{rev}=\frac{RT}{F}ln\left(\frac{4\frac{P_{Ca}}{P_{Na}}{\left[Ca^{2+}\right]}_{o}}{{\left[Na^{+}\right]}_{o}[1+exp(\frac{E_{rev,Ca}}{\frac{RT}{F}})]}\right)$$ (1)

where R is the universal gas constant, *T* is the room temperature in Kelvin, F is Faraday’s constant, [Ca^2+^]_o_ and [Na^+^]_o_ are the concentrations of Ca^2+^ and Na^+^ in the external bath solutions, *E*_rev,Ca_ *and E*_rev,Na_ are the reversal potentials measured in Ca^2+^ and Na^+^ bath solutions, respectively, and $\frac{P_{Ca}}{P_{Na}}$ is the ratio of permeabilities of Ca^2+^ to Na^+^. Permeability ratios for monovalent ion *X* with respect to Na^+^ were calculated using the Goldman-Hodgkin-Katz (GHK) equation [12],

$$E_{rev,X}-E_{rev,Na}=\Delta E_{rev}=\frac{RT}{zF}ln\left(\frac{P_{X}{\left[X\right]}_{o}}{P_{Na}{\left[{Na}^{+}\right]}_{o}}\right)$$ (2)

where R is the universal gas constant, *T* is the room temperature in Kelvin, F is Faraday’s constant, *z* is the ionic charge, [*X*]_o_ and [Na^+^]_o_ are the concentrations of ion *X* (where *X* is K^+^ or H^+^) and Na^+^ in the external bath solutions, *E*_rev,*X*_ *and E*_rev,Na_ are the reversal potentials measured in ion *X* or Na^+^ bath solutions, respectively.

### Molecular dynamics simulations

Note that the structural models presented in this work differ from our prior models of C1C2 in the following ways. Firstly, in the present work we include the full dimeric biological assembly of the channel in all simulations presented here to account for the potential importance of dimerization on the channel’s structural stability [46, 47]. Secondly, we updated our applied photocycle model from the four-state kinetic model described previously [28, 48] to the more complete branched double-photocycle model proposed by Kuhne et al. (Fig 3) [34]. The branched double-photocycle model incorporates new data from recently published NMR and time-resolved spectroscopic studies that clarify previously unresolved details regarding the retinal conformation and the protonation states of certain ionizable residues present in each photointermediate state [34, 49, 50].

#### System preparation

An all-atom model of homodimeric C1C2 containing residues 24–348 with bound all-*trans*, 15-*anti* retinal (see Fig 1) for both protomers (A and B) was built from the X-ray crystal structure of protomer A (PDBID: 3UG9) in the fully dark-adapted closed state D_470_/C_1_ obtained from the Orientations of Proteins in Membranes (OPM) database [21, 51]. The I-TASSER server was used to model the disordered N-terminal, C-terminal, and loop regions that are not visible in the crystal structure [52]. The initial protein model plus co-crystallized water molecules were imported into Visual Molecular Dynamics 1.9.4 (VMD) molecular visualization software [53], where the transformation operations specified in the original PDB file (PDBID: 3UG9) were performed to create protomer B and complete the dimeric biological assembly of C1C2.

CHARMM-GUI Input Generator v3.7 [54–57] was used to generate the initial simulation box and input files for standard MD simulations in the Nanoscale Molecular Dynamics (NAMD) [58] software package. Initial protonation states of ionizable residues for the wild-type C1C2 dimer modeled in the dark-adapted closed state (D_470_/C_1_) were set using the CHARMM-GUI workflow. Based on results from our previous work modeling the closed state C1C2 monomer, all ionizable residues were modeled in their standard protonation states at physiological pH except for residues E129 and D195 which were protonated [32]. The retinal chromophore was kept in the all-*trans*, 15-*anti* conformation as in the crystal structure with a protonated Schiff base. Retinal conformation and all protonation states were the same for both protomers.

All structures presented here contained three disulfide bridges, observed in the crystal structure, which connect C66, C73, and C75 of protomer A to C66’, C75’, and C73’ on protomer B, respectively. All histidine residues were modeled as neutral with the proton assigned to either the δ- or ε-nitrogen by visual inspection of its local environment. The N297D mutant was generated by replacing the asparagine residue at position 297 with aspartate in both protomers. The dimer was then placed in a dioleoylphosphatidylcholine (DOPC) lipid bilayer by the replacement method, and the system was solvated with a 55 Å thick TIP3P water box on each side of the membrane and 150 mM NaCl, consistent with our prior works [28, 32]. The final dimensions of the simulation box were 13.0 nm x 13.0 nm x 14.8 nm and contained ~240K atoms (S1 Fig).

#### Classical molecular dynamics

All classical MD simulations were performed in NAMD 2.14 software for system minimization/equilibration cycles or NAMD 3.0α9 for production runs [58]. Systems were equilibrated using the standard minimization and equilibration scheme generated by the CHARMM-GUI workflow. Simulations were performed in the constant particle, pressure, and temperature (NPT) ensemble at 303.15°C with periodic boundary conditions, TIP3P water, and CHARMM36m (C36m) all-atom additive force field [59], as done previously [28, 32]. All bonds involving hydrogen were made rigid using the SETTLE algorithm. An integration timestep of 1 fs was used during equilibration and a 2 fs timestep was set for production runs. Constant pressure was maintained at 1 atm with the Nosé-Hoover Langevin piston method with an oscillation period of 50 fs and decay time of 25 fs. Constant temperature control was achieved by implementing Langevin dynamics with a damping coefficient of 1/ps. Full electrostatic interactions were calculated at every step with the particle mesh Ewald algorithm and a maximum grid spacing of 1.0 Å. Long-range electrostatic and van der Waals forces were calculated at every step with a switching distance of 10 Å and smoothly truncated at 12 Å. Coordinates were saved every 100 ps and trajectories were produced from 100 ns of unrestrained production run. Structural analysis was done as previously described [28, 32], and details are given in the data analysis section of the Methods.

#### Retinal isomerization and modeling of all photocycle states

To approximate the process of channel opening in the *anti*- and *syn-*photocycles, each photocycle state was simulated in sequence according to the C1C2 double photocycle model illustrated in Fig 3 beginning with the dark-adapted D_470_/C_1_ closed state. The same procedure was followed for both the wild-type C1C2 and N297D mutant channels. A list of all simulated models and their configurations is provided in S3–S6 Tables. All models presented in this work were equilibrated using the default CHARMM-GUI energy minimization and equilibration scheme followed by 100 ns of unrestrained production run prior to the generation of the next state in the series.

Please see the Methods in S1 Text for modeling details for photocycle states D_470_/C_1_, P_500_, P_390_, and P_480_/C_2_, as well as procedures for the all-*trans*, 15-*anti* → 13-*cis*, 15-*anti* retinal isomerization and the 13-*cis*, 15-*syn* → 13-*trans*, 15-*syn* retinal isomerization.

*Anti-cycle high-conducting P*_*520*_*/O*_*1*_ *open state*. The fully equilibrated P_390_ system was used to build the high-conducting open state, P_520_/O_1_. The optimal configuration of the P_520_/O_1_ open state channel was determined heuristically using a combination of p*K*_a_ analysis of the P_390_ system and evidence from published experimental data as a guide. For a more detailed rationale of our choice of protonation states in the P_520_/O_1_ open state models along with a discussion of the relevant literature, please see S1 Text. Accordingly, all models of the P_520_/O_1_ state contained retinal in the 13-*cis*, 15-*anti* conformation with a protonated Schiff base and with residues E129 and D292 protonated to match the literature [34, 60–62], while the protonation states of D195, H173, and H304 were varied to determine the configuration that best promotes pore formation in the *anti*-cycle. A total of six models for the wild-type P_520_/O_1_ open state dimer were simulated, and their configurations are listed in S3 Table. All other ionizable residues were modeled in their standard states at physiological pH.

The optimal structure for the dimeric N297D mutant channel in the P_520_/O_1_ open state was determined based on results from simulations of the wild-type channel. Accordingly, the last frame of the P_390_ pre-open intermediate of N297D was used to build two different structures for the P_520_/O_1_ open state of N297D. Their configurations are listed in S4 Table. For each protein, the system that formed a stable, fully open channel with the highest number of pore waters in both protomers within 100 ns of unrestrained production run was chosen for use in subsequent ion permeation studies (wild-type model #6, S3 Table; N297D model #2, S4 Table).

*Syn-cycle low-conducting I*_*530*_*/O*_*2*_ *open state*. The fully equilibrated P_480_/C_2_ system was used to approximate the 13-*cis*, 15-*syn* → 13-*trans*, 15-*syn* photoisomerization of retinal that leads to the formation of the light-adapted low-conducting open state, I_530_/O_2_. This procedure is described in S1 Text. Following isomerization, the resulting system was used to build the I_530_/O_2_ model with protonated Schiff base, D195 protonated, and E129 deprotonated, based on experimental evidence [34, 50, 63]. Since this led to complete channel opening in both protomers during the 100-ns unrestrained production run for both the wild-type and N297D mutant channels, these models were chosen for use in subsequent ion permeation studies.

#### Determining the PMF with multiple-walker/well-tempered metadynamics

**Fig 4:**
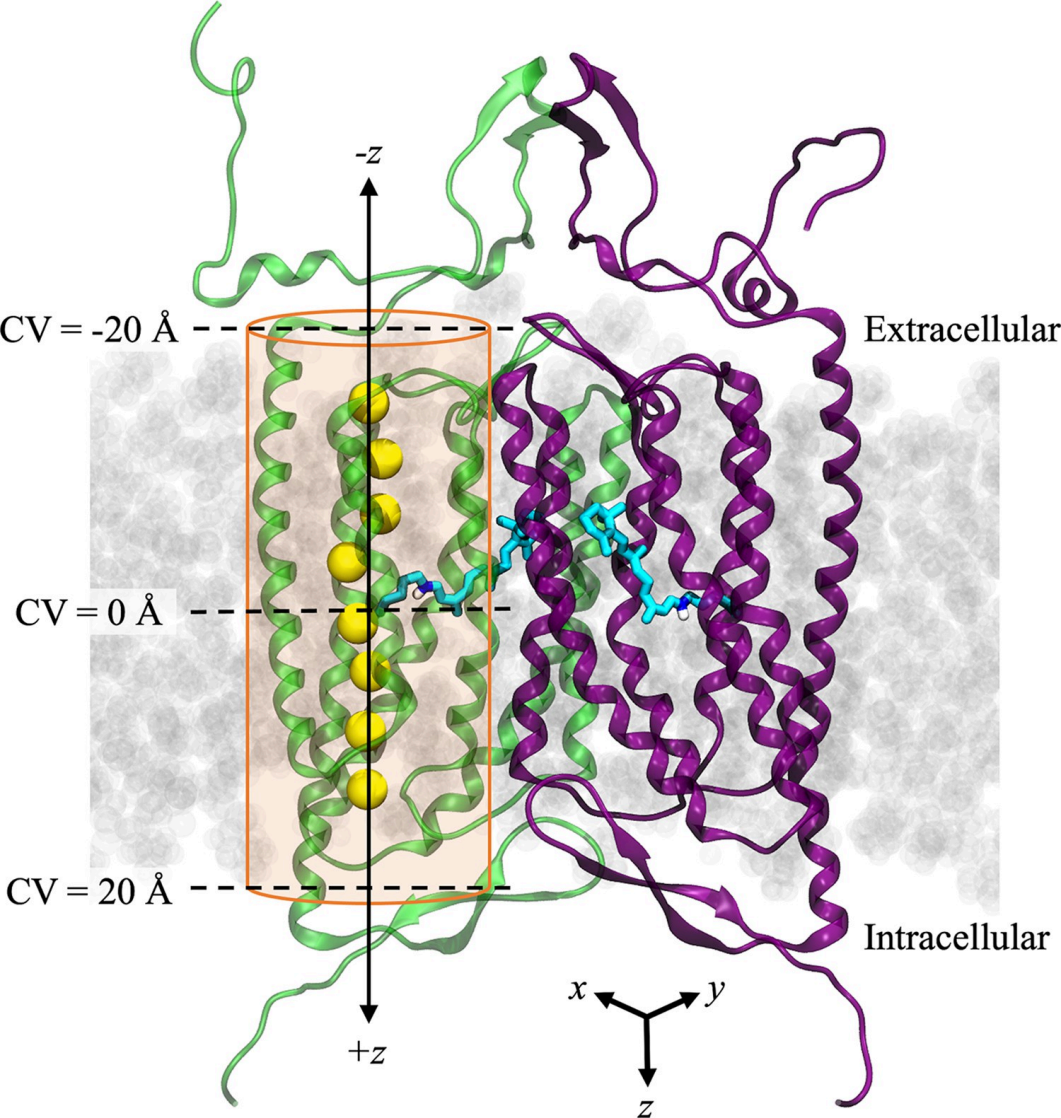
Setup of MWWT-MetaD with 8 “walkers”. Representative diagram of the simulated system showing initial positions of Na^+^ (yellow spheres) in each of the 8 replicas or “walkers” extracted from the SMD trajectory. Retinal is shown as sticks in cyan, protomer A is shown in green, protomer B is in purple, and lipids are light-gray space-filling molecules in the background. To prevent the ion from diffusing out of the channel, a harmonic wall potential was applied to limit the movement of the sodium ion to an area defined by a 14-Å radius cylinder (shown in orange) centered around the *z*-axis of the pore (solid black double arrow). The collective variable (CV) used for free energy calculations was defined as the *z-*axis component of the ion’s distance away from the center of mass of the helical core of protomer A. Thus, a CV value of zero was located about halfway through the membrane near the retinal (middle black dashed line), negative CV values were toward the extracellular side, and positive CV values were toward the intracellular side. Foreground lipids, water, and other ions are hidden for clarity.

MWWT-MetaD simulations [64, 65] were performed in NAMD 2.14 using the built-in metadynamics colvars module. The trajectory of ion translocation through the pore obtained from steered molecular dynamics (SMD) simulations (please see S1 Text for details) was used to generate the starting positions for eight equally spaced replicas or “walkers” (Fig 4, yellow spheres) for each protein system. Energy minimization was performed to minimize any potential carry-over of undesired perturbations to the system from the SMD simulation. This procedure was followed by 10 ns of equilibration for each walker while the ion’s motion was restrained by an umbrella potential of 5.0 kcal/mol·Å^2^. A one-dimensional collective variable (CV) was chosen to describe the translocation of the ion across the membrane. This CV was defined as the distance along the *z*-axis between the ion and the center of mass of the α-carbon atoms of the residues that form the helical core of monomer B. The reaction coordinate spanned from -20.0 Å to 20.0 Å along the channel axis in the *z*-dimension (Fig 4) and was discretized into bins 0.1 Å wide.

To prevent the ion from diffusing too far away from the protein and sampling irrelevant conformations, harmonic wall potentials were implemented in the *z*-direction and in the *xy*-plane such that the bias was only applied outside the channel beyond a certain threshold indicated by the orange cylinder in Fig 4. In the *z*-direction, a half-harmonic potential of 100 kcal/mol/Å acted on the ion if it traveled to within 1.0 Å of the CV boundaries. In the *xy*-plane, the bias was applied only beyond a threshold of 14.0 Å from the central axis of the permeation pathway. The potential was set to zero anywhere within the wall’s boundaries so that the ion could move freely within the space.

Metadynamics simulations for all eight walkers were run asynchronously with a 2-fs integration timestep on a shared filesystem. A time-dependent metadynamics biasing potential was added every 1 ps. This time interval was chosen based on the autocorrelation time of the CV measured during a short unbiased simulation. The added Gaussian hills had a height of 0.301 kcal/mol and width of 0.24 Å (σ = 0.12 Å). Well-tempered metadynamics was implemented with a biasing factor of 8.5, which corresponds to a bias temperature of 2273.63 K. The bias factor is based on an initial estimate for the largest free-energy barrier in the channel of ~ 5 kcal/mol and calculated as described in Bussi and Branduardi, 2015 [66]. All other system parameters were kept consistent with standard MD production runs described in the previous sections.

Simulations were run for 100–140 ns for each walker, or until the free energy converged, for a total simulation time of up to 1.12 μs per system. The full length of the trajectories of all eight walkers in each system were used for data analysis. Lastly, to facilitate the clear interpretation and presentation of the results, a baseline adjustment of the calculated PMF profiles was made such that the relative free energy of the ion in the bulk solution was set to 0 kcal/mol. The CV value where the ion samples the bulk solution was determined via post analysis of the ion-water radial distribution function from MWWT-MetaD trajectories, as described in the following section.

#### Data analysis

All analyses of trajectories from MD simulations and RMSD calculations were performed in VMD 1.9.4 with built-in or freely available tools, including VMD Extensions [67], p*K*_a_ Analysis scripts for analyzing interactions across trajectories [68], and our own custom shell or MathWorks MATLAB R2023b scripts. Figures were made using either MATLAB or GraphPad Prism version 10.1.1 for Mac software. Molecular representation graphics were rendered in VMD 1.9.4.

*Structural analysis of equilibrated photocycle state models*. Equilibration of each photocycle state model was assessed by calculating the RMSD of the helix backbone atoms against the unequilibrated structure over the course of a 100-ns production run, and the last 20 ns after the system had converged were used for structural analysis (200 frames) prior to the initiation of ion permeation studies. The structural features analyzed during the final 20-ns window were limited to either those that were no longer changing significantly (i.e. distances between helices) or to small local fluctuations that could be sampled adequately within this timeframe (i.e. hydrogen bonding patterns among interior sidechains) as done previously [28, 32]. Therefore, the 20-ns window provides sufficient sampling time for these purposes.

The following characteristics were measured for each protomer separately and are reported as the average ± SEM in S3–S6 Tables. Hydrogen bonds between sidechains were defined based on an average heavy atom separation of < 3.5 Å and average bond angle of 145-180° [69]. Strength of hydrogen bonding interactions and p*K*_a_ values of ionizable residues were calculated using the PROPKA 3.4.0 PyPI software package [70, 71]. The average number of water molecules in the pore (#H_2_O) was determined in VMD. The HII-HVII interhelix separation (*r*(HII-HVII) [Å]) was defined as the distance between helices II and VII at the pore exit on the intracellular side measured between the backbone C_α_ atoms of G300 and E122.

*Analysis of MWWT-MetaD simulations*. The full length of the trajectories from MWWT-MetaD simulations were used in the following analyses. Determination of pore size during ion translocation for each system was done in the software program HOLE [72] using the run-average coordinates of each walker taken from MWWT-MetaD simulations with ions and water removed. HOLE results from all eight walkers in the system were then averaged together to get the ensemble average of the pore dimensions as a function of the CV ± SEM, where *N* = 8. The hardcore.rad file supplied by HOLE was selected as the set of van der Waals radii used in the calculations [73]. Full-length trajectories from MWWT-MetaD simulations were imported into VMD to analyze the hydration shell occupancy of permeating cations as a function of the CV using the built-in Colvars Dashboard module and the coordNum colvar. The number of coordinating water molecules was defined as the number of water oxygen atoms within 3.0 Å of the ion for Na^+^, and within 4.0 Å for Ca^2+^ to include calcium’s second hydration shell. Direct coordination of the ion by the protein was determined in a similar manner with a cutoff distance of 3.0 Å for both cations.

## Results and discussion

### Electrophysiology: Central gate residue N297 is an important determinant of calcium selectivity

To evaluate the channel’s preference for one ion over another, photocurrents of the wild-type C1C2 channel and three previously studied central gate mutants [28]- V125L, N297D, and N297V -were recorded at a series of membrane potentials (*V*_m_) with the oocyte immersed in bath solutions of varying ionic composition (see Materials and Methods for recipes). Reversal potentials, *E*_rev_ (S1 Table), were calculated from the current-voltage relations (I-V curves) extracted from recorded photocurrent traces at peak and steady-state current. Shifts in reversal potentials measured for the mutant variants vs. the wild-type protein indicate mutation-induced changes to the ion selectivity of the channel.

**Fig 5:**
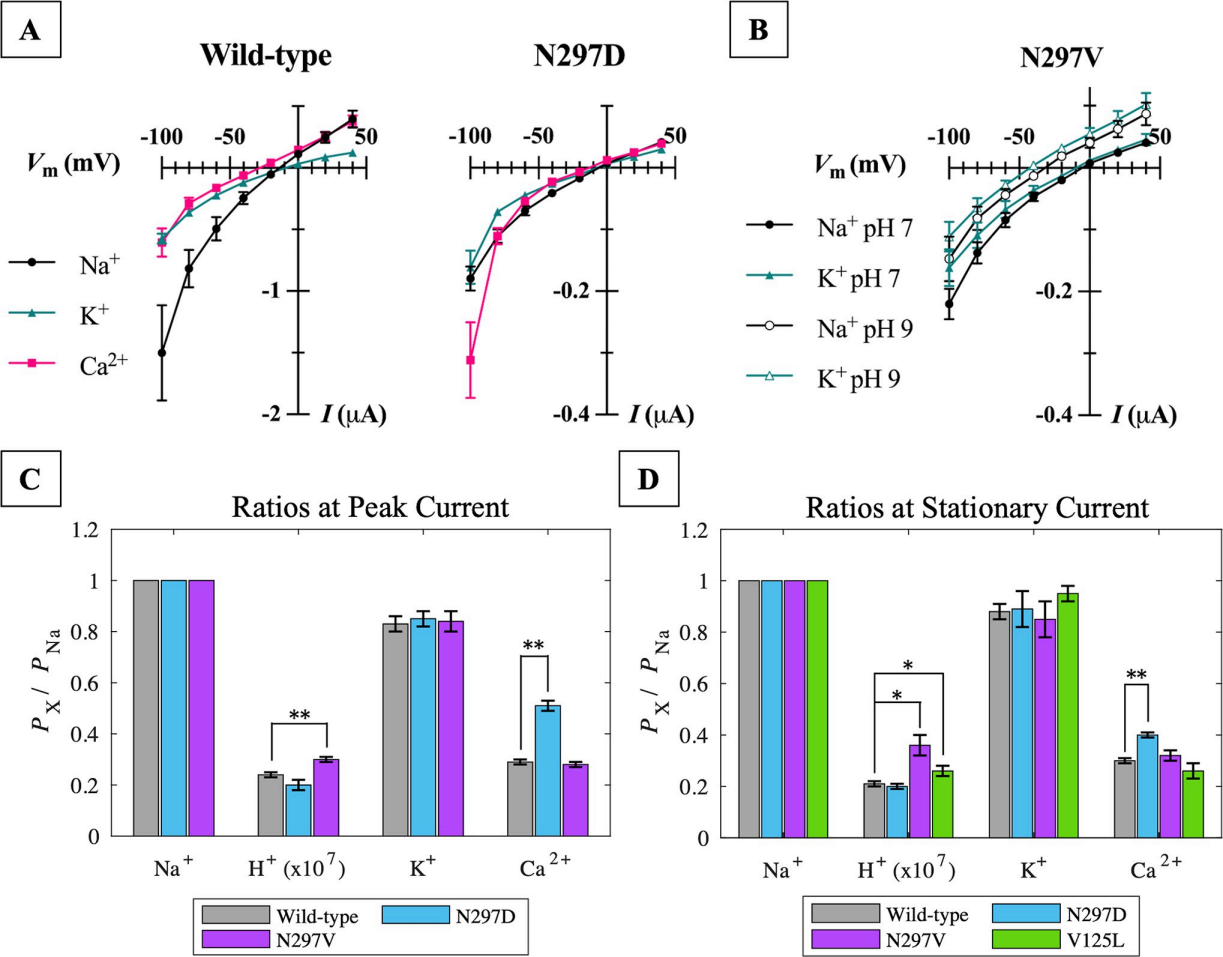
Characterization of ion selectivity in C1C2 wild-type and mutant channels. **(A)** Current-voltage relationships extracted from photocurrent traces of wild-type and N297D expressed in *X*. *laevis* oocytes immersed in the indicated bath solution at pH 7. Plotted values are the average of 6–9 cells ± SEM measured at peak current. **(B)** Current-voltage relationships extracted from photocurrent traces of N297V expressed in *X*. *laevis* oocytes immersed in the indicated Na^+^ or K^+^ bath solution at pH 7 or 9. Plotted values are an average of 5–6 cells ± SEM measured at stationary current. The reversal potential (*E*_rev_) is the membrane potential (*V*_m_) at the x-intercept of each curve in A and B. Reversal potential values measured in all bath solutions for all variants are listed in S1 Table. **(C)** Permeability ratios calculated from the difference in measured reversal potentials at peak current and **(D)** at stationary current. Ratios for V125L were only calculated for stationary current due the lack of peak current recovery after the initial light pulse. Proton permeability ratios *P*_H_/*P*_Na_ are scaled by a factor of 10^7^ for display purposes. Values are an average of 3–18 cells ± SEM. Statistical significance is denoted by * for *p* < 0.05 and ** for *p* < 0.01. Values shown in C and D are also available in numerical format in S2 Table.

The most significant *E*_rev_ shifts were seen for N297D and N297V. Only small changes were seen for the V125L mutation. Fig 5 shows the current-voltage relationships at peak current for the wild-type channel and N297D mutant (Fig 5A), and at stationary current for the N297V mutant (Fig 5B). The non-linear shape of the I-V curves and inward rectification of the ion flow are characteristic of nonselective pores facilitating passive ion transport down the concentration gradient. The steepness of the curve also suggests the presence of a voltage-sensitive gate/barrier within the pore [12].

Reversal potentials for V125L were calculated only for the steady-state current due to the absence of peak current after the first light pulse, as shown previously [28]. Valine 125 is situated just opposite the central gate in a narrow region of the pore. Conservative mutation of this residue to a leucine resulted in only modest negative shifts in reversal potential measured in Na^+^ solution at pH 7 and 9, and K^+^ solution at pH 7 (S1 Table). Calculating the permeabilities of each cation relative to Na^+^ reveals a slight increase in H^+^ selectivity compared to the wild-type channel (permeability ratio at stationary current *P*_H_*/P*_Na_: WT = 0.21 ± 0.01 x10^7^; V125L = 0.26 ± 0.02 x10^7^) while the relative selectivities of K^+^ and Ca^2+^ were unaffected.

At stationary current, substitution of polar asparagine 297 in the central gate with a neutral valine residue produced large negative shifts in reversal potentials measured in Na^+^ and K^+^ at pH 9. The same effect was observed at peak current, but to a lesser extent. In contrast, a small positive shift was seen for reversal potentials measured in Ca^2+^. Collectively, these shifts indicate a substantial increase in the channel’s preference for protons over Na^+^ compared to the wild-type channel (permeability ratio at stationary current *P*_H_*/P*_Na_: WT = 0.21 ± 0.01 x10^7^; N297V = 0.36 ± 0.04 x10^7^; Fig 5D). However, the negative shifts in *E*_rev_ were only observed at pH 9 and not at pH 7 (Fig 5B), where the contribution of H^+^ to the inward current is much higher. Therefore, the increase in *P*_H_*/P*_Na_ can likely be attributed to a mutation-induced loss of Na^+^ and K^+^ permeability in N297V rather than an increase in proton permeability.

The most notable change in permeability occurred when Asn 297 was exchanged for a negatively charged aspartate residue. The measured reversal potential increased drastically for Ca^2+^ at both peak and stationary currents, which translates to significantly enhanced calcium selectivity in N297D over wild-type by nearly two-fold (permeability ratio at peak current *P*_Ca_*/P*_Na_: WT = 0.29 ± 0.01; N297D = 0.51 ± 0.02; Fig 5C). Since the N297D mutation resulted in the most significant change in permeability observed here, we selected the N297D mutant for further investigation in MD simulations.

### Results from structural analysis of photocycle state models

For brevity, only results for the open state models P_520_/O_1_ and I_530_/O_2_ are presented in the main text. Modeling results for all preceding states of the *anti-* and *syn-*cycles (D_470_/C_1_, P_500_, P_390_, and P_480_/C_2_) can be found in S2 Text. Lists of all simulated models and their configurations are provided in S3–S6 Tables. For each system, the RMSD of the protein’s transmembrane helices stabilized within the first 60 ns of a 100-ns unrestrained production run (S2 Fig), indicating the system had converged.

#### *Anti-*cycle high-conducting open state P_520_/O_1_

In our initial attempt to model the dimeric wild-type C1C2 channel in the P_520_/O_1_ open state (see model #1 in S3 Table), deprotonation of D195 and concomitant reprotonation of the RSB had little to no effect on protein structure or internal water distribution compared to the P_390_ state, and a continuous water-filled pore did not form in either protomer. Thus, proton transfer from D195 to the RSB was not sufficient to open the channel under our simulation conditions.

Results for the N297D mutant with the same configuration (S4 Table, model #1) also did not produce a fully open pore, although N297D had a slightly higher influx of pore waters in the extracellular vestibule of one protomer compared to the wild-type. Interestingly, water molecules also entered the previously dry region of the DC-gate to bridge the sidechains of C167 and negatively charged D195. The additional water in this space caused the retinal polyene chain to shift toward helix VI due to hydrophobic effects, and the protonated Schiff base formed a new H-bond with the backbone carbonyl of nearby S295. Strengthening of the previously disrupted hydrogen bonds of the intracellular gate also occurred, and ultimately this gate completely closed toward the second half of the trajectory. Since the formation of a fully open and hydrated pore is critical to the success of ion permeation studies in the P_520_/O_1_ open state, this prompted us to investigate other possible configurations for our protein models in this state.

To help guide our selection of protonation states for molecular modeling of the P_520_/O_1_ state, we revisited the P_390_ state trajectories of each protein and analyzed the average p*K*_a_ values of titratable residues after the protein backbone had reached equilibrium, with a particular focus on ICG histidines and D195 whose protonation states are still unresolved in the literature (see S1 Text for discussion). Results for the N297D mutant channel in the P_390_ state revealed a negative shift for H173 (p*K*_a_ ± SEM: 3.10 ± 0.05 in protomer A; 3.55 ± 0.04 in protomer B) from the model p*K*_a_ reference value of 6.50 for histidine used by PROPKA. In contrast, a positive shift was seen for H304 (p*K*_a_ ± SEM: 6.90 ± 0.01 in protomer A; 7.36 ± 0.08 in protomer B) and reached a maximum p*K*_a_ of 9.28. Results were similar for the wild-type protein. Therefore, H173 likely remains neutral, while H304 could become positively charged or facilitate proton transfer during the P_390_ → P_520_/O_1_ transition.

For the wild-type C1C2 channel in the P_390_ state, the p*K*_a_ of D195 hovered around 7 and hardly varied (p*K*_a_ ± SEM: 7.15 ± 0.01 in protomer A; 7.02 ± 0.01 in protomer B). A similar result was seen for the P_390_ state of the N297D mutant channel. This is a substantial positive shift from the model p*K*_a_ reference value of 3.80 for aspartate. Furthermore, the calculated p*K*_a_ of D195 was largely unaffected by the protonation state of the retinal Schiff base since these values did not change significantly when retinal was protonated in model #1 of the P_520_/O_1_ state for either protein, confirming previous results [28]. This indicates that D195 could be either neutral or negatively charged in the open state.

Thus, to determine the configuration that best promotes channel opening in the P_520_/O_1_ state of C1C2, six different structures of dimeric wild-type C1C2 in the P_520_/O_1_ state were simulated by varying the protonation states of D195, H173, and H304 until a continuous water-filled pore was achieved in both protomers. For each model, the average number of water molecules in the pore were calculated for both protomers individually and used as a metric to gauge the degree of channel opening. Their configurations and analysis results are given in S3 Table.

The model that achieved a fully open channel most representative of the high-conducting P_520_/O_1_ open state in both protomers was model #6, which contained retinal in the 13-*cis*, 15-*anti* conformation with a protonated Schiff base, residues E129, D195, and D292 modeled as protonated, and residues H173 and H304 modeled as neutral but with the titratable proton moved from the δ-N to the ϵ-N position. This configuration led to the highest influx of pore waters out of all six models with an average increase of 33–39 water molecules in each protomer compared to the P_390_ state, bringing the total to 75 ± 0.3 pore waters per protomer (S3 Table). A snapshot of the internal water distribution for one protomer is shown in S3 Fig. As shown, the invading water formed a continuous and fully hydrated pore. Therefore, model #6 was selected for further investigation in ion permeation studies.

**Fig 6:**
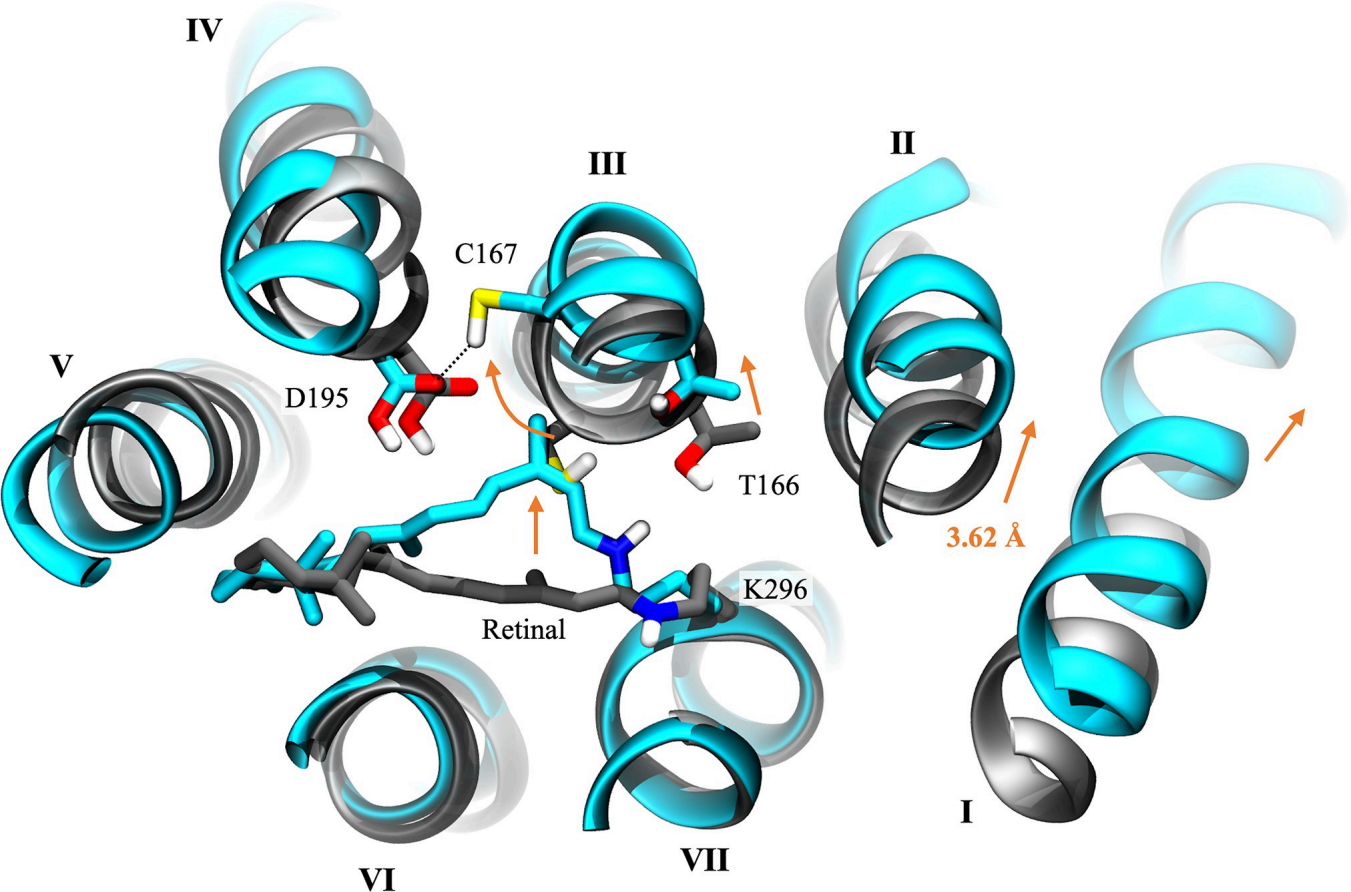
Retinal kink and conformational change upon channel opening. Structural alignment of one protomer of the wild-type C1C2 dimer comparing differences between the initial D_470_/C_1_ closed state (gray) and the high-conducting P_520_/O_1_ open state (cyan; S3 Table, model #6) in the vicinity of the retinal. View is from the extracellular side of the channel. Orange arrows indicate the direction of movement of residues and helices. The curved orange arrow traces the path taken by the sidechain of C167 upon channel opening, whose initial position in the D_470_/C_1_ closed state model is partially obscured by the retinal polyene chain of the P_520_/O_1_ open state model. Black dotted lines represent hydrogen bonds. The other protomer, nonpolar protons, solvent, ions, and lipids are hidden for clarity.

Recall that the central gate had already opened during the simulation of the preceding photointermediate, P_390_, but the intracellular gate remained partially closed. Complete channel opening in model #6 of the P_520_/O_1_ open state was triggered by conformational changes in two key regions of the channel: the retinal binding pocket and the intracellular gate. The most prominent changes were seen in the retinal binding pocket, where distortion of the retinal’s shape induced the rearrangement of nearby sidechains and moderate shifts in protein backbone, as illustrated in Fig 6. Reprotonation of the RSB induced the transient formation of an H-bond between the sidechain of T166 on helix III and the Schiff base proton, causing the retinal to twist and bend outward toward helix III. The kinked conformation of the retinal polyene chain induced a lateral movement of helix III toward the dimer interface that exaggerated the bend in the middle of the helix caused by proline 168. This pushed the sidechain of C167 further into the space between helices III and IV and strengthened its H-bond with D195.

Simultaneously at the intracellular gate, switching the titratable proton from the δ-N to the ϵ-N position in both H173 and H304 disrupted the interactions between their hydrogen bonding partners E121 and E122, respectively. This caused E122 and R307 to become more mobile and move apart. The sidechain of R307 then folded back nearly flat against the backbone of helix VII allowing enough space between helices II and VII for water to pass freely. In proteins, tautomerization of a solvent-accessible histidine (e.g. proton transfer from the δ-N to the ϵ-N on the imidazole ring) indicates that it may be transiently protonated [74]. Since tautomerization of histidines H173 and H304 rapidly promoted the complete opening of the intracellular gate during the P_390_ → P_520_/O_1_ state transition (see S3 Table), residues H173 and/or H304 may take part in an intramolecular proton transfer reaction during the photocycle that triggers opening of the intracellular gate in the P_520_/O_1_ state.

The reduced number and strength of interactions between helices II and VII also increased the mobility of their backbones. This enabled the lateral movement of retinal and helix III to translate to the intracellular end of helix II through an interhelical hydrogen bonding network formed by residues E129, K132, and E162, and resulted in a large outward shift of the helix II backbone as water filled the channel. The shift dilated the cytosolic entrance so that larger cations may pass by increasing the separation between helix II and VII on the intracellular side by an average cumulative distance of 3.62 ± 0.06 Å in protomer A and 1.74 ± 0.05 Å in protomer B vs. the D_470_/C_1_ closed state. This result is in excellent agreement with the helix-II-tilt model of channel opening, where low-resolution X-ray crystallography and EPR spin distance measurements in ChR2 showed a distinct outward movement of helix II by ~1.5–4.0 Å following light activation [46, 47, 75, 76].

Initial development of a retinal kink and outward shift of helix III were also observed in a recent TR-SFX study of the P_390_ pre-open intermediate of C1C2 and were identified as vital steps that lead to pore formation in the P_520_/O_1_ open state [37]. In that paper, QM/MM modeling of their X-ray structure to generate the P_520_/O_1_ open state led to changes in protein conformation that are nearly identical to those observed in our P_520_/O_1_ open state model obtained using classical MD methods.

Additionally, of the wild-type P_520_/O_1_ state models simulated here, channels in which the retinal maintained planarity (models #1 and 3) resulted in little to no water influx compared to those that adopted a twisted retinal (models #2, 4, 5, and 6) regardless of whether the inner gate opened successfully. This is likely due to an unfavorable electrostatic environment caused by the presence of a negative charge on D195 in models #1 and 3 that prevented the lateral movement of the retinal polyene chain toward helix III. Thus, under our simulation conditions, the sidechain of D195 must be neutral (protonated) in the P_520_/O_1_ state for the pore to expand to a size suitable for cation permeation.

Since model #6 was the only model that produced a fully open and hydrated pore in both protomers of the wild-type channel, the same configuration was applied to the N297D mutant channel to generate the P_520_/O_1_ open state (S4 Table, model #2). The retinal configuration and assigned protonation states of sidechains were the same for both protomers. Results of model #2 of the N297D mutant in the P_520_/O_1_ open state closely resembled model #6 of the wild-type channel. Hydrogen bonding patterns among functional residues in N297D were identical to that of the wild-type protein (S4 Fig).

The same conformational changes that were observed in model #6 of the wild-type channel in the region of the retinal and among pore-forming helices were also seen in model #2 the N297D mutant and similarly led to the formation of continuous water-filled pores in both protomers. Tautomerization of H173 and H304 triggered complete opening of the intracellular gate that allowed an additional ~10–37 water molecules to enter the channel versus the P_390_ state and brought the total number of pore waters to 68 ± 0.3 in protomer A and 84 ± 0.4 in protomer B (S4 Table, model #2).

Retinal kink-induced movement of helix III resulted in a cumulative outward shift of helix II by 1.27 ± 0.04 Å in protomer A and 3.59 ± 0.07 Å in protomer B vs. the D_470_/C_1_ closed state, giving an intracellular opening of about the same size as the wild-type channel. Since a completely open and water-filled pore was achieved here, this model was selected for use in subsequent ion permeation studies to investigate Na^+^ and Ca^2+^ transport through the N297D mutant channel in the high-conducting P_520_/O_1_ open state.

#### *Syn*-cycle low-conducting open state I_530_/O_2_

In both protomers of the wild-type C1C2 model structure in the I_530_/O_2_ open state, retinal isomerization to the 13-*trans*, 15*-syn* conformer reoriented the RSBH^+^ proton to point toward the intracellular side of the channel, which severed its hydrogen bond with D292 that fixed retinal’s position in the binding pocket. The retinal polyene chain then shifted laterally toward C167 on helix III and adopted a bent conformation resembling that of the P_520_/O_1_ open state in the *anti*-cycle (Fig 6). This caused C167 to rotate further around helix III toward helix IV and the dimer interface such that the thiol group on C167 donated a bifurcated H-bond to the two oxygens of the D195 sidechain.

Subsequent outward bending of helix III triggered an outward shift of the intracellular side of helix II by an additional ~1.5 Å from the P_480_/C_2_ closed state, giving a cumulative helix II shift of ~2.1–2.3 Å against the D_470_/C_1_ ground state (S5 Table). The average distance between helices II and VII on the intracellular side of the fully equilibrated protein measured 8.06 ± 0.03 Å in protomer A and 8.50 ± 0.04 Å in protomer B. Thus, in the wild-type channel, the intracellular pore opening in the light-adapted low-conducting I_530_/O_2_ open state was ~1 Å smaller than the opening in the dark-adapted high-conducting P_520_/O_1_ open state.

Development of the retinal kink also led to significant weakening/loss of key H-bonds and coulombic interactions among sidechains linking helices I, II, and VII. As shown in S5 Fig for the central gate, residue E129 frequently alternated between interacting with K132 and S102, and only E162 formed a stable salt bridge with K132. The combined effects of decreased interhelical sidechain interactions and the movement of pore-lining helices prompted the ICG to fully open. This allowed an additional ~28–35 water molecules to invade the channel from the intracellular side and form a continuous water-filled pore in both protomers (S3 Fig).

In the N297D mutant channel, equilibration of the I_530_/O_2_ open state model led to a similar rearrangement of sidechain interactions as the wild-type C1C2 channel. A comparison of the hydrogen bonding networks between the two proteins in this state revealed that the only significant difference was in the position of R159 (S5 Fig). Instead of interacting with T285 as in the wild-type channel, the sidechain of R159 in the N297D mutant formed a double H-bond with both E136 and E162 in protomer A, and with D292 in protomer B. All other sidechain interactions were the same as the wild-type C1C2 protein for both protomers.

Furthermore, the same changes in the conformation of the retinal polyene chain and helix backbone that led to channel opening in wild-type C1C2 were also seen in the N297D mutant channel. The consequent outward shift of the intracellular end of helix II dilated the cytosolic entrance to about the same size as the high-conducting P_520_/O_1_ open state of N297D and about ~1 Å larger than the low-conducting I_530_/O_2_ open state of the wild-type channel. The shift in the helix II backbone fully opened the ICG and allowed the largest influx of water molecules observed here for a total of 87 ± 0.3 pore waters in protomer A, and 95 ± 0.5 pore waters in protomer B.

Since the models of the I_530_/O_2_ open state for both the wild-type C1C2 and the N297D mutant channels gave fully open and continuous water-filled pores, these structures were selected for use in ion permeation studies as discussed in the following sections.

### Mechanism of Na^+^ and Ca^2+^ transport through the open C1C2 channel

A total of four dimeric C1C2 open state structures were selected for further investigation in computational ion permeation studies: 1) Wild-type C1C2 high-conducting P_520_/O_1_ open state model #6, 2) N297D high-conducting P_520_/O_1_ open state model #2, 3) Wild-type C1C2 low-conducting I_530_/O_2_ open state, and 4) N297D low-conducting I_530_/O_2_ open state (see S3–S6 Tables for model data). One-dimensional single-ion PMF profiles were calculated for Na^+^ and Ca^2+^ permeation through one protomer of each of the four structures using MWWT-MetaD, producing a total of eight independent PMFs. For all systems, a plot of the calculated RMSD between sequential PMF outputs versus simulation time (S7 Fig) trended toward zero as the simulation progressed until a steady value was reached, indicating good convergence of all PMFs.

#### Ion permeation pathway and channel entrance

**Fig 7:**
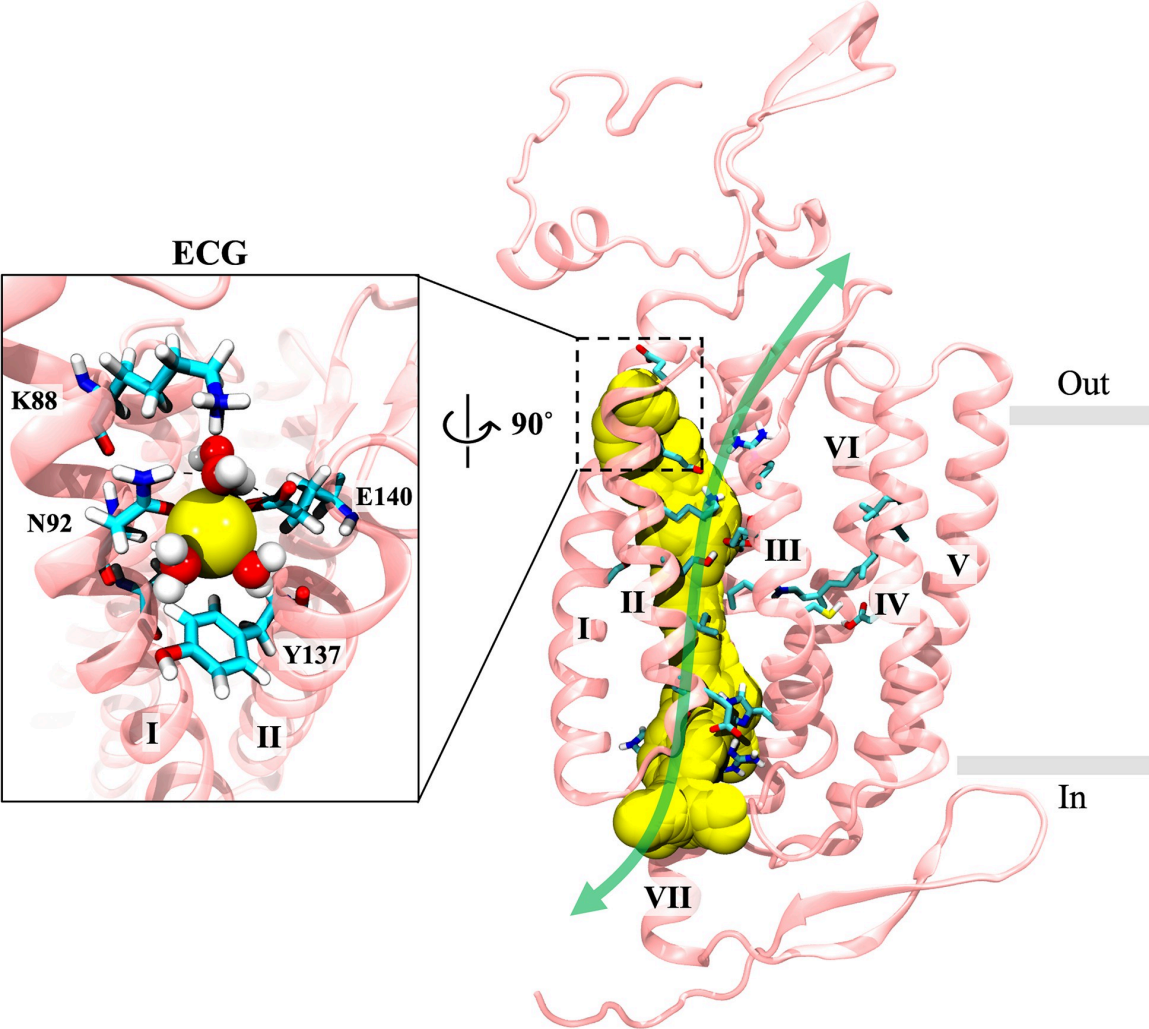
Predicted vs. actual permeation pathway in C1C2. Merged image of all frames of a 100-ns trajectory of one of eight walkers from metadynamics simulations of the wild-type P_520_/O_1_ open state model (*anti*-cycle) showing the actual permeation path taken by Na^+^ through the pore (yellow spheres) compared to the pathway predicted by the crystal structure (green double arrow). The window on the left shows a close-up snapshot of Na^+^ and coordinated waters as it passes through the pore entrance located between helices I and II on the extracellular side. This perspective is rotated 90° relative to the view on the right. The protein backbone is in pink with select individual residues represented as sticks in cyan. Na^+^ is shown in yellow, and coordinated waters are shown as red and white space-filling molecules. Gray bars mark the membrane boundaries. The other protomer, bulk solvent, and lipids are hidden for clarity. ECG, extracellular gate.

Results from MWWT-MetaD simulations for Na^+^ and Ca^2+^ permeation through C1C2 show that the lowest free energy path for cations through the pore differs somewhat from the pathway originally predicted from the closed-state crystal structure of C1C2 [21]. Fig 7 shows the actual path taken by Na^+^ (yellow spheres) through the channel during MWWT-MetaD simulations compared to the originally predicted pathway (green double arrow). Furthermore, Ca^2+^ was found to take the same route as Na^+^, and results were consistent across all eight replicas for both cations.

For the sake of brevity, we will focus only on the high-conducting P_520_/O_1_ open state of the wild-type C1C2 channel in our discussion of the pathway but note that the same route was found in all four systems studied. As seen in Fig 7, permeating cations passed through the intracellular and central gates while closely following the series of carboxylate residues lining transmembrane helix II, in agreement with earlier predictions. However, the pathway diverges considerably from the predicted one regarding the location of the channel entrance on the extracellular side.

Recall that a highly conserved cluster of residues at the extracellular ends of helices III, VI, and VII forms a water-filled conduit that connects the extracellular vestibule with the external milieu in the closed state (Fig 2D). This slightly electropositive opening was originally predicted to be the channel entrance for protons and larger cations [21]. In our simulations, although a steady column of water was maintained within the passage in the open state, neither Na^+^ nor Ca^2+^ was ever seen moving through that region of the channel. Instead, cations preferentially passed through a water-filled crevice between the tops of helices I and II that had formed during channel opening, giving a slightly bent route.

As described in the Methods, the metadynamics bias potential was applied to the ion’s movement along the *z*-dimension only, whereas its motion in the *xy*-plane within the channel was unbiased and unrestrained. Outside of the channel, a harmonic wall potential in the shape of a 14-Å radius cylinder was implemented to prevent the ion from migrating too far away from the protein. However, this boundary was rarely encountered and only at the very far limit of the CV in the cytosolic bulk solution. Therefore, we can conclude that this boundary had no influence on the ion’s chosen route through the pore, and that any lateral shift in the permeation pathway found here is due to the ion moving spontaneously toward sites that facilitate more favorable ion-water and/or ion-protein interactions.

In further support of our findings, multiple spontaneous permeation events of unbiased and unrestrained Na^+^ originating from the bulk solution were observed in one or both protomers during some simulations of Na^+^ and Ca^2+^ translocation. In most cases, bulk Na^+^ entered from the extracellular side through the water-filled opening between the tops of helices I and II shown in Fig 7. Occasionally, bulk Na^+^ would also enter from the cytosol through the intracellular gate formed by residues E121, E122, H173, H304, and R307. In all cases, the unbiased cations followed the same route through the pore as the biased cations. Bulk Cl^-^ did not enter the channel from any entrance. Also, no ions were observed entering or exiting the pore through the opening near the conserved cluster between extracellular loops I and III.

Therefore, we propose that the extracellular entrance to the channel for cations is actually located in the gap between the tops of helices I and II, as seen for some anion ChRs [31], and not through the water-filled tunnel formed by extracellular loops I and III as originally suggested by the crystal structure. This assignment is consistent with mutational analysis reported by Berndt et al. where substitution of ECG residues Q95, E136, or E140 with positively charged lysine or arginine nearly abolished the photocurrent while also shifting the reversal potential by up to -40 mV [25]. Furthermore, this region was previously identified as a potential cation binding site in early homology models of ChR2 in the dark-adapted closed state [30, 77], but the current study is the first to provide compelling evidence of its role as the channel entrance where unbiased cations were observed entering and transiting the pore spontaneously from the bulk solution.

The entrance is off-axis relative to the rest of the pathway and nearly level with the polar headgroups of the membrane lipids, placing it in a position ideal for attracting cations closely associated with the membrane surface. Several polar residues on helices I and II frame the opening. Residues K88, N92, Y137, and E140 are oriented to face the exterior of the protein as shown in Fig 7, and Q95 and E136 are positioned just inside the channel (obscured by Na^+^ and water in the figure). Collectively, these residues form an electronegative surface that provides a more suitable environment for attracting small positively charged ions compared to the slightly electropositive surface at the opening between extracellular loops I and III.

The remainder of the permeation pathway determined from MWWT-MetaD simulations agrees well with earlier predictions based on the closed-state crystal structure [21]. In the central region of the channel, water and cations passed through the space adjacent to the retinal Schiff base and directly between central gate residues N297 and E129. No ions were observed passing through the water-filled space between the bend in the retinal polyene chain and helix VI, which was speculated to be an alternate route for cations in another computational study of an early P_390_ pre-open state model of C1C2 [29]. However, considering the persistent flow of water through this area, we cannot rule out the possibility that it could serve as another route for H^+^-only transport via a water wire-type mechanism.

Lastly, on the intracellular side of the protein, Na^+^ kept close to E121 and E122 on helix II before exiting the pore into the cytosol by either swinging under the first intracellular loop or by traveling through the space between the ends of helices II and III via residues E121, H173, and N182. While Ca^2+^ also exited the pore via these same routes, Ca^2+^ showed a strong preference for the exit between helices II and III on the cytosolic side of the protein.

#### Transport mechanism in the high-conducting P_520_/O_1_ open state of wild-type C1C2

**Fig 8:**
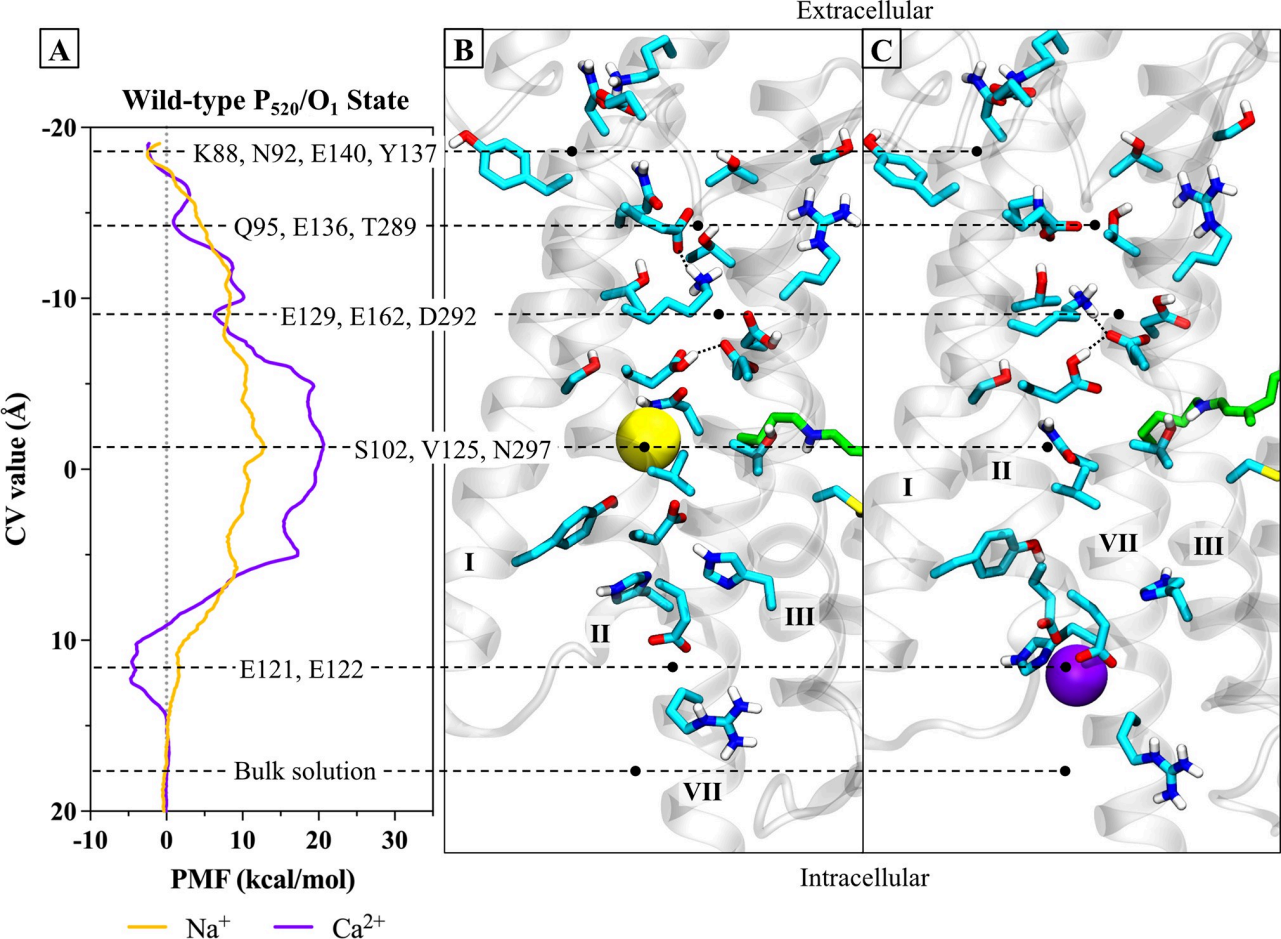
Correlation of PMFs to pore structure of the P_520_/O_1_ open state of the wild-type channel. **(A)** PMF profiles of Na^+^ (yellow line) and Ca^2+^ (purple line) permeation through one protomer of the wild-type C1C2 channel in the high-conducting P_520_/O_1_ open state calculated from metadynamics simulations. **(B)** Snapshot of the permeation pathway from metadynamics simulations as Na^+^ (yellow sphere) passes through the central gating region of the pore. **(C)** Snapshot of the permeation pathway from metadynamics simulations showing Ca^2+^ (purple sphere) in a binding site at the intracellular entrance to the pore. The snapshots in B and C are aligned to the reaction coordinate (CV value) of the PMF profile in A, where the horizontal black dashed lines correlate points on the PMF with important cation-sidechain interactions as the ion traverses the pore. Small, black dotted lines connecting sidechains indicate hydrogen bonds. The protein backbone is shown in transparent gray cartoon, sidechains of select residues are represented as sticks in cyan, and retinal is in green. The other protomer, solvent, lipids, and nonpolar protons are hidden for clarity.

The PMF profiles calculated for Na^+^ and Ca^2+^ permeation along the discovered route allow us to examine the significant cation-protein interactions in more detail. Fig 8 aligns the PMFs (panel A) for the high-conducting P_520_/O_1_ open state of the wild-type C1C2 channel to the reaction coordinate (panels B and C) to illustrate how key features of the free energy curves correspond to specific sites along the pathway. Since the C1C2 photocurrent is somewhat inwardly rectified, we will describe the transport mechanism in the ion’s preferred direction of travel from the extracellular side to the cytosolic side of the membrane.

On the extracellular side, the PMF for Na^+^ transport (yellow line) has a shallow free energy well with a depth of about -2.5 kcal/mol relative to the bulk solution that corresponds to the extracellular entrance at CV ≈ -18.5 Å and that is about ~ 8 Å off to the side of the pore’s central axis. Here, Na^+^ is guided through the gap between the tops of helices I and II via direct and water-mediated interactions with polar and charged sidechains K88, N92, E140, and Y137 that form a flexible ring around the mostly hydrated ion, as shown in the detailed view of the ECG in Fig 7. Locations where a local minimum in the calculated PMF correlates with direct coordination of the ion by one or more protein ligands that displaces at least one of the ion’s inner shell waters is indicative of an ion binding site at that location. Therefore, this site at the extracellular entrance is the first weak cation binding site in the channel.

Next, Na^+^ is pulled laterally into the extracellular vestibule by attractive forces originating from direct interactions with carbonyl and carboxylate groups on sidechains Q95 and E136 positioned just inside the pore mouth. The ion briefly sheds 1–2 waters from its inner hydration shell when coordinated by both Q95 and E136 simultaneously at CV ≈ -14 Å (S8 Fig). This interaction encourages Na^+^ to leave the binding site at the entrance and progress deeper into the channel instead of migrating back out into the extracellular medium. Once inside the vestibule, which ranges from CV ≈ -18.5 Å to -10 Å, the free energy gradually rises to ~ 8 kcal/mol as Na^+^ regains a full hydration shell and explores the walls of the wide aqueous cavity. The sidechain of residue E136 is highly mobile within the extracellular vestibule and maintains contact with Na^+^ within this space. The position of positively charged residue R159 forms a repulsive barrier that prevents the ion from passing between helices III and VII, and the very top of the vestibule is capped by a cluster of bulky aromatic and hydrophobic residues that block water and ions from exiting the pore vertically between helices I, II, III, and VII.

After passing by residue K132 at CV ≈ -9 Å, Na^+^ dissociates from E136 and interacts with the carboxylate sidechain of E162 via direct coordination at CV ≈ -8 Å, shedding 1–2 inner shell waters in the process. This interaction with E162 results in a very small ~ 1 kcal/mol dip in the free energy profile to a local minimum of ~ 7 kcal/mol. In contrast to the highly mobile E136 sidechain, the positions of sidechains E129, E162, and D292 in this region are not disrupted by the presence of the permeating ion and remain fixed in place by strong intramolecular interactions. From CV ≈ -6 Å to +6 Å, Na^+^ diffuses rapidly through the central region of the pore. The PMF steadily increases until reaching a global free energy maximum of ~ 13 kcal/mol at CV = -1.25 Å corresponding to the region of the central gate adjacent to the RSBH^+^ and between residues N297, V125, and A126. The ion keeps at least 4 waters in its inner hydration shell as it passes through this central constriction site (S8 Fig) where it is helped along by a direct interaction with the carbonyl oxygen on sidechain N297.

Close to the pore exit on the cytosolic side, the sidechain of E122 is also highly mobile and reorients its carboxylate group to face up and toward the ion as it approaches, as seen in the simulation snapshot in Fig 8B. Residue E122 directly binds Na^+^ at CV ≈ +4 Å resulting in another very shallow (~ 1 kcal/mol deep) local free energy minimum of ~ 8 kcal/mol. This interaction persists from CV ≈ +4 Å to +10 Å as sidechain E122 moves in step with the ion and flips downward toward the cytosol as the ion progresses. Na^+^ briefly sheds 1–2 coordinating waters around CV ≈ +8 Å as it travels through the ICG due to interactions with sidechains E121, E122, and H173 before finally exiting the pore and diffusing into the cytosol corresponding to CV > +15 Å. The PMF decreases steadily from its peak at CV = -1.25 Å back to the baseline of 0 kcal/mol as it enters the intracellular bulk solution past CV > +15 Å (Fig 8A). The free energy remains flat at 0 kcal/mol within the bulk solution until the edge of the reaction coordinate just beyond CV ≈ +20 Å, after which point the PMF rises sharply as the ion encounters the harmonic wall potential boundary (not shown).

Since Ca^2+^ takes the same route through the pore as Na^+^, the PMF for Ca^2+^ permeation (Fig 8A, purple line) is qualitatively very similar to Na^+^ but with higher local maxima and lower local minima because of the comparatively stronger electrostatic interactions between Ca^2+^ and negatively charged residues E136, E162, E122, and E121 (Fig 8). Also, like Na^+^, Ca^2+^ traverses the pore mostly hydrated with no less than six coordinating waters throughout much of the pathway (S8 Fig). The local minimum at CV ≈ -14 Å is the most dehydrating site for Ca^2+^ in the channel due to strong direct interactions with sidechains E136 and Q95 that briefly reduce the number of waters in the ion’s inner shell from seven to five (S8 Fig).

On the intracellular side, Ca^2+^ is coordinated by E122 and E121 simultaneously in a bidentate fashion at CV ≈ +11 Å, as shown in the snapshot in Fig 8C. This interaction results in a deep free energy well and global minimum of ~ -5 kcal/mol that marks another cation binding site in the channel. The charge complementarity between the two glutamate ligands and Ca^2+^, and the absence of a free energy well at this position in the Na^+^ PMF profile, suggests that this site may be selective for divalent cations over monovalent cations. After Ca^2+^ dissociates from this site, the PMF returns to the reference value of ~ 0 kcal/mol within the bulk solution on the cytosolic side corresponding to CV > +15 Å.

The maximal overall free energy barrier (from global minimum to global maximum) found for Na^+^ permeation is ~ 15.5 kcal/mol. Na^+^ overcomes this large central barrier in steps by crossing a series of smaller barriers separated by short distances of ~2–3 Å. Each step is a new position in the channel where the free energy differs by only a few kcal/mol from the previous position. A look at the plotted trajectories of the biased Na^+^ ions for this system given in S9 Fig show that this is a very efficient mechanism that allows Na^+^ to readily permeate the channel.

As shown in Fig 8, the global free energy maxima of both the Ca^2+^ and Na^+^ PMFs occur in the same position along the channel at CV = -1.25 Å, which corresponds to the space adjacent to the RSBH^+^ between sidechains N297, A126, and V125. However, there is an additional barrier in the Ca^2+^ PMF profile starting at CV ≈ -9 Å which has a height of ~ 12 kcal/mol that is not present in the Na^+^ PMF. At this location, fully hydrated Ca^2+^ attempts to pass through a narrow constriction site where its movement is partially opposed by hydrophobic residues A126 and V125 along one side of the pore wall. The carbonyl oxygen on sidechain N297 weakly coordinates Ca^2+^ to help shed one water molecule from the ion’s densely packed hydration shell, but it is unable to shed a second due to calcium’s strong hydration free energy. The pore is still wide enough for the ion and its coordinating waters to squeeze by, but these factors create a steep barrier that slow down Ca^2+^ diffusion substantially. Looking at the plotted trajectories for this system in S9 Fig, the Ca^2+^ walker sampling this region took about ~100 ns to cross this barrier. In contrast, the Na^+^ walkers went through the same region in no more than ~ 5 ns, which illustrates the magnitude of the difference in conduction of Ca^2+^ vs. Na^+^ through the channel.

Furthermore, the relative overall barrier heights between the two cation species show that Ca^2+^ must overcome a free energy barrier that is ~7 kcal/mol higher than that for Na^+^ to permeate the channel from the extracellular side. Taken together with the slower diffusion rate for Ca^2+^, these results signal a much stronger preference for Na^+^ transport over Ca^2+^ than expected based on an experimental permeability ratio of *P*_Ca_/*P*_Na_ = 0.3 from reversal potential measurements, which would correspond to a theoretical free energy difference of less than ~ 1 kcal/mol.

This degree of overestimation of the barrier heights is not uncommon when employing nonpolarizable additive force fields, such as the C36m force field used here, to describe biophysical processes in complex electrostatic environments, especially for those involving Ca^2+^ [78, 79]. As demonstrated for the extensively studied gramicidin A channel, the magnitudes of the calculated cation-protein interaction energies in confined spaces depend heavily on the system configuration and force field used [80]. It is likely that the inclusion of additional factors, such as the effects of polarization or multiple occupancy within the channel, are needed to obtain absolute barrier height estimates that are more quantitatively in line with experimental single-channel permeability measurements. Each of these factors can affect barrier heights and well depths relative to bulk by several kcal/mol in highly charged systems such as ours [81].

However, here we are primarily interested in investigating the *trends* in relative selectivity observed among the wild-type C1C2 and mutant channels, and inclusion of these factors is not expected to drastically alter the overall *relative* relationship between Na^+^ and Ca^2+^ permeation in this channel to the extent that it would change the conclusions of the present study. Therefore, the use of the more computationally efficient nonpolarizable C36m additive force field is appropriate for our purposes. Qualitatively, the present PMF profiles agree well with the expected selectivity of C1C2 based on our electrophysiology results, which showed a higher relative permeability for Na^+^ versus Ca^2+^ in the wild-type channel.

#### Pore structure and differential selectivity of the two open states

**Fig 9:**
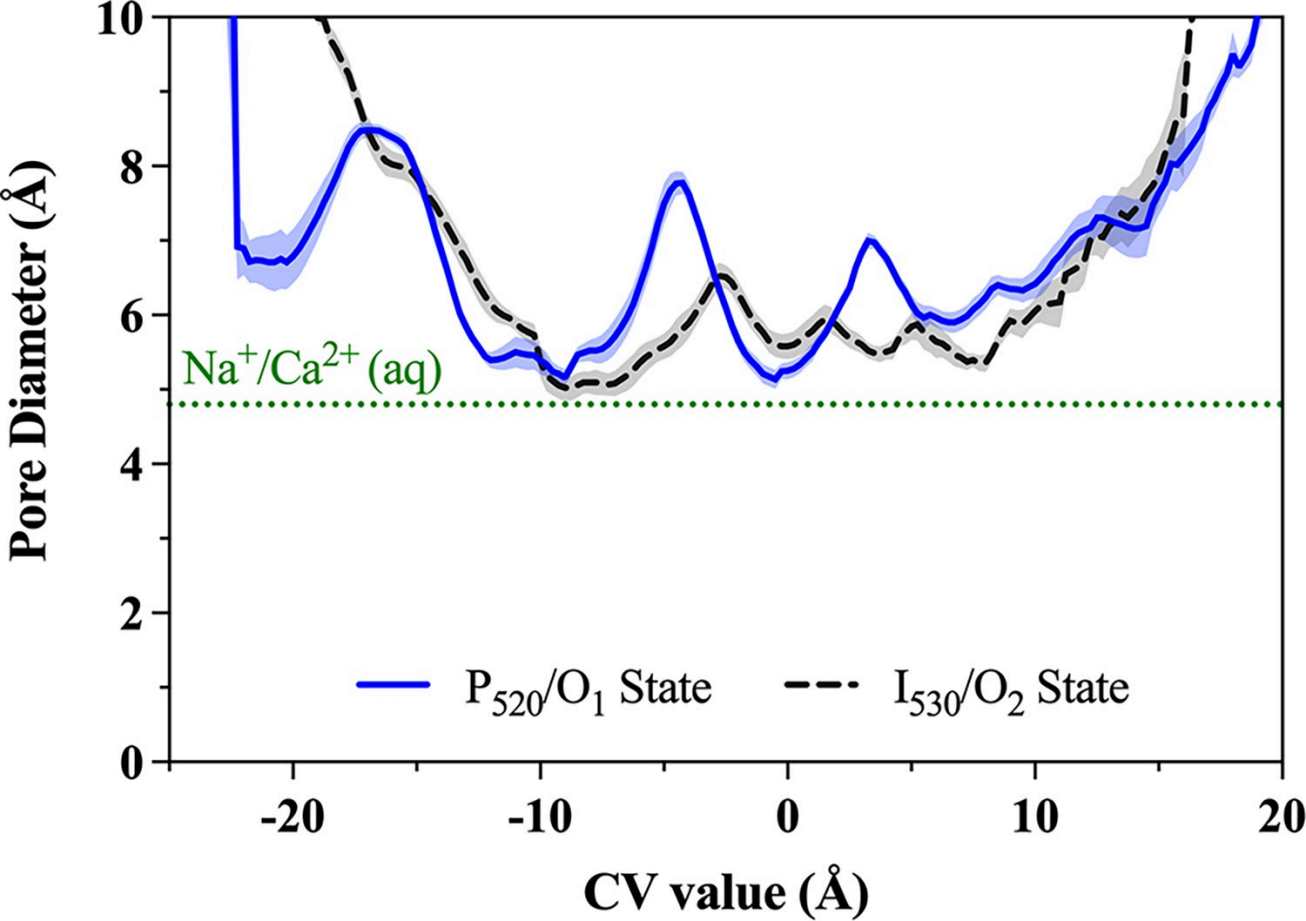
Comparison of pore size in the two open states. The average pore diameter during Na^+^ translocation is plotted as a function of the CV value for the wild-type C1C2 channel in the P_520_/O_1_ open state (solid blue line) and the I_530_/O_2_ open state (gray dashed line). Pore dimensions were measured with the software program HOLE as described in the data analysis section of the Methods. Shaded areas are ± SEM, where n = 8. The green dotted line at 4.8 Å marks the diameter of hydrated Na^+^ or Ca^2+^ defined as twice the peak of the radial distribution of the ion’s first hydration shell.

In our current model of the wild-type C1C2 channel, the minimum pore diameter measured from ensemble-averaged structures during Na^+^ permeation was found to be nearly identical for both open states at 5.1 ± 0.1 Å in the P_520_/O_1_ state and 5.0 ± 0.2 Å in the I_530_/O_2_ state (Fig 9), and was just slightly larger than the 4.8-Å diameter of Na^+^ and Ca^2+^ with a full inner hydration shell [82]. However, these values likely underestimate the pore’s true effective size since they neglect the flexibility and anisotropy of the channel’s interior walls. In contrast to the rigid backbone-lined pore structures of many tetrameric ion channels, pore boundaries in C1C2 are dynamic and largely defined by sidechains with high conformational freedom that may easily reposition to accommodate the passage of larger cations.

Furthermore, the cross-sectional area of the pore is not cylindrical but rather forms a slot shape with dimensions ~ 5 Å x 10 Å in the region of the central gate- large enough to fit two hydrated Na^+^ side by side. These results show that the present open state models are in excellent agreement with our lab’s empirically-derived estimate of ChR pore size reported previously [41], and is consistent with the known size cut-off for the permeability of large organic cations in ChRs [83].

Unlike in ChR2, the selectivity differences between the two open states of the wild-type C1C2 channel are miniscule. Results from our reversal potential measurements presented earlier indicate a small but statistically significant decrease in the apparent H^+^ permeability relative to Na^+^ in the I_530_/O_2_ open state versus the P_520_/O_1_ open state, whereas the relative permeabilities of K^+^ and Ca^2+^ vs. Na^+^ remained the same. Alternatively, since these are relative measures of permeability and not absolute values, this could also be caused by a nonspecific increase in cation permeability in the I_530_/O_2_ open state, while H^+^ permeability remains the same in both open states.

To clarify the nature of these selectivity differences, and to better understand the structural and mechanistic basis for the channel’s reduced conductance in the I_530_/O_2_ open state, we compared the PMF profiles of Na^+^ and Ca^2+^ permeation through both open states of the wild-type C1C2 channel. Results from MWWT-MetaD simulations show that the lowest free energy path for cations through the pore is the same in both open states. However, the free energy surfaces of Na^+^ and Ca^2+^ permeation along this route in the low-conducting I_530_/O_2_ open state differ dramatically from those in the P_520_/O_1_ open state.

**Fig 10:**
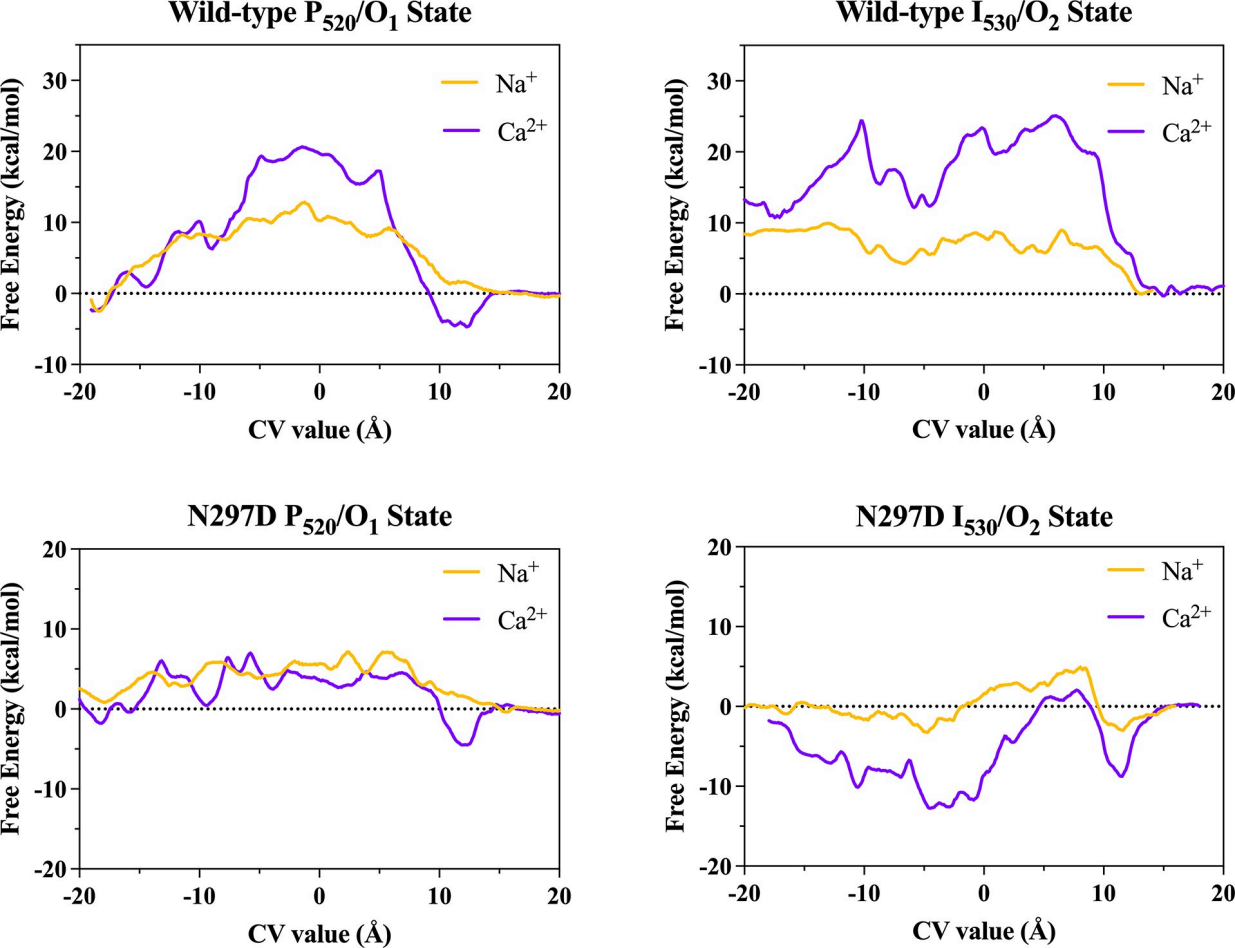
Comparison of PMF profiles for cation permeation in the high- and low-conducting open states of wild-type C1C2 and N297D mutant channels. Potential of mean force (PMF) profiles calculated from MWWT-MetaD simulations of Na^+^ (yellow line) and Ca^2+^ (purple line) translocation through the indicated structure. For reference, the channel entrance on the extracellular side corresponds to CV ≈ -18 Å, and positive CV values >15 Å correspond to the bulk cytosolic solution.

The top two panels in Fig 10 compare the free energy profiles between the two open state structures of the wild-type channel. In stark contrast to the results for the P_520_/O_1_ open state structure, the PMF for Na^+^ permeation in the I_530_/O_2_ open state reveals a mostly flat and featureless free energy surface throughout much of the channel. The PMF for Ca^2+^ transport shows the same general pattern as Na^+^, where the main features of the free energy surface occur in the same locations along the reaction coordinate, but the relative magnitudes of the minima and maxima are larger for the Ca^2+^ PMF due to its +2 ionic charge. Remarkably, the relative differences between the main barrier heights for Na^+^ vs. Ca^2+^ permeation are the same in both open states and therefore have similar permeability ratios, in excellent agreement with the experimentally observed trends for these two cations in the wild-type channel.

Next, we examined how the major cation-protein and cation-water interactions correlate to the PMF and found that the observed changes to the free energy landscape in the I_530_/O_2_ open state can be attributed to specific differences in pore structure at two locations: the extracellular entrance and the central binding site. The structure of the I_530_/O_2_ open state shows an additional outward bend of the extracellular end of helix II that is not observed in the P_520_/O_1_ open state. As a result, the distance between the tops of helices I and II at the extracellular entrance increases by several angstroms, causing partial destruction of the weak cation binding site that forms there in the P_520_/O_1_ open state.

This change in pore structure can be easily seen by comparing the pore dimensions of the two open states, shown in Fig 9 for the wild-type channel, near the region corresponding to the extracellular entrance around CV ≈ -20 Å. On the one hand, this eliminates a free energy well close to the membrane surface that might attract cations to the pore entrance. On the other hand, it also shortens the total length of the pore and flattens the PMF for the first ~ 8 Å of the pathway inside the extracellular vestibule from CV ≈ -20 to -12 Å, creating an essentially barrierless entry point for cations from the extracellular side.

Instead of participating in the ECG, the carboxylate sidechain of residue E140 now faces outward to interact with the polar lipid headgroups. In this position, E140 has a great deal of conformational freedom and can still singly-coordinate cations diffusing near the membrane surface to pass them along to E136, but the strength of this interaction is much weaker compared to the dual-ligand binding site formed by N92 and E140 working together in the previous open state. Therefore, the first weak coordination site for cations in the I_530_/O_2_ open state channel then becomes the site formed by sidechains Q95 and E136 located further down the gap between helices I and II around CV ≈ -12 Å.

The second difference arises as a consequence of the different proton transfer reactions that take place during channel opening in the *syn*-cycle prior to the onset of cation conductance that leads to the presence of deprotonated E129 and D292 in the I_530_/O_2_ open state. This introduces two additional negative charges in the pathway and also increases the conformational mobility of these two sidechains which were both fixed in place by hydrogen bonds in the previous open state. The combination of these two factors leads to a broad free energy well in the PMFs of both cations over the range CV ≈ -10 to 0 Å, with a well depth of ~ 5.5 kcal/mol for Na^+^ and ~ 12 kcal/mol for Ca^2+^. The free energy minimum at CV ≈ -6 Å in the Na^+^ PMF correlates to dual bidentate coordination by E162 and D292 carboxylates.

**Fig 11:**
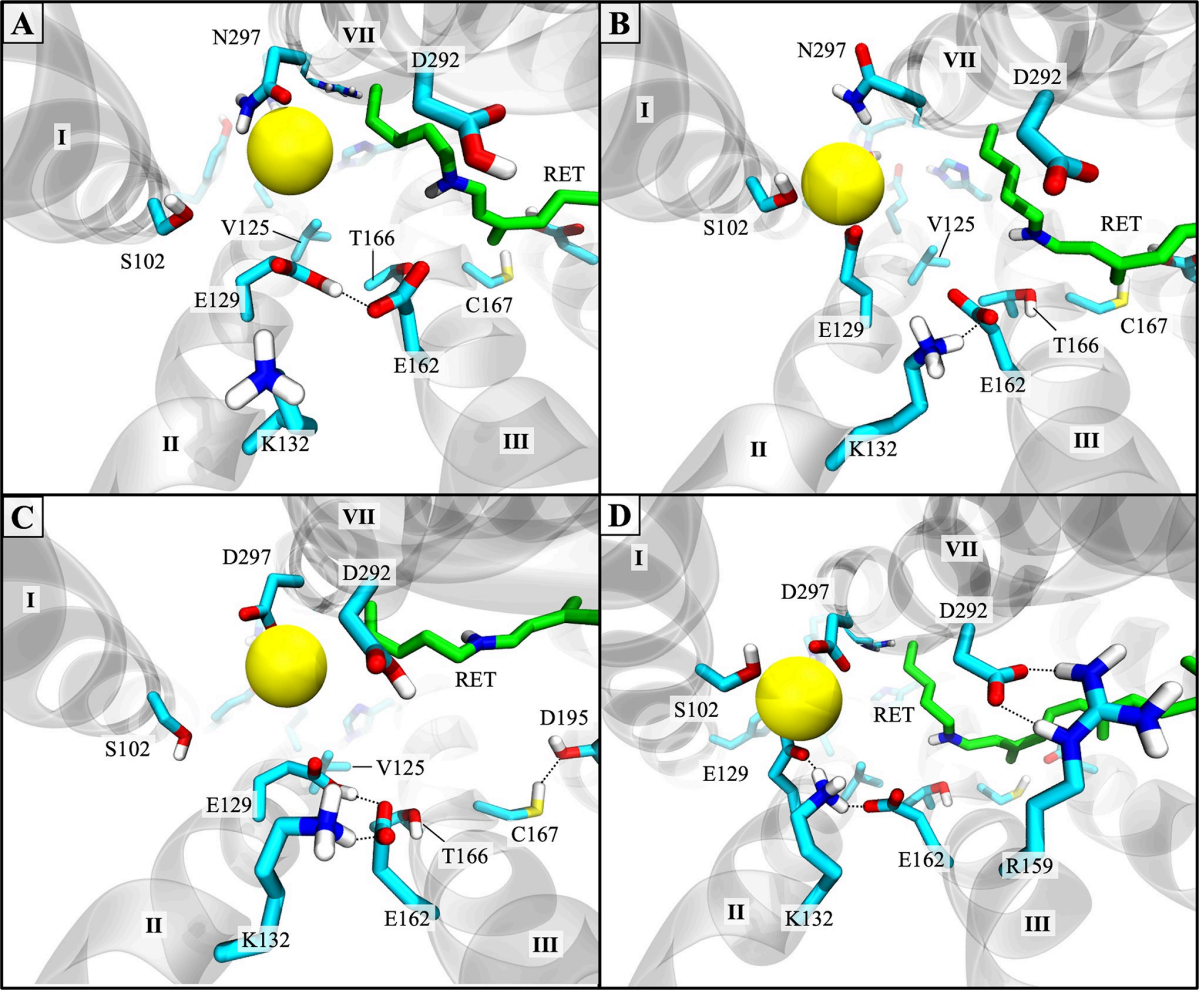
Charge density and number of coordinating ligands in central gate governs selectivity. View of Na^+^ passing through the central gate of **(A)** wild-type C1C2 in the P_520_/O_1_ open state, **(B)** wild-type C1C2 in the I_530_/O_2_ open state, **(C)** N297D in the P_520_/O_1_ open state, and **(D)** N297D in the I_530_/O_2_ open state. Results for Ca^2+^ coordination were similar to Na^+^ except for in the low-conducting I_530_/O_2_ open states (B and D), where Ca^2+^ associated strongly with the charged D292 sidechain instead of S102, as discussed in the text. View is from the extracellular side of the channel and corresponds to CV ≈ -2 Å. Black dotted lines indicate hydrogen bonds, the protein backbone is in transparent gray cartoon, select residues are shown as sticks in cyan, retinal is in green, and yellow spheres are Na^+^. The other protomer, solvent, lipids, and nonpolar protons are hidden for clarity.

For Na^+^, these differences allow the progressive shedding of water molecules from the ion’s inner hydration shell in a smooth and continuous manner until only one shell water is left, which occurs in the central binding site corresponding to CV ≈ -2 to 0 Å. Here, the polar charged/polar oxygen sidechains of three residues E129, N297, and S102 all directly coordinate the ion simultaneously. Fig 11 shows how the structure of this binding site is affected by the introduction of a negative charge on E129 to facilitate stronger Na^+^ binding with three ligands in the I_530_/O_2_ open state (panel B) versus one neutral ligand in the P_520_/O_1_ open state (panel A).

Two additional strongly dehydrating binding sites for Na^+^ were found near the pore exit on the cytosolic side. The first site is between helices II and III due to strong interactions with residues E121, E122, H173, and N182 and was also identified as an exit for both cations in the previous open state. The second site, which was not identified as a binding site in the P_520_/O_1_ open state, is located between helices I and VII and formed by sidechains Y109, Q112, E122, and H304. Both sites correspond to CV ≈ +9 Å but are separated by a lateral distance of ~ 5 Å at the cytosolic side.

The primary interactions are somewhat different for Ca^2+^ due to the ion’s +2 charge. For Ca^2+^, the free energy minimum occurs around CV ≈ -5 Å and correlates to triple coordination by all three negatively charged carboxylate sidechains E129, E162, and D292 in a mixed monodentate-bidentate manner. The strong binding interaction between Ca^2+^ and the negative charge on D292 pulls the ion close to the RSBH^+^ as it travels between the free energy minimum at CV ≈ -5 Å and the central constriction site around CV ≈ -2 to 0 Å. As a result, Ca^2+^ coordination in this space is dominated by strong direct interactions with charged E129 and D292 and transient direct coordination by polar N297 working together to partially dehydrate the ion to a minimum of 6 waters in its inner hydration shell, whereas residue S102 interacts with the passing ion only through bridging water molecules. The very steep rise in free energy on either side of the PMF minimum indicates strong Ca^2+^ binding at this site that could cause the ion to become trapped and lead to markedly slower transport or even partial pore block. Indeed, the walker trajectories for Ca^2+^ in this structure (S9 Fig) show two walkers that were unable to leave the binding site within the 100-ns simulation timeframe.

For both cations, the presence of additional negative charges on E129 and D292 in the I_530_/O_2_ open state increases the cation binding affinity of this site vs. the P_520_/O_1_ open state and reduces the free energy barriers by partially counteracting the unfavorable energic effects of adjacent hydrophobic residues A126 and V125 that oppose water and ion passage within the central constriction of the pore. In contrast to the P_520_/O_1_ open state, the PMF of Na^+^ permeation in the I_530_/O_2_ open state is weakly correlated to the ion’s hydration number (S8 Fig), indicating that the ability of the channel to efficiently dehydrate cations is an important driver of the transport mechanism in this state. Inspection of the walkers’ trajectories (S9 Fig) reveals that the mobility of ions within the pore is somewhat reduced for Na^+^ and severely reduced for Ca^2+^ in this state compared to the P_520_/O_1_ open state, indicating that the dehydration process within the pore is much more efficient for Na^+^ compared to Ca^2+^.

Collectively, the results suggest that ion dehydration and dissociation from binding sites are likely rate-limiting steps in the permeation mechanism and lead to reduced overall conductance in the I_530_/O_2_ open state, in agreement with experimental observations. They also help to clarify that the apparent reduction in the permeability ratio *P*_H_/*P*_Na_ measured at steady-state current versus peak current in the wild-type channel is likely due to a nonspecific increase in cation selectivity relative to H^+^ in a manner that leads to slower single-ion transport rates and reduced current through the low-conducting I_530_/O_2_ open state.

#### Effect of the N297D mutation on the energetics of cation transport

Next, we investigated the mechanism by which replacement of neutral asparagine at position 297 in the region of the central gate with a negatively charged aspartate residue leads to increased Ca^2+^ selectivity relative to Na^+^ in C1C2. Results from MWWT-MetaD simulations in N297D show that the lowest free energy route for both cations through the mutant channel is the same as in the wild-type channel. Therefore, the location of the pathway itself is not altered by this mutation. The minimum pore size is also not drastically different in the N297D mutant compared to the wild-type channel (S10 Fig).

*High-conducting P*_*520*_*/O*_*1*_ *open state*. Calculation of the PMF profiles for Na^+^ and Ca^2+^ permeation along the minimum free energy route in the high-conducting P_520_/O_1_ open state show that the overall shapes of the free energy landscapes resemble that of the P_520_/O_1_ open state of the wild-type channel, but the height of their main free energy barriers are greatly reduced for both cations in N297D, as shown in Fig 10. Notably, the mutation reduced the barriers to a greater extent for Ca^2+^ permeation than for Na^+^ permeation through the same regions.

For both cations, the largest reduction in free energy occurs in the region of the central constriction site where the mutant residue is located, spanning from CV ≈ -6 to +6 Å. In the Na^+^ PMF, the global peak free energy barrier is reduced from ~ 15 kcal/mol in the wild-type channel to just ~ 6 kcal/mol in the N297D mutant channel. The effect on the Ca^2+^ PMF is even larger, lowering the barrier from ~ 23 kcal/mol to ~ 9 kcal/mol. Importantly, this makes the barrier heights of Na^+^ and Ca^2+^ almost equal with respect to the bulk cytosolic solution, and differ by only ~ 3 kcal/mol coming from the extracellular side in the N297D mutant vs ~ 7 kcal/mol in the wild-type channel.

Inspection of the hydration shell data for cations in this structure (S8 Fig) shows that dehydration within the pore is more efficient for both Na^+^ and Ca^2+^ at key binding sites compared to the wild-type channel, but it is especially improved for Ca^2+^ within the central binding site between CV ≈ -5 Å and 0 Å. Here, Ca^2+^ coordination is enhanced by the negatively charged carboxylate on D297 that allows the more favorable bidentate interaction with Ca^2+^ versus the weaker monodentate interaction with neutral asparagine in the wild-type channel. However, since there is still only one participating ligand at this site (see Fig 11C), this increases the electronegativity and the binding strength of this site enough to partially counteract the barrier imposed by V125 and A126 without creating a deep free energy well that could lead to slowed transport or partial pore block. As shown by the plots of the ion trajectories along the channel axis (S9 Fig), Na^+^ diffusion is slowed only marginally in the P_520_/O_1_ open state while Ca^2+^ movement is not hindered relative to the wild-type channel. These results indicate a large increase in Ca^2+^ permeability relative to Na^+^ in the N297D mutant vs. the wild-type channel in the high-conducting P_520_/O_1_ open state consistent with the trends identified from experimental measurements.

*Low-conducting I*_*530*_*/O*_*2*_ *open state*. In contrast to the wild-type channel, our electrophysiology measurements presented earlier showed that the relative permeabilities of Na^+^ and Ca^2+^ (*P*_Ca_/*P*_Na_) for the N297D mutant differed significantly when measured at peak current vs. steady-state current, where *P*_Ca_/*P*_Na_ was increased to a lesser extent by the N297D mutation in the low-conducting I_530_/O_2_ open state vs. the high-conducting P_520_/O_1_ open state. The calculated PMF profiles shown in Fig 10 are consistent with this trend, where the difference between the maximum free energy barriers between the two cations for the N297D channel is greater in the I_530_/O_2_ open state (|ΔΔG| ≈ 5.5 kcal/mol) than in the P_520_/O_1_ open state (|ΔΔG| ≈ 3 kcal/mol) but still less than either state of the wild-type channel (|ΔΔG| ≈ 7.5 kcal/mol).

Similar to the results for the high-conducting P_520_/O_1_ open state, the PMFs for cation permeation in the I_530_/O_2_ open state of the N297D mutant channel also show a marked reduction in the total free energy of transport for both Na^+^ and Ca^2+^ as a result of the mutation, while the overall shapes of their free energy surfaces share many of the same features as the PMFs of the wild-type channel in the same state. During the transition to the low-conducting open state, the N297D mutant channel exhibits the same local distortion of the helix II backbone as the wild-type protein that partially destroys the binding site at the extracellular entrance between the ends of helices I and II.

Although cations still entered the extracellular vestibule by following the negatively charged carboxylate residues along helix II, the distortion creates a nearly barrierless entry point to the pore in the mutant channel for both ion types from the extracellular side. For Na^+^ permeation, the resulting free energy surface is very flat and remains close to zero kcal/mol throughout the entire pathway with respect to the cytosolic bulk solution. This condition permits Na^+^ to freely diffuse from one side of the pore to the other, as seen in the walkers’ trajectories plotted in S9 Fig, indicating that Na^+^ transport in the I_530_/O_2_ open state in N297D is just as efficient as for the high-conducting P_520_/O_1_ open state of the wild-type channel.

Likewise, the PMF profile for Ca^2+^ permeation shows the same overall shape and major features as the Na^+^ PMF, but the free energy is lowered several times more for Ca^2+^ by the N297D mutation than for Na^+^ such that the total PMF for Ca^2+^ is far below that for Na^+^ with respect to the cytosol. As shown in Fig 10, the Ca^2+^ PMF now features a very broad and deep free energy well that spans nearly the entire length of the pore from CV ≈ -20 Å at the membrane surface on the extracellular side to just before the channel exit on the cytosolic side around CV ≈ +8 Å. This well reaches a global minimum of ~ -13 kcal/mol with respect to the cytosolic bulk solution at the same point along the pathway as in the wild-type channel PMFs (CV ≈ -5 Å), and correlates to strong electrostatic interactions between Ca^2+^ and the negatively charged sidechains of E129, E162, and D292.

As mentioned earlier, in the wild-type channel, the presence of these three negative charges concentrated in the central region of the pore created a deep free energy well with steep ~ 12 kcal/mol barriers on either side, substantially reducing the rate of Ca^2+^ transport in that structure. Curiously, the introduction of a fourth negative charge in the same region by the N297D mutation results in a similarly deep free energy well in the PMF but does not lead to slow transport. In fact, Ca^2+^ is seen traversing the channel even more efficiently than in any of the other structures studied (see S9 Fig).

**Fig 12:**
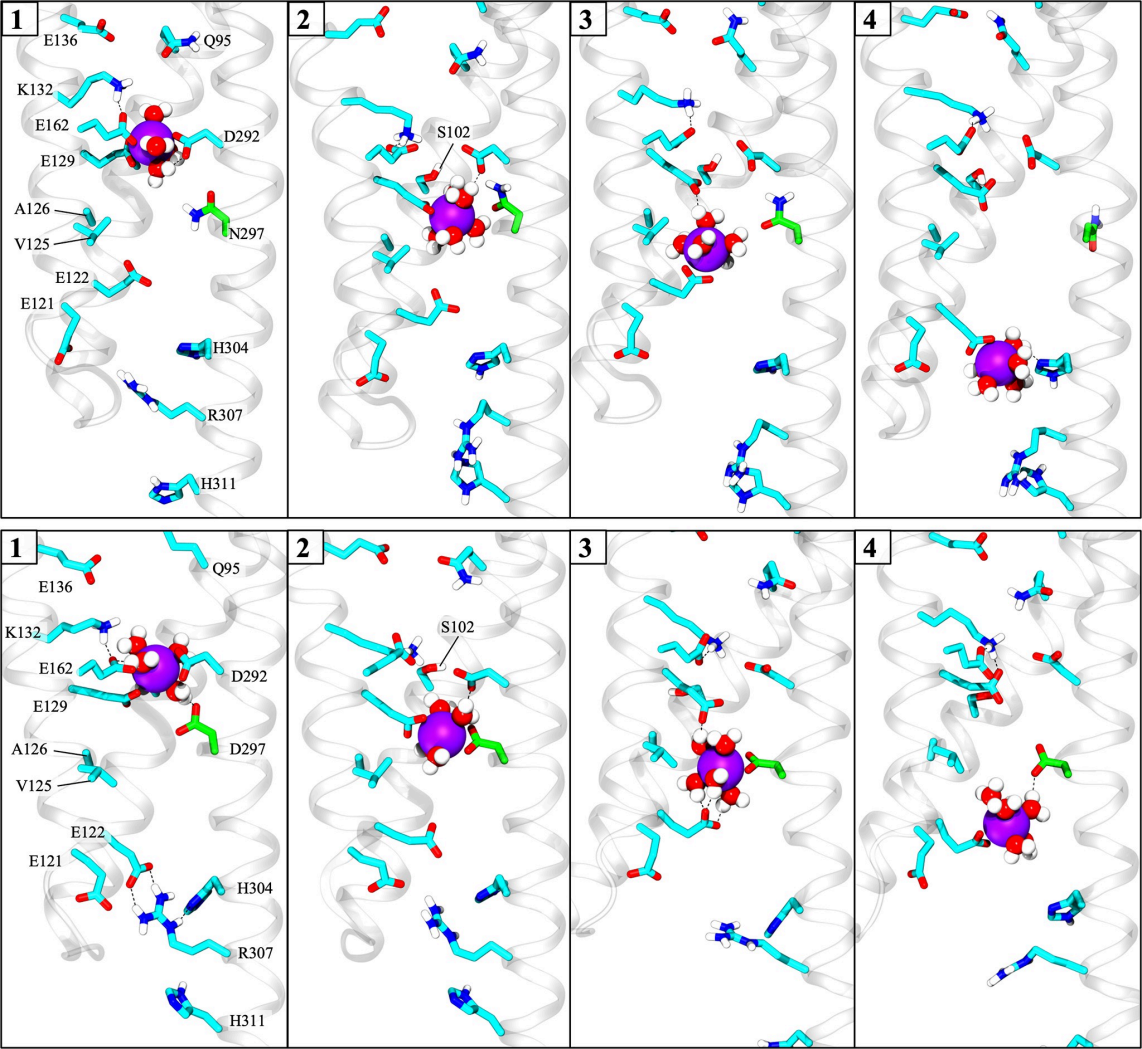
Sidechain D297 mediates Ca^2+^ transfer between central and cytosolic binding sites. Shown above is a series snapshots from MWWT-MetaD simulations comparing the mechanisms of Ca^2+^ permeation between the wild-type channel (top row) and the N297D mutant (bottom row) in the I_530_/O_2_ open state as the ion travels from the free energy minimum in frame 1 (CV ≈ -5 Å) to the pore exit on the intracellular side in frame 4 (CV ≈ +10 Å). All views are taken from the channel’s interior facing helix I and oriented with the extracellular side at the top of the image. Only transmembrane helices I, II, and VII and relevant sidechains are visible, while the remainder of the protein and the retinal at position 296 are hidden for clarity. In all parts of the figure, the protein backbone is in transparent gray cartoon, the sidechain at position 297 is shown as green sticks while all other sidechains are colored cyan, black dotted lines represent hydrogen bonds, purple spheres are Ca^2+^, and waters belonging to the ion’s inner hydration shell are shown as red and white space-filling molecules. The other protomer, nonpolar protons, bulk solvent, other pore waters, and membrane lipids are hidden for clarity.

*The distribution of negative charges in the pore affects Ca*^*2+*^ *mobility*. To understand how Ca^2+^ can escape the central binding site within the simulation time frame in the N297D mutant but not in the wild-type channel, we compared the individual steps involved in transporting Ca^2+^ across the central barrier in these two proteins in the I_530_/O_2_ open state. The top row of Fig 12 shows the Ca^2+^ transport process for the wild-type channel described earlier, where the ion travels from the free energy minimum in frame 1 to the pore exit on the intracellular side in frame 4.

Notice that residue E122 is located two helix turns below residue E129 on the same transmembrane segment (helix II) with barrier-forming residues V125 and A126 on the helix turn in between. This arrangement places E122 and E129 too far apart for both sidechains to coordinate the ion simultaneously. Therefore, as Ca^2+^ passes by hydrophobic residues V125 and A126, Ca^2+^ briefly becomes fully rehydrated and then partially dehydrated again during the handoff between E129 and E122. Considering the relatively slow substitution rate of inner shell waters around Ca^2+^ compared to Na^+^ [12], these repeated dehydration-rehydration cycles are likely rate-limiting steps in the mechanism that would slow Ca^2+^ passage through the pore. Furthermore, the strength of the electrostatic interaction between Ca^2+^ and the neutral sidechain of N297 is very weak, so N297 contributes very little stabilization energy to the ion during the transition. The combined effects of all the aforementioned factors results in a large and sudden ~ 12 kcal/mol increase in the free energy as Ca^2+^ travels through this region in the wild-type channel.

The steps of the Ca^2+^ transport mechanism through the same region in the N297D mutant channel are shown in the second series of snapshots in Fig 12. As shown in the figure, while residues E129, E162, and D292 also coordinate Ca^2+^ to form a binding site in the N297D mutant (frame 1), the position of the negative charge on D297 leads to a more even distribution of negative charges along the pore’s axis. Specifically, the D297 sidechain is situated on transmembrane helix VII directly across from barrier-forming residues V125 and A126 on helix II, and halfway between E129 of the central binding site and E122 of the cytosolic binding site. In this staggered arrangement, residue D292 competes for binding to Ca^2+^ with residue D297 one helix turn below.

This competition encourages the dissociation of Ca^2+^ from the binding site as the substitution of the D292 ligand by D297 pulls the ion down and away from residue E162. The sidechain of E129 swings down to stay directly coordinated to Ca^2+^ as this substitution is made and forms a secondary binding site with D297 about ~ 4 Å below the first, as shown in frame 2. Next, E129 passes the ion to D297, which in turn swings down to carry Ca^2+^ past barrier-forming residues V125 and A126, as shown in frame 3, and delivers the ion directly to E122 at the pore exit in frame 4. Lastly, sidechain E122 swings down to carry Ca^2+^ to E121 where it is released into the cytosolic bulk solution in a manner similar to that shown in frame 4 for the wild-type channel.

In this way, residue D297 in the mutant channel reduces the amount of free energy required for Ca^2+^ to leave the binding site and assists the passage of Ca^2+^ across the largest free energy barrier in the channel by stabilizing its transition between sidechain E129 in the central binding site and E122 at the pore exit on the intracellular side. Therefore, while the absolute magnitude of the free energy basin in of the binding site is the same in both proteins, the N297D mutation decreases the rate that the free energy rises as Ca^2+^ moves through the central constriction site.

The more gradual increase in free energy allows Ca^2+^ to overcome the barrier more easily by breaking it down into smaller steps, where each step is a new position in the channel that differs by only a few kcal/mol from the previous position, thereby preventing the ion from becoming trapped. These results show that Ca^2+^ permeability in C1C2 is controlled not only by the overall electronegativity of the pore, but also by the *distribution* of negative charges and their local environment within the pore.

## Conclusion

In this work, we have uncovered the major energetic, mechanistic, and structural factors governing Na^+^ and Ca^2+^ permeation in both open states of the light-activated channelrhodopsin chimera C1C2 at the atomic level using complementary experimental and computational methods. Electrophysiological characterization of whole-cell photocurrents measured in *X*. *laevis* oocytes revealed the Ca^2+^ permeability of the wild-type C1C2 channel relative to Na^+^ is on par with the high Ca^2+^-conducting ChR variant CatCh [17], and that the N297D mutation in the central region of the pathway increases the relative Ca^2+^ permeability of C1C2 even further by nearly two-fold. These measurements also found that the magnitude of the enhancement was larger for photocurrents measured at peak current compared to steady-state current, implying that the selectivity of the two open states were differentially impacted by the N297D mutation.

To understand the specific cation-protein and cation-water interactions underlying these experimental observations, we next sought to calculate the PMF profiles of Na^+^ and Ca^2+^ permeation through both open states of the wild-type C1C2 channel and the N297D mutant. However, since no high-resolution crystal structure of the fully open channel for any channelrhodopsin protein has been solved yet, we performed extensive molecular modeling studies that led to the successful development of four dimeric open state structures with continuous water-filled pores suitable for further investigation in ion permeation studies: 1) wild-type C1C2 high-conducting P_520_/O_1_ open state 2) N297D high-conducting P_520_/O_1_ open state 3) wild-type C1C2 low-conducting I_530_/O_2_ open state, and 4) N297D low-conducting I_530_/O_2_ open state. A thorough analysis of the results show that our new models are in excellent agreement with the experimentally observed changes to protein conformation and helix hydration associated with the onset of cation conductance in C1C2 [34, 35, 37].

Considering the uncertainties regarding the putative permeation pathway in C1C2, we chose to simulate Na^+^ and Ca^2+^ permeation through the new model structures using MWWT-MetaD simulations. This method enabled us to determine the lowest free energy route for cations through the channel and simultaneously calculate the 1D-PMF profiles for single-ion transport along it, without placing any restraints on the protein or on the ion’s position while inside the channel. The results found that while Na^+^ and Ca^2+^ primarily followed the series of carboxylate sidechains along helix II as expected, the entrance to the pore for cations on the extracellular side is actually located in a gap between the extracellular ends of helices I and II and not though the extracellular access tunnel originally predicted from the crystal structure of the closed state C1C2 channel [21]. Here, the sidechains of residues K88, N92, E140, and Y137 form a weak cation binding site in the P_520_/O_1_ open state that attracts water and cations that diffuse near the membrane to the pore mouth. These residues create an electronegative surface more suitable for attracting cations into the pore compared to the slightly electropositive surface of the extracellular access tunnel.

The remainder of the permeation pathway was in line with the predicted route where the pore is bounded by helices I, II, III, and VII and passes through the space adjacent to the RSBH^+^. Results were consistent across all replicas and in all four channel models studied. Both Na^+^ and Ca^2+^ followed the same route through the pore except for the channel exit on the cytosolic side, where Na^+^ generally preferred to exit under the first intracellular loop or under helix I, and Ca^2+^ showed a strong preference for the exit between helices II and III.

It is important to note that in addition to the water in the main pathway, multiple continuous columns of interconnected water molecules were observed moving through regions of the protein that were inaccessible to Na^+^ and Ca^2+^. For instance, water flowed through the cavity that formed between the bend in the retinal polyene chain and helix VI during all MWWT-MetaD simulations. Water also persistently flowed through the extracellular access tunnel to join with this water column and with the water in the main pathway, as illustrated by the snapshots in S3 Fig. Therefore, since H^+^ transport is thought to occur via a “water wire” or Grotthuss-type mechanism, we cannot rule out the possibility that H^+^ may take one or multiple routes through the pore, and that these may be separate and distinct from the pathway for larger cations. The specific role this water plays in channel function would be an interesting question for future studies to address.

Analysis of the ion-water and ion-protein intermolecular interactions involved in cation transport showed that cations and water molecules are co-permeant. Cations migrate through the pore in a partially dehydrated state using a relay mechanism, whereby cations are passed between adjacent carboxylate residues on helices II and VII via sidechain swinging in a manner reminiscent of a monkey swinging on vines. In this way, cation permeability is controlled by a series of low-affinity asymmetrical binding sites with highly flexible sidechains along the full length of the pore.

Furthermore, we found that the differential selectivity and conductance properties between the high- and low-conducting open states are caused by specific changes to pore conformation that occur during channel opening in the *syn*-cycle and impact the cation binding affinities of the central binding site and the pore entrance binding site in the low-conducting I_530_/O_2_ open state. As discussed in the introduction, the channel undergoes different proton transfer reactions following photoactivation in the *syn*-cycle vs. the *anti*-cycle that leads to the presence of two additional negative charges in the central region of the pore (specifically on E129 and D292) in the I_530_/O_2_ open state. In the wild-type channel, this increased the strength of cation binding at the central binding site which lowered the free energy barrier of transport for both Na^+^ and Ca^2+^ by equal amounts but also slowed the diffusion rate of ions through the channel.

We also observed a distortion in the helix II backbone in the I_530_/O_2_ open state that caused the extracellular ends of helices I and II at the pore entrance to separate, partially destroying the binding site that formed there in the P_520_/O_1_ open state. Although this eliminated a free energy well on the extracellular side that would help to attract cations to the pore entrance, cations still passed between helices I and II to enter the pore during the simulations even though the strength of the binding site was significantly reduced.

Simulations in the N297D mutant channel showed that the N → D mutation increased both Na^+^ and Ca^2+^ permeability by mediating the handoff of cations between the central and cytosolic binding sites via the direct coordination of cations by D297 and sidechain swinging. This reduced the maximum free energy barrier for both cations imposed by hydrophobic residues V125 and A126 that separate the two sites and line the narrowest region of the pore. However, the effect was greater for Ca^2+^ due to its +2 charge and higher free energy of hydration versus Na^+^, leading to increased Ca^2+^ permeability relative to Na^+^ in the mutant channel. Overall, results from the MWWT-MetaD simulations of Na^+^ and Ca^2+^ permeation across all four open state C1C2 models developed here successfully reproduced the trends in cation selectivity and conductance properties observed from experimental measurements of wild-type C1C2 and N297D mutant channel photocurrents.

## Supporting information

S1 Fig C1C2 dimer and simulation box setup. Snapshot of the unequilibrated system used in molecular dynamics simulations containing the wild-type C1C2 dimer embedded in a DOPC lipid bilayer with explicit water and 150 mM NaCl. Protomer A is in blue and protomer B is in red. Lipids are represented as space-filling molecules with foreground lipids hidden for clarity. Water molecules are rendered as blue-gray transparent surfaces on either side of the membrane. Yellow spheres are Na^+^. Cyan spheres are Cl^-^. The simulation box measured 13.0 nm x 13.0 nm x 14.8 nm and contained ~240K atoms.
(TIFF)

S2 Fig Equilibration of structural models. The RMSD of the helix backbone atoms measured against the unequilibrated structure during classical molecular dynamics simulations. Plots for the P_520_ states correspond to model #6 for the wild-type channel and model #2 for the N297D mutant since these systems were chosen for use in ion permeation studies (see S3, S4 Tables).
(TIFF)

S3 Fig Evolution of water distribution and pore opening in the *anti*- and *syn-*cycles. Simulation snapshots of one protomer of the dimeric wild-type C1C2 structure showing the gradual development of a water-filled pore as the channel progressed through the two possible reaction pathways of the photocycle: the *anti*-cycle (A→B→C) and the *syn*-cycle (A→D→E) (see Fig 3). **(A)** The D_470_/C_1_ closed state. Both pathways began with the protein in the initial dark-adapted/ground state with all-*trans*, 15-*anti* retinal and a protonated Schiff base (RSBH^+^). Upon equilibration, water filled the extracellular vestibule while the central gate (CG; residues S102, E129, and N297) and intracellular gate (ICG, residues not shown) remained closed, blocking water passage. In the first reaction pathway (*anti*-cycle), retinal isomerization from all-*trans*, 15-*anti* → 13-*cis*, 15-*anti* formed the first photointermediate state, P_500_. This structure is not shown above since no significant changes to protein conformation or internal water distribution were observed compared to the D_470_/C_1_ closed state. **(B)** The “pre-open” P_390_ intermediate state. Next, deprotonation of the RSBH^+^ by D292 triggered the rearrangement of sidechain interactions that fully opened the CG and weakened some interactions in the ICG. This allowed partial hydration of the permeation pathway from the intracellular side, but a continuous pore did not yet form. **(C)** The high-conducting P_520_/O_1_ open state (S3 Table, model #6). Reprotonation of the RSB and tautomerization of ICG histidines led to moderate shifts in the protein backbone among helices I, II, III, and VII. This triggered complete opening of the ICG and allowed water to completely fill the channel. **(D)** The P_480_/C_2_ closed state. In the second reaction pathway (*syn*-cycle, Fig 3), a double isomerization of the all-*trans*, 15-*anti* retinal from the D_470_/C_1_ closed state to 13-*cis*, 15-*syn* and deprotonation of E129 formed the second light-adapted/desensitized closed state. This event triggered partial opening of the CG that allowed water to hydrate the central region of the channel, while the ICG stayed closed. **(E)** The low-conducting/inactivated second open state, I_530_/O_2_. The third and final isomerization reaction from 13-*cis*, 15-*syn* → all-*trans*, 15-*syn* retinal led to small shifts in pore-framing helices that fully opened both gates, and a continuous water-filled pore was formed. In all parts of the figure, water is shown as transparent gray surfaces. Pink ribbons represent the protein backbone. Retinal and other relevant residues are shown as sticks in cyan. The other protomer, lipids, ions, and nonpolar protons are hidden for clarity.
(TIFF)

S4 Fig Rearrangement of central gating interactions during channel opening in the *anti*-cycle. Schematic representation of hydrogen bonds (orange lines) and weak coulombic interactions (dotted orange lines) among residues of the central gate (black text boxes) and region surrounding the retinal (gray text boxes) for one protomer of the wild-type (WT) C1C2 and N297D mutant channels in each state of the *anti*-cycle. The P_500_ intermediate is omitted since no significant changes to sidechain interactions were observed for either protein compared to the ground state aside from those directly due to retinal isomerization as described in the text. Residues with charged sidechains are marked by a (+) or (-) superscript. RSBH^+^, protonated retinal Schiff base; RSB, deprotonated retinal Schiff base.
(TIFF)

S5 Fig Rearrangement of central gating interactions during channel opening in the *syn*-cycle. Schematic representation of hydrogen bonds (solid orange lines) and weak coulombic interactions (dotted orange lines) among residues of the central gate (black text boxes) and region surrounding the retinal (gray text boxes). Networks are shown for one protomer of the wild-type (WT) C1C2 and N297D mutant channels in each state of the s*yn*-cycle starting from the D_470_/C_1_ ground state. Residues with charged sidechains are marked by a (+) or (-) superscript. RSBH^+^, protonated retinal Schiff base; RSB, deprotonated retinal Schiff base.
(TIFF)

S6 Fig Force profiles from steered molecular dynamics simulations. Steering force (pN) from pulling Na^+^ and Ca^2+^ at constant velocity through the pore of one protomer in each equilibrated open-state structure. Ions were pulled along the *z-*axis of the channel from the extracellular side (*z* ≈ -20 Å) to the intracellular side (*z* ≈ 20 Å). For reference, the central gate and retinal are located around *z* ≈ -5 to 0 Å and the intracellular gate is located around *z* ≈ 10 to 15 Å.
(TIFF)

S7 Fig Convergence of metadynamics simulations. To track convergence, the calculated PMF profile from MWWT-MetaD simulations was output every nanosecond. Convergence was reached when the calculated RMSD between sequential PMFs no longer changed significantly.
(TIFF)

S8 Fig Hydration shells of Na^+^ and Ca^2+^ in the channel. Number of water molecules coordinated by the transiting ion as a function of its CV value (e.g., position along channel axis; see Materials and Methods and Fig 4 in the main text for definition) during MWWT-MetaD simulations. The number of coordinating water molecules was defined as the number of water oxygen atoms within a radial distance of 3.0 Å from the ion for Na^+^ and within 4.0 Å for Ca^2+^ to include calcium’s second hydration shell. Each plot is the combined data for all eight walkers in the system for the full duration of the simulation (100–140 ns per walker). The CG and retinal Schiff base are located between CV ≈ -5 and 0 Å, and the ICG is located around CV ≈ 10 Å.
(TIFF)

S9 Fig Cation trajectories for individual walkers during MWWT-MetaD simulations. The CV value of the biased Na^+^ (left plots) or Ca^2+^ (right plots) along the channel axis is plotted as a function of simulation time for the indicated system. Each trace represents one walker, and there are eight walkers per system. The position of the RSBH^+^ is indicated by a black dashed line at CV = 0 Å for reference. CV values > 15 Å are in the bulk cytosolic solution. Please note that the CV value axis is oriented with negative values at the top (extracellular side) and positive values at the bottom (cytosolic side) to be consistent with the orientation of the protein snapshots in Fig 8 and other figures throughout the text.
(TIFF)

S10 Fig Pore dimensions during ion translocation. Average pore diameter plotted as a function of the CV value determined from MWWT-MetaD simulations during Na^+^ (green) or Ca^2+^ (purple) translocation. Each trace is the ensemble average of all eight walkers of the wild-type C1C2 (solid lines) or N297D (dotted lines) channels in the high-conducting P_520_/O_1_ (left) or low-conducting I_530_/O_2_ (right) open states. Shaded areas are ± SEM, where *N* = 8. Pore diameter was measured using the software program HOLE as described in the data analysis section of the Methods. The gray dotted line at 4.8 Å marks the diameter of hydrated Na^+^ or Ca^2+^ that is defined as twice the peak radial distribution of water oxygen atoms belonging to each ion’s first hydration shell.
(TIFF)

S1 Table Measured reversal potentials of wild-type and mutant channels. Reversal potentials were obtained from current-voltage relationship plots of peak and stationary photocurrents of wild-type C1C2 and mutants measured from *X*. *laevis* oocytes in the indicated 115 mM Na^+^, 115 mM K^+^, or 77 mM Ca^2+^ bath solution (see Methods in the main text for recipes). Each value is an average of 3–18 cells ± SEM. *, *p* < 0.05; **, *p* < 0.01; WT, wild-type; ND, not determined.
(PDF)

S2 Table Ionic permeability ratios. Calculated using Eq (1) or (2) in the methods from the difference of measured reversal potentials recorded from individual cells. Each value is an average of 3–18 cells ± SEM. *, *p* < 0.05; **, *p* < 0.01; WT, wild-type; ND, not determined.
(PDF)

S3 Table Wild-type *anti*-cycle model structures. Model #6 of the P_520_/O_1_ state was used in SMD and metadynamics simulations. *Asterisk, protonated.
(PDF)

S4 Table N297D *anti*-cycle model structures. Model #2 of the P_520_/O_1_ state was used in SMD and metadynamics simulations. *Asterisk, protonated.
(PDF)

S5 Table Wild-type *syn*-cycle model structures. The I_530_/O_2_ state model was used in SMD and metadynamics simulations. *Asterisk, protonated.
(PDF)

S6 Table N297D *syn*-cycle model structures. The I_530_/O_2_ state model was used in SMD and metadynamics simulations. *Asterisk, protonated.
(PDF)

S1 Text Supporting information: Methods. Describes methods related to molecular modeling of photocycle states D_470_/C_1_, P_500_, P_390_, and P_480_/C_2_ and retinal isomerization for the dimeric wild-type C1C2 and N297D mutant channels, steered molecular dynamics, and a discussion on our computational approach for calculating the PMF.
(PDF)

S2 Text Supporting information: Results and discussion. Presents modeling results for photocycle states D_470_/C_1_, P_500_, P_390_, and P_480_/C_2_ for the dimeric wild-type C1C2 and N297D mutant channels.
(PDF)
