# Supplementary figures and images for: Metadynamics simulations reveal mechanisms of Na+ and Ca2+ transport in two open states of the channelrhodopsin chimera, C1C2
Source: PLoS One. 2024 Sep 6;19(9):e0309553. doi: 10.1371/journal.pone.0309553 (PMC11379304; doi:10.1371/journal.pone.0309553)

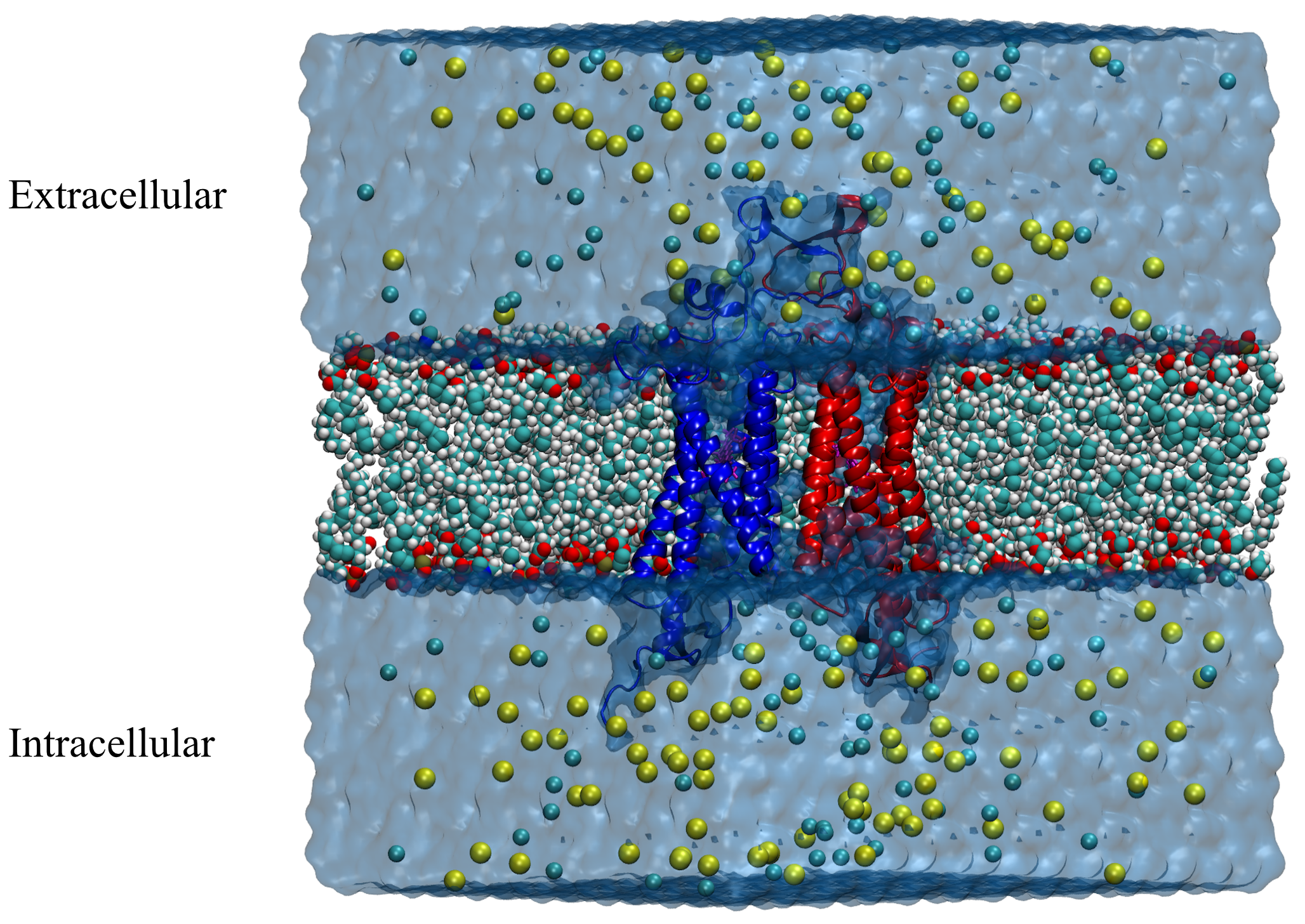

Supplement: S1 Fig — Snapshot of the unequilibrated system used in molecular dynamics simulations containing the wild-type C1C2 dimer embedded in a DOPC lipid bilayer with explicit water and 150 mM NaCl. Protomer A is in blue and protomer B is in red. Lipids are represented as space-filling molecules with foreground lipids hidden for clarity. Water molecules are rendered as blue-gray transparent surfaces on either side of the membrane. Yellow spheres are Na+. Cyan spheres are Cl-. The simulation box measured 13.0 nm x 13.0 nm x 14.8 nm and contained ~240K atoms. (TIFF) [file pone.0309553.s001.tiff]

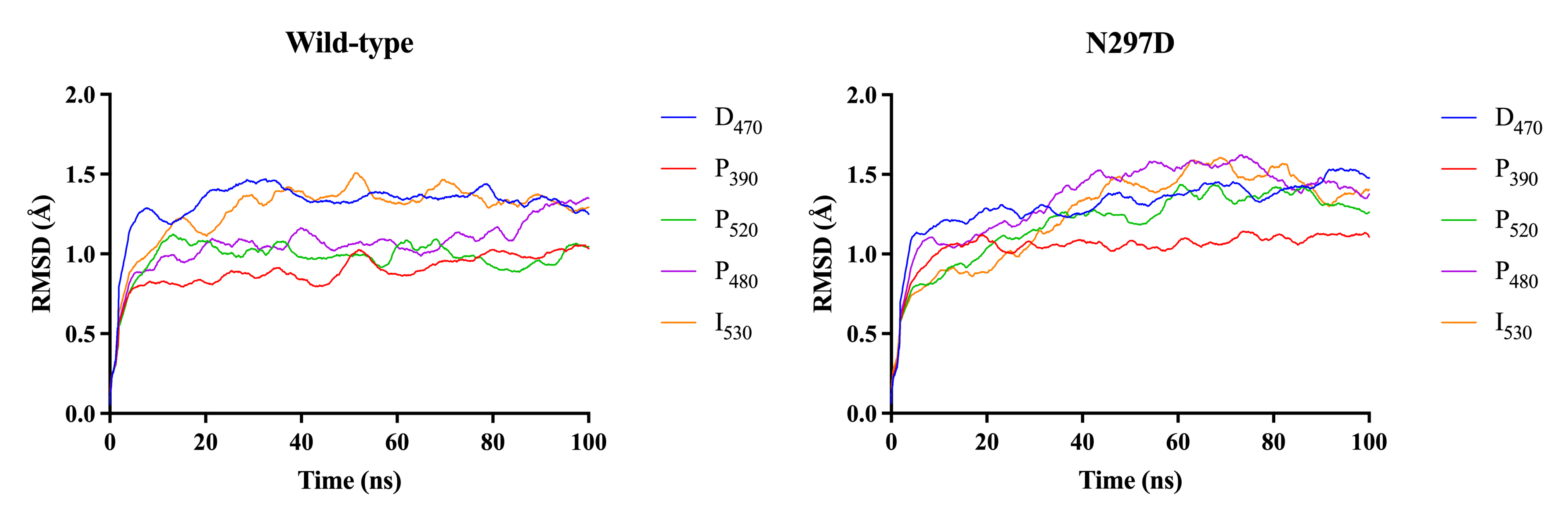

Supplement: S2 Fig — The RMSD of the helix backbone atoms measured against the unequilibrated structure during classical molecular dynamics simulations. Plots for the P520 states correspond to model #6 for the wild-type channel and model #2 for the N297D mutant since these systems were chosen for use in ion permeation studies (see S3, S4 Tables). (TIFF) [file pone.0309553.s002.tiff]

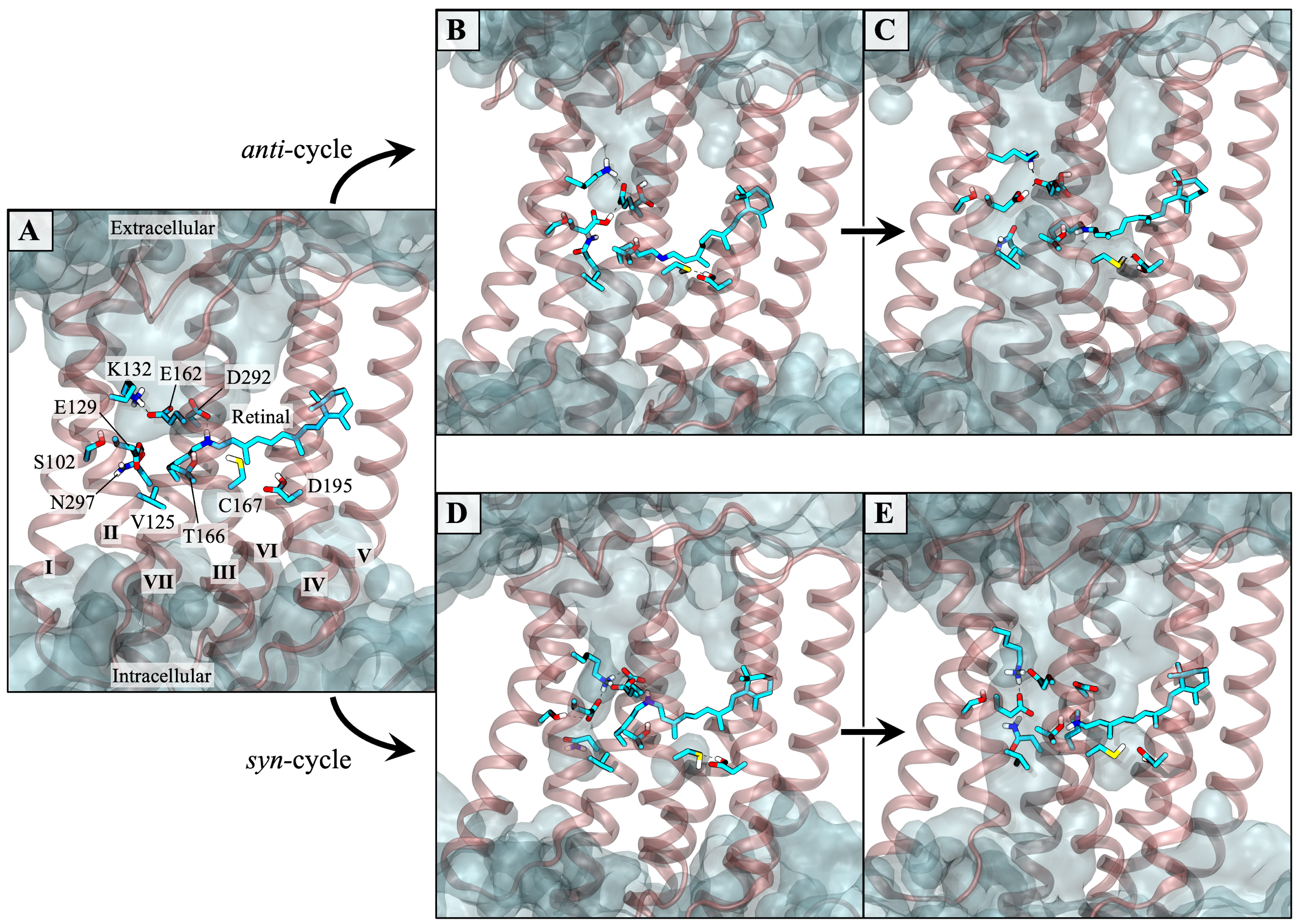

Supplement: S3 Fig — Simulation snapshots of one protomer of the dimeric wild-type C1C2 structure showing the gradual development of a water-filled pore as the channel progressed through the two possible reaction pathways of the photocycle: the anti-cycle (A→B→C) and the syn-cycle (A→D→E) (see Fig 3). (A) The D470/C1 closed state. Both pathways began with the protein in the initial dark-adapted/ground state with all-trans, 15-anti retinal and a protonated Schiff base (RSBH+). Upon equilibration, water filled the extracellular vestibule while the central gate (CG; residues S102, E129, and N297) and intracellular gate (ICG, residues not shown) remained closed, blocking water passage. In the first reaction pathway (anti-cycle), retinal isomerization from all-trans, 15-anti → 13-cis, 15-anti formed the first photointermediate state, P500. This structure is not shown above since no significant changes to protein conformation or internal water distribution were observed compared to the D470/C1 closed state. (B) The “pre-open” P390 intermediate state. Next, deprotonation of the RSBH+ by D292 triggered the rearrangement of sidechain interactions that fully opened the CG and weakened some interactions in the ICG. This allowed partial hydration of the permeation pathway from the intracellular side, but a continuous pore did not yet form. (C) The high-conducting P520/O1 open state (S3 Table, model #6). Reprotonation of the RSB and tautomerization of ICG histidines led to moderate shifts in the protein backbone among helices I, II, III, and VII. This triggered complete opening of the ICG and allowed water to completely fill the channel. (D) The P480/C2 closed state. In the second reaction pathway (syn-cycle, Fig 3), a double isomerization of the all-trans, 15-anti retinal from the D470/C1 closed state to 13-cis, 15-syn and deprotonation of E129 formed the second light-adapted/desensitized closed state. This event triggered partial opening of the CG that allowed water to hydrate the central region o [file pone.0309553.s003.tiff]

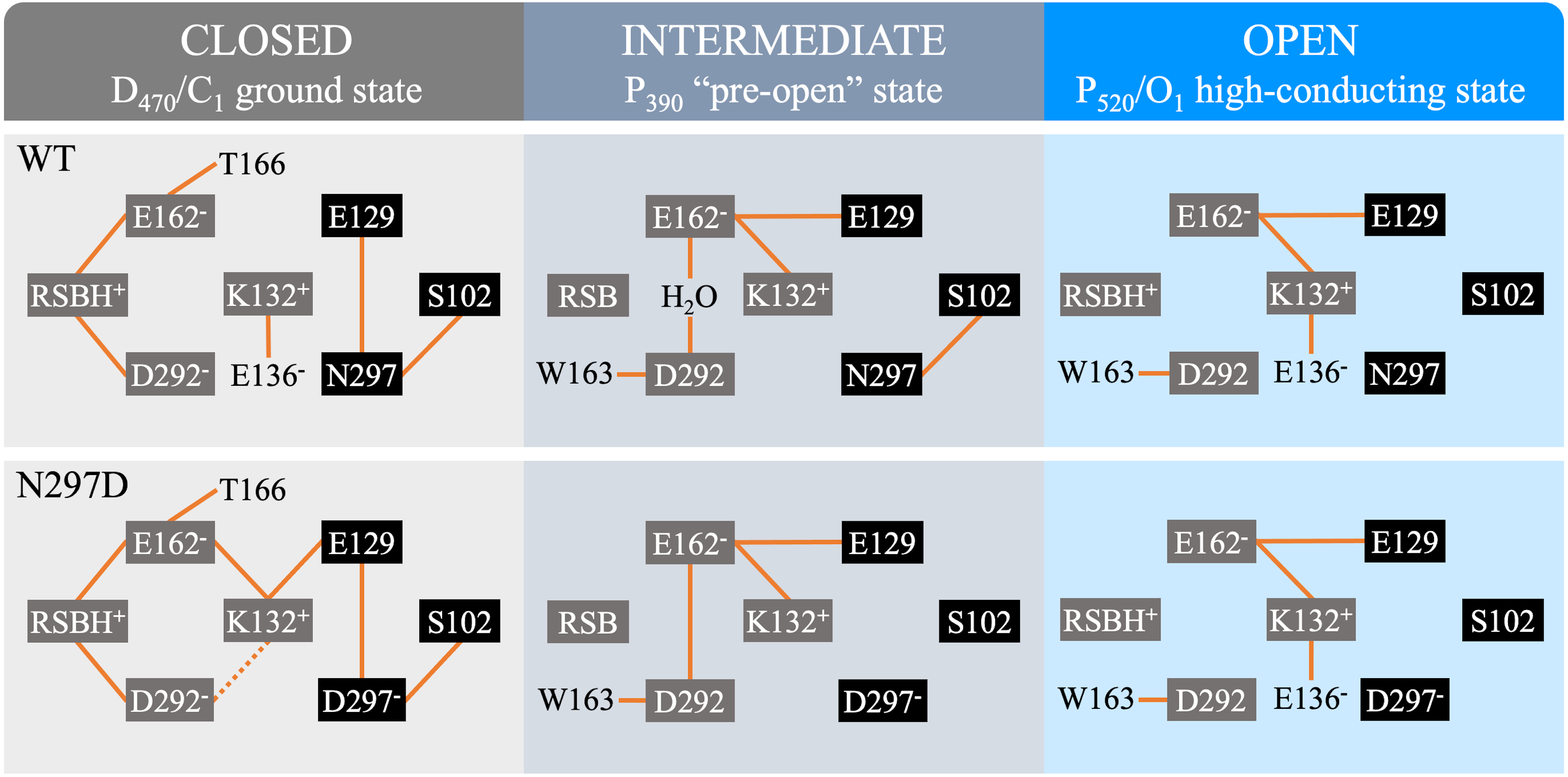

Supplement: S4 Fig — Schematic representation of hydrogen bonds (orange lines) and weak coulombic interactions (dotted orange lines) among residues of the central gate (black text boxes) and region surrounding the retinal (gray text boxes) for one protomer of the wild-type (WT) C1C2 and N297D mutant channels in each state of the anti-cycle. The P500 intermediate is omitted since no significant changes to sidechain interactions were observed for either protein compared to the ground state aside from those directly due to retinal isomerization as described in the text. Residues with charged sidechains are marked by a (+) or (-) superscript. RSBH+, protonated retinal Schiff base; RSB, deprotonated retinal Schiff base. (TIFF) [file pone.0309553.s004.tiff]

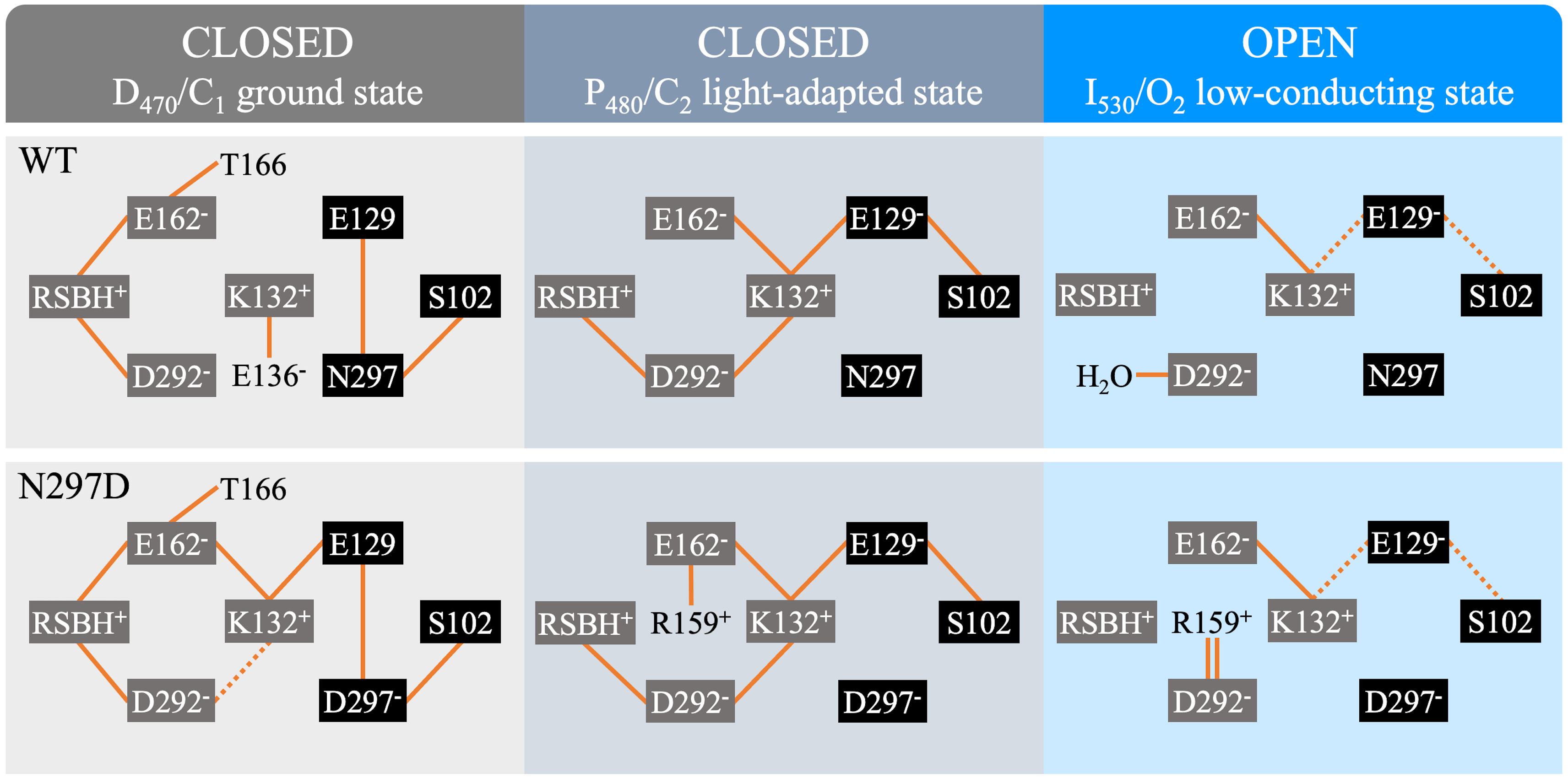

Supplement: S5 Fig — Schematic representation of hydrogen bonds (solid orange lines) and weak coulombic interactions (dotted orange lines) among residues of the central gate (black text boxes) and region surrounding the retinal (gray text boxes). Networks are shown for one protomer of the wild-type (WT) C1C2 and N297D mutant channels in each state of the syn-cycle starting from the D470/C1 ground state. Residues with charged sidechains are marked by a (+) or (-) superscript. RSBH+, protonated retinal Schiff base; RSB, deprotonated retinal Schiff base. (TIFF) [file pone.0309553.s005.tiff]

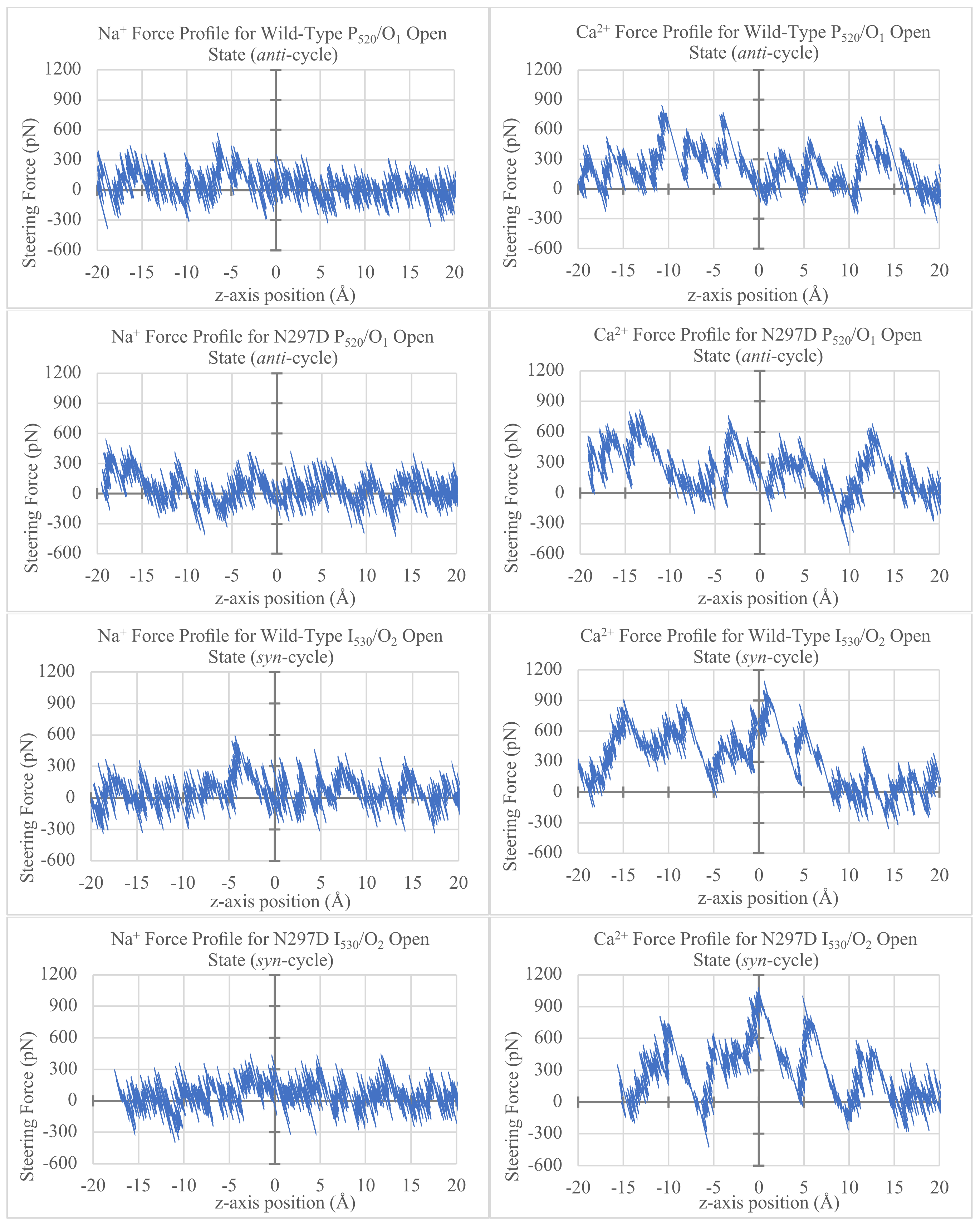

Supplement: S6 Fig — Steering force (pN) from pulling Na+ and Ca2+ at constant velocity through the pore of one protomer in each equilibrated open-state structure. Ions were pulled along the z-axis of the channel from the extracellular side (z ≈ -20 Å) to the intracellular side (z ≈ 20 Å). For reference, the central gate and retinal are located around z ≈ -5 to 0 Å and the intracellular gate is located around z ≈ 10 to 15 Å. (TIFF) [file pone.0309553.s006.tiff]

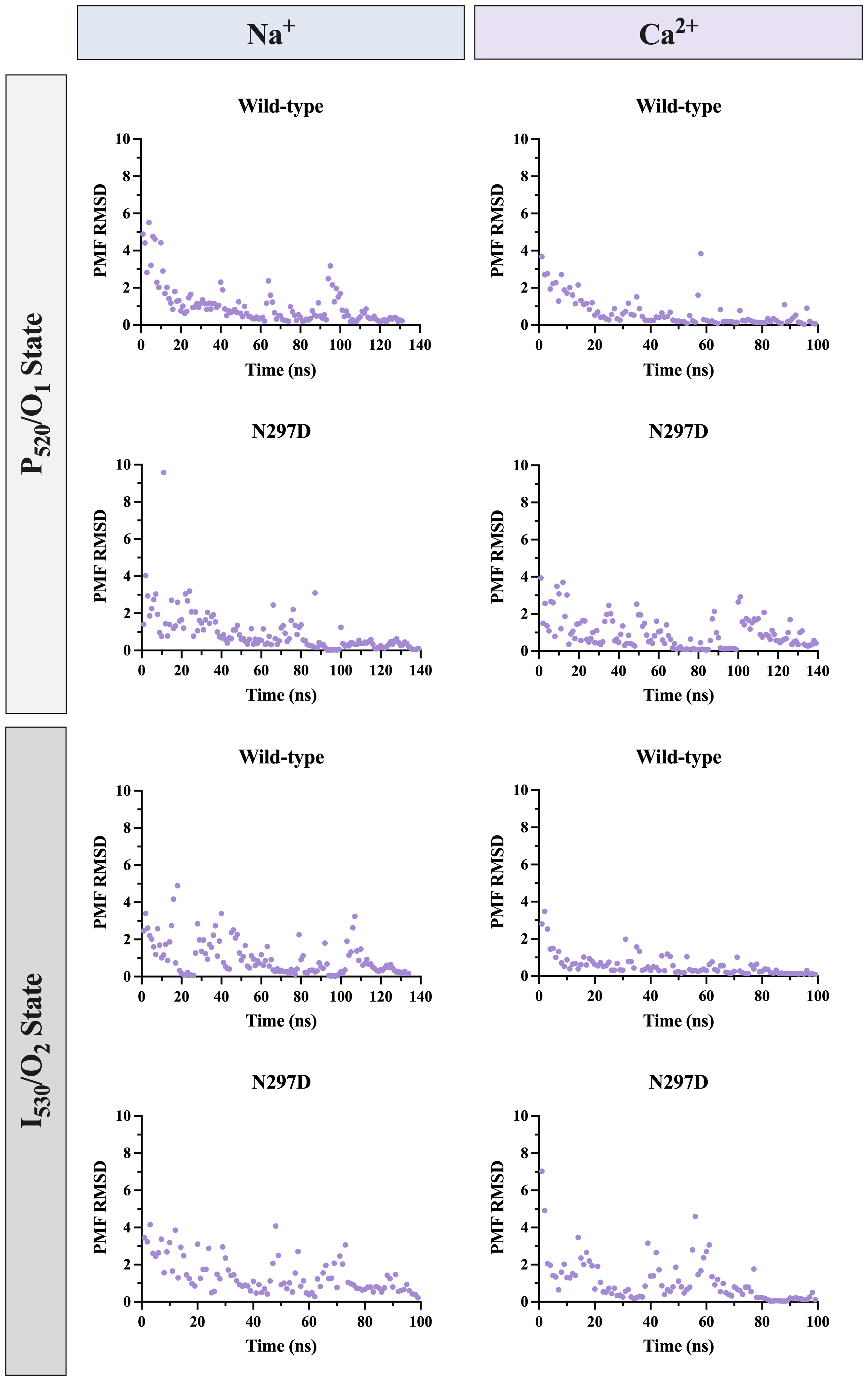

Supplement: S7 Fig — To track convergence, the calculated PMF profile from MWWT-MetaD simulations was output every nanosecond. Convergence was reached when the calculated RMSD between sequential PMFs no longer changed significantly. (TIFF) [file pone.0309553.s007.tiff]

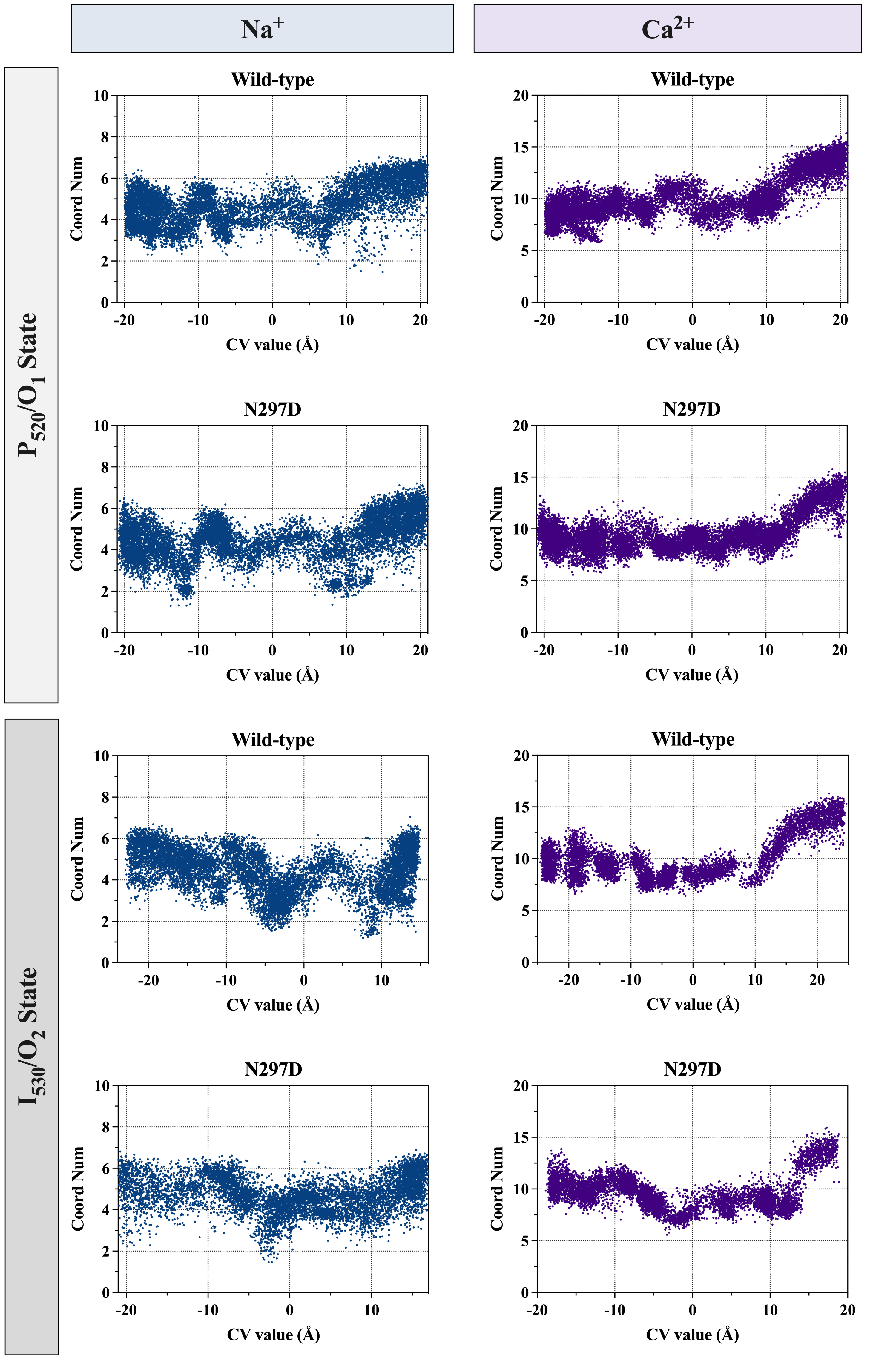

Supplement: S8 Fig — Number of water molecules coordinated by the transiting ion as a function of its CV value (e.g., position along channel axis; see Materials and Methods and Fig 4 in the main text for definition) during MWWT-MetaD simulations. The number of coordinating water molecules was defined as the number of water oxygen atoms within a radial distance of 3.0 Å from the ion for Na+ and within 4.0 Å for Ca2+ to include calcium’s second hydration shell. Each plot is the combined data for all eight walkers in the system for the full duration of the simulation (100–140 ns per walker). The CG and retinal Schiff base are located between CV ≈ -5 and 0 Å, and the ICG is located around CV ≈ 10 Å. (TIFF) [file pone.0309553.s008.tiff]

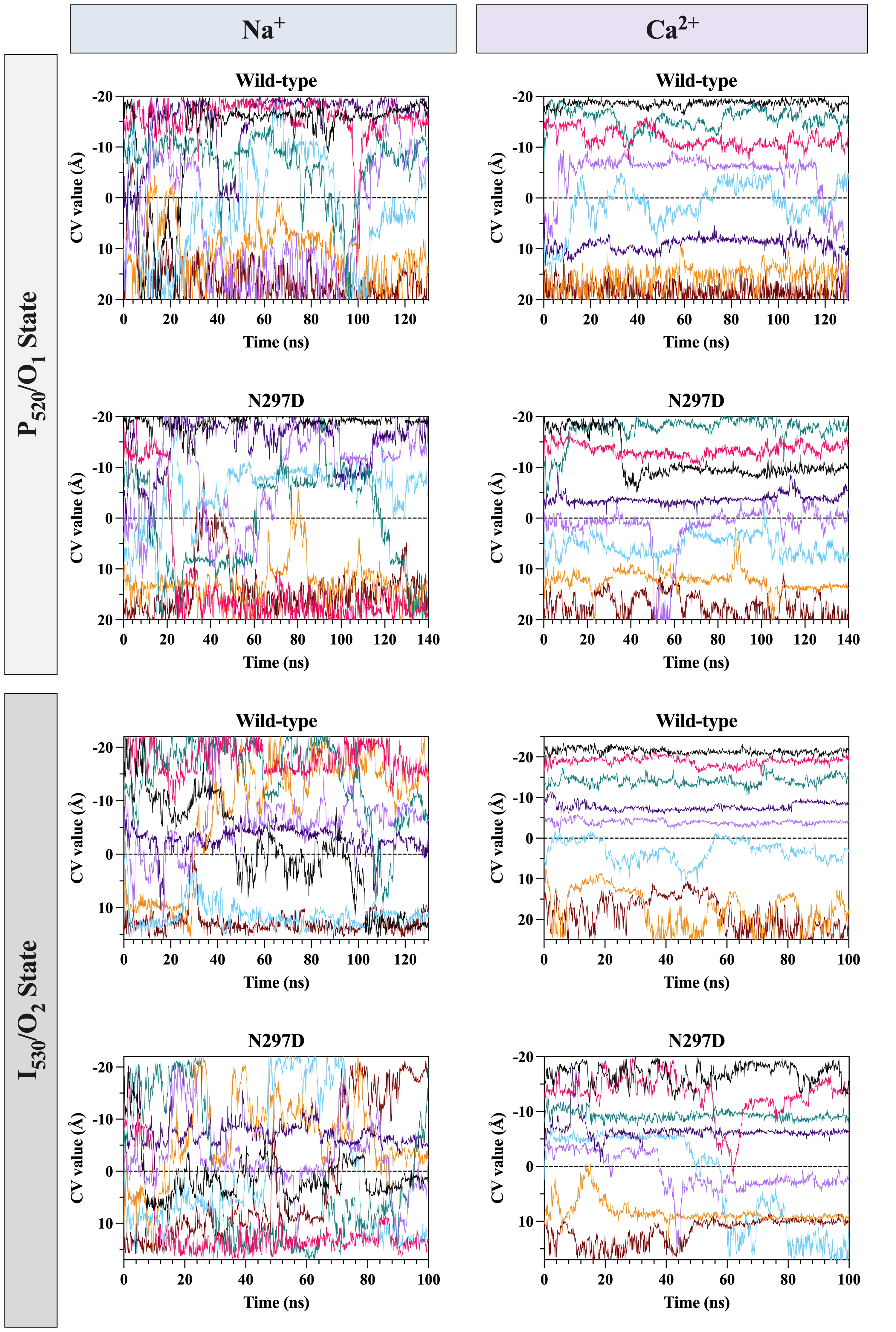

Supplement: S9 Fig — The CV value of the biased Na+ (left plots) or Ca2+ (right plots) along the channel axis is plotted as a function of simulation time for the indicated system. Each trace represents one walker, and there are eight walkers per system. The position of the RSBH+ is indicated by a black dashed line at CV = 0 Å for reference. CV values > 15 Å are in the bulk cytosolic solution. Please note that the CV value axis is oriented with negative values at the top (extracellular side) and positive values at the bottom (cytosolic side) to be consistent with the orientation of the protein snapshots in Fig 8 and other figures throughout the text. (TIFF) [file pone.0309553.s009.tiff]

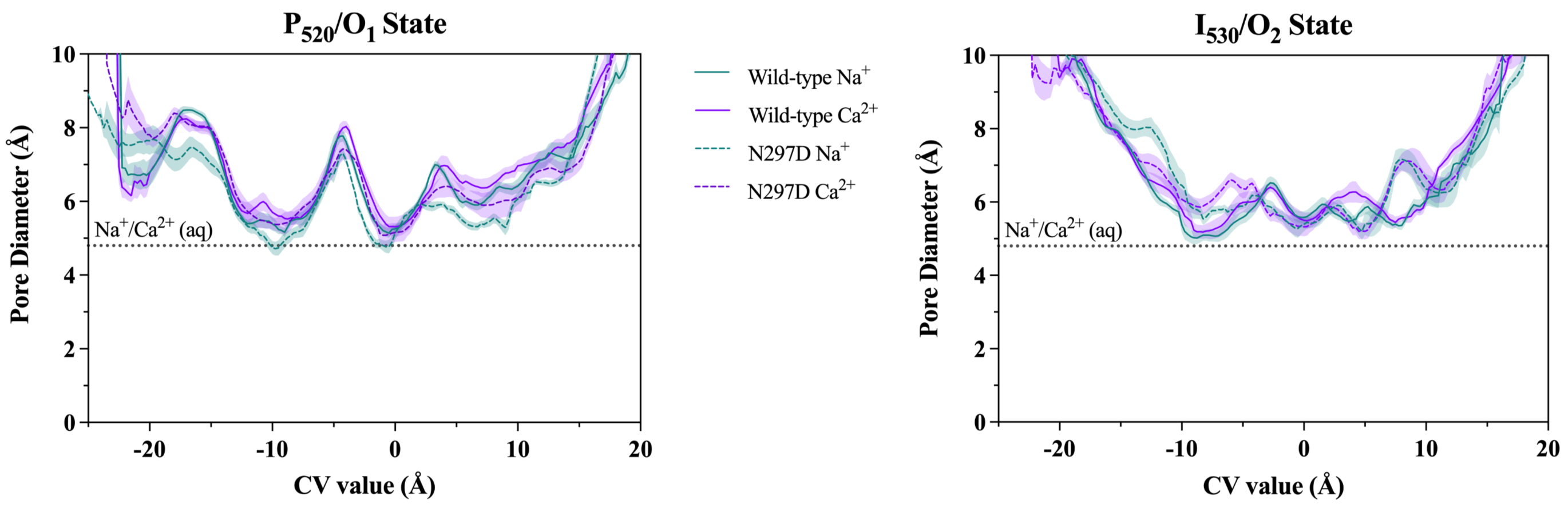

Supplement: S10 Fig — Average pore diameter plotted as a function of the CV value determined from MWWT-MetaD simulations during Na+ (green) or Ca2+ (purple) translocation. Each trace is the ensemble average of all eight walkers of the wild-type C1C2 (solid lines) or N297D (dotted lines) channels in the high-conducting P520/O1 (left) or low-conducting I530/O2 (right) open states. Shaded areas are ± SEM, where N = 8. Pore diameter was measured using the software program HOLE as described in the data analysis section of the Methods. The gray dotted line at 4.8 Å marks the diameter of hydrated Na+ or Ca2+ that is defined as twice the peak radial distribution of water oxygen atoms belonging to each ion’s first hydration shell. (TIFF) [file pone.0309553.s010.tiff]
