# Supplementary material for: Metadynamics simulations reveal mechanisms of Na+ and Ca2+ transport in two open states of the channelrhodopsin chimera, C1C2
Source: PLoS One. 2024 Sep 6;19(9):e0309553. doi: 10.1371/journal.pone.0309553 (PMC11379304; doi:10.1371/journal.pone.0309553)
Supplement: S1 Table — Reversal potentials were obtained from current-voltage relationship plots of peak and stationary photocurrents of wild-type C1C2 and mutants measured from X. laevis oocytes in the indicated 115 mM Na+, 115 mM K+, or 77 mM Ca2+ bath solution (see Methods in the main text for recipes). Each value is an average of 3–18 cells ± SEM. *, p < 0.05; **, p < 0.01; WT, wild-type; ND, not determined. (PDF) [file pone.0309553.s011.pdf]

S1 Table. Measured reversal potentials of wild-type and mutant channels. Reversal potentials were obtained from current-voltage relationship plots of peak and stationary photocurrents of wild-type C1C2 and mutants measured from *X. laevis* oocytes in the indicated 115 mM Na^+, 115 mM K^+, or 77 mM Ca^{2+} bath solution (see methods in the main text for recipes). Each value is an average of 3-18 cells ± SEM. *, $p < 0.05$; **, $p < 0.01$; WT, wild-type; ND, not determined.

	Peak Current E_{rev} (mV)				Stationary Current E_{rev} (mV)			
**Bath solution**	**WT**	**V125L**	**N297D**	**N297V**	**WT**	**V125L**	**N297D**	**N297V**
Na^+, pH 7	-3.5 ± 0.9	ND	-2.9 ± 0.6	-5.1 ± 0.7	-4.1 ± 0.7	-9.8 ± 1.1**	-2.3 ± 1.0	-6.8 ± 1.2
K^+, pH 7	-8.2 ± 1.2	ND	-7.2 ± 0.6	-9.5 ± 0.4	-7.4 ± 1.1	-11.0 ± 1.1*	-5.4 ± 0.8	-10.9 ± 0.6*
Na^+, pH 9	-23.9 ± 0.8	ND	-16.5 ± 1.8**	-32.6 ± 0.2**	-22.5 ± 1.2	-28.2 ± 1.1**	-19.8 ± 0.8	-37.8 ± 1.4**
K^+, pH 9	-36.2 ± 0.7	ND	-26.0 ± 0.8**	-45.7 ± 0.6**	-31.8 ± 1.4	-29.7 ± 1.2	-24.7 ± 1.8*	-47.2 ± 1.4**
Na^+, pH 7 BAPTA-AM	-13.4 ± 0.8	ND	-6.2 ± 0.7**	-9.6 ± 1.1	-13.2 ± 1.0	-13.6 ± 1.0	-8.0 ± 0.8**	-9.7 ± 1.3
Ca^{2+}, pH 7 BAPTA-AM	-27.8 ± 0.4	ND	-11.3 ± 0.8**	-25.0 ± 0.9*	-26.6 ± 0.3	-29.3 ± 2.4	-16.8 ± 0.7**	-22.5 ± 1.0**
