# Supplementary material for: Metadynamics simulations reveal mechanisms of Na+ and Ca2+ transport in two open states of the channelrhodopsin chimera, C1C2
Source: PLoS One. 2024 Sep 6;19(9):e0309553. doi: 10.1371/journal.pone.0309553 (PMC11379304; doi:10.1371/journal.pone.0309553)
Supplement: S2 Table — Calculated using Eq (1) or (2) in the methods from the difference of measured reversal potentials recorded from individual cells. Each value is an average of 3–18 cells ± SEM. *, p < 0.05; **, p < 0.01; WT, wild-type; ND, not determined. (PDF) [file pone.0309553.s012.pdf]

S2 Table. Ionic permeability ratios. Calculated using Eq (1) or (2) in the methods from the difference of measured reversal potentials recorded from individual cells. Each value is an average of 3-18 cells ± SEM. *, $p < 0.05$; **, $p < 0.01$; WT, wild-type; ND, not determined.

P_X / P_{Na}	Peak Current				Stationary Current			
X **=**	**WT**	**V125L**	**N297D**	**N297V**	**WT**	**V125L**	**N297D**	**N297V**
H^+(x10^7)	0.24 ± 0.01	ND	0.20 ± 0.02	0.30 ± 0.01**	0.21 ± 0.01	0.26 ± 0.02*	0.20 ± 0.01	0.36 ± 0.04*
K^+	0.83 ± 0.03	ND	0.85 ± 0.03	0.84 ± 0.04	0.88 ± 0.03	0.95 ± 0.03	0.89 ± 0.07	0.85 ± 0.07
Ca^{2+}	0.29 ± 0.01	ND	0.51 ± 0.02**	0.28 ± 0.01	0.30 ± 0.01	0.26 ± 0.03	0.40 ± 0.01**	0.32 ± 0.02
