# Supplementary material for: Metadynamics simulations reveal mechanisms of Na+ and Ca2+ transport in two open states of the channelrhodopsin chimera, C1C2
Source: PLoS One. 2024 Sep 6;19(9):e0309553. doi: 10.1371/journal.pone.0309553 (PMC11379304; doi:10.1371/journal.pone.0309553)
Supplement: S3 Table — Model #6 of the P520/O1 state was used in SMD and metadynamics simulations. *Asterisk, protonated. (PDF) [file pone.0309553.s013.pdf]

S3 Table. Wild-type *anti*-cycle model structures. Model #6 of the P_{520}/O_1 state was used in SMD and metadynamics simulations. *Asterisk, protonated.

	**Model State**								
**Residue:**	**D_{470}/C_1**	**P_{500}**	**P_{390}**	**P_{520}/O_1**					
**Retinal**	13-*trans*, 15-*anti* *	13-*cis*, 15-*anti* *	13-*cis*, 15-*anti*	13-*cis*, 15-*anti* *					
**Model #**				1	2	3	4	5	6
**E129**	*	*	*	*	*	*	*	*	*
**D195**	*	*	*		*		*	*	*
**D292**			*	*	*	*	*	*	*
**H173**	δ-H	δ-H	δ-H	δ-H	δ-H	δ-H	δ-H	ϵ-H[a]	ϵ-H
**H304**	δ-H	δ-H	δ-H	δ-H	*	*	ϵ-H	ϵ-H	ϵ-H
**# H_2O:**									
**Protomer A**	48 ± 0.2	41 ± 0.3	36 ± 0.2	34 ± 0.1	56 ± 0.3	49 ± 0.3	65 ± 0.3	52 ± 0.4	75 ± 0.3
**Protomer B**	47 ± 0.2	43 ± 0.2	42 ± 0.2	39 ± 0.1	67 ± 0.2	45 ± 0.2	74 ± 0.4	72 ± 0.3	75 ± 0.3
***r*(HII-HVII) [Å]:**									
**Protomer A**	5.79 ± 0.02	5.85 ± 0.03	5.71 ± 0.02	5.65 ± 0.02	7.05 ± 0.05	5.64 ± 0.03	6.64 ± 0.03	6.88 ± 0.04	9.41 ± 0.04
**Protomer B**	6.36 ± 0.02	6.08 ± 0.03	6.68 ± 0.02	6.66 ± 0.02	7.86 ± 0.03	5.88 ± 0.03	8.28 ± 0.03	8.54 ± 0.03	8.10 ± 0.03

[a] δ-H on protomer A; ϵ-H on protomer B.
