# Supplementary material for: Metadynamics simulations reveal mechanisms of Na+ and Ca2+ transport in two open states of the channelrhodopsin chimera, C1C2
Source: PLoS One. 2024 Sep 6;19(9):e0309553. doi: 10.1371/journal.pone.0309553 (PMC11379304; doi:10.1371/journal.pone.0309553)
Supplement: S4 Table — Model #2 of the P520/O1 state was used in SMD and metadynamics simulations. *Asterisk, protonated. (PDF) [file pone.0309553.s014.pdf]

S4 Table. N297D *anti*-cycle model structures. Model #2 of the P_{520}/O_1 state was used in SMD and metadynamics simulations. *Asterisk, protonated.

	Model State				
**Residue:**	**D_{470}/C_1**	**P_{500}**	**P_{390}**	**P_{520}/O_1**	
**Retinal**	13-*trans*, 15-*anti* *	13-*cis*, 15-*anti* *	13-*cis*, 15-*anti*	13-*cis*, 15-*anti* *	
**Model #**				1	2
**E129**	*	*	*	*	*
**D195**	*	*	*		*
**D292**			*	*	*
**H173**	δ-H	δ-H	δ-H	δ-H	ϵ-H
**H304**	δ-H	δ-H	δ-H	δ-H	ϵ-H
**# H_2O:**					
**Protomer A**	47 ± 0.2	47 ± 0.2	57 ± 0.3	63 ± 0.2	68 ± 0.3
**Protomer B**	40 ± 0.2	38 ± 0.3	46 ± 0.3	48 ± 0.2	84 ± 0.4
***r*(HII-HVII) [Å]:**					
**Protomer A**	7.06 ± 0.02	6.90 ± 0.01	7.45 ± 0.02	7.79 ± 0.02	8.33 ± 0.02
**Protomer B**	6.47 ± 0.03	6.29 ± 0.01	6.49 ± 0.03	6.30 ± 0.02	10.06 ± 0.04
