# Supplementary material for: Metadynamics simulations reveal mechanisms of Na+ and Ca2+ transport in two open states of the channelrhodopsin chimera, C1C2
Source: PLoS One. 2024 Sep 6;19(9):e0309553. doi: 10.1371/journal.pone.0309553 (PMC11379304; doi:10.1371/journal.pone.0309553)
Supplement: S5 Table — The I530/O2 state model was used in SMD and metadynamics simulations. *Asterisk, protonated. (PDF) [file pone.0309553.s015.pdf]

S5 Table. Wild-type *syn*-cycle model structures. The I_{530}/O_2 state model was used in SMD and metadynamics simulations. *Asterisk, protonated.

	Model State		
**Residue:**	**D_{470}/C_1**	**P_{480}/C_2**	**I_{530}/O_2**
**Retinal**	13-*trans*, 15-*anti* *	13-*cis*, 15-*syn* *	13-*trans*, 15-*syn* *
**E129**	*		
**D195**	*	*	*
**D292**			
**H173**	δ-H	δ-H	ϵ-H
**H304**	δ-H	δ-H	δ-H
**# H_2O:**			
**Protomer A**	48 ± 0.2	54 ± 0.3	82 ± 0.3
**Protomer B**	47 ± 0.2	55 ± 0.3	90 ± 0.4
***r*(HII-HVII) [Å]:**			
**Protomer A**	5.79 ± 0.02	8.10 ± 0.03	8.06 ± 0.03
**Protomer B**	6.36 ± 0.02	7.04 ± 0.04	8.50 ± 0.04
