# Supplementary material for: Metadynamics simulations reveal mechanisms of Na+ and Ca2+ transport in two open states of the channelrhodopsin chimera, C1C2
Source: PLoS One. 2024 Sep 6;19(9):e0309553. doi: 10.1371/journal.pone.0309553 (PMC11379304; doi:10.1371/journal.pone.0309553)
Supplement: S6 Table — The I530/O2 state model was used in SMD and metadynamics simulations. *Asterisk, protonated. (PDF) [file pone.0309553.s016.pdf]

S6 Table. N297D *syn*-cycle model structures. The I_{530}/O_2 state model was used in SMD and metadynamics simulations. *Asterisk, protonated.

	**Model State**		
**Residue:**	**D_{470}/C_1**	**P_{480}/C_2**	**I_{530}/O_2**
**Retinal**	13-*trans*, 15-*anti*<br>*	13-*cis*, 15-*syn*<br>*	13-*trans*, 15-*syn*<br>*
**E129**	*		
**D195**	*	*	*
**D292**			
**H173**	δ-H	δ-H	ϵ-H
**H304**	δ-H	δ-H	ϵ-H
**# H_2O:**			
**Protomer A**	47 ± 0.2	56 ± 0.3	87 ± 0.3
**Protomer B**	40 ± 0.2	54 ± 0.3	95 ± 0.5
***r*(HII-HVII) [Å]:**			
**Protomer A**	7.06 ± 0.02	8.46 ± 0.03	10.01 ± 0.04
**Protomer B**	6.47 ± 0.03	8.44 ± 0.04	8.70 ± 0.04
