# Supplementary material for: Metadynamics simulations reveal mechanisms of Na+ and Ca2+ transport in two open states of the channelrhodopsin chimera, C1C2
Source: PLoS One. 2024 Sep 6;19(9):e0309553. doi: 10.1371/journal.pone.0309553 (PMC11379304; doi:10.1371/journal.pone.0309553)
Supplement: S1 Text — Describes methods related to molecular modeling of photocycle states D470/C1, P500, P390, and P480/C2 and retinal isomerization for the dimeric wild-type C1C2 and N297D mutant channels, steered molecular dynamics, and a discussion on our computational approach for calculating the PMF. (PDF) [file pone.0309553.s017.pdf]

Supporting information: Methods

The following describes the methods related to molecular modeling of photocycle states D_{470}/C_1, P_{500}, P_{390}, and P_{480}/C_2 for the dimeric wild-type C1C2 and N297D mutant channels and associated retinal isomerizations, steered molecular dynamics, and a discussion on our computational approach for calculating the PMF. The relevant evidence from the literature that informed our choice of retinal conformation and protonation states of titratable residues in each photocycle model is provided alongside the modeling details for each state except for the P_{520}/O_1 open state, whose rationale is given in a dedicated section below.

Modeling of the dark-adapted D_{470}/C_1 closed state

The dark adapted D_{470}/C_1 ground state was modeled with protonated all-*trans*, 15-*anti* retinal and with residues E129 and D195 protonated based on results from our previous modeling study of the C1C2 monomer in the closed state, where alternative configurations for the protonation states of titratable residues in this state were thoroughly explored [1]. This configuration is also in agreement with published spectroscopic, NMR, and X-ray data of wild-type C1C2 and ChR2 in the dark-adapted closed state [2–6]. The fully equilibrated D_{470}/C_1 state structure was used as a starting point to build the structures for the next state in the sequence of each photocycle, which is the P_{500} state for the *anti*-cycle and the P_{480}/C_2 state for the *syn*-cycle.

Modeling of *anti*-cycle intermediate states

Photoactivation of C1C2 from the dark adapted D_{470}/C_1 ground state by illumination with 470 nm blue light triggers photoisomerization of the retinal from all-*trans*, 15-*anti* → 13-*cis*, 15-*anti* that forms the P_{500} intermediate and initiates the process of channel opening in the *anti*-cycle [5,7,8]. This transition was simulated using the following procedure.

all-*trans*, 15-*anti* → 13-*cis*, 15-*anti* retinal isomerization: The fully equilibrated D_{470}/C_1 state system including protein dimer, membrane lipids, water, and ions was used to approximate the all-*trans*, 15-*anti* → 13-*cis*, 15-*anti* isomerization of retinal in a stepwise manner. The same procedure was carried out in both protomers. The dihedral angle formed by atoms C12-C13=C14-C15 (Fig 1) of the retinal polyene chain was rotated in 20º steps from 180º to 0º while the C15=N dihedral angle was restrained at 180º. Between each rotation step, dihedral restraints were placed on the remaining atoms of the retinal polyene chain to maintain planarity while the rest of the system was allowed to relax for 1 ns. Once isomerization was complete, the system was relaxed for an additional 9 ns. The resulting structure of retinal in the 13-*cis*, 15-*anti* conformation was used in all subsequent intermediates of the *anti*-cycle (P_{500}, P_{390}, and P_{520}/O_1; see Fig 3).

While this approximation of retinal isomerization neglects the excited state and single-bond distortions in the polyene chain, this method has been used by other groups in the past with excellent results comparable to those obtained by more rigorous QM/MM calculations [7,9]. Moreover, this approach is sufficient for our purposes since we are primarily interested in events that occur after retinal isomerization in this work.

P_{500} state: The last frame from the all-*trans*, 15-*anti* → 13-*cis*, 15-*anti* isomerization was used to model the P_{500} state. Time-resolved FTIR and transient absorption spectroscopy experiments show the D_{470}/C_1 → P_{500} state transition occurs within < 450 fs of photon absorption, with little to no movement of the protein backbone, and with no proton transfer between residues

[5,6,9]. Therefore, protonation states of ionizable residues in the P_{500} state model were kept the same as in the D_{470}/C_1 state model.

P_{390} state: The last trajectory frame of the equilibrated P_{500} system was used to build the initial structure of the P_{390} "pre-open" intermediate. Residues E129, D195, and D292 were modeled as protonated while the retinal Schiff base was deprotonated to match experimental data [7–9]. All other ionizable residues were modeled in their standard protonation states at physiological pH. Optimized force field parameters for deprotonated retinal were obtained from Zhu et al. [10].

Rationale for choice of protonation states in the P_{520}/O_1 open state models

Multiple time-resolved FTIR and Raman spectroscopy measurements of ChR2 and C1C2 under single-turnover conditions have demonstrated that the $P_{390} \rightarrow P_{520}/O_1$ transition occurs with reprotonation of the RSB from an internal proton donor, and that D292 and E129 remain protonated in the P_{520}/O_1 state [7,9,11,12]. However, the identity of the residue that serves as the RSB's proton donor is still hotly debated. Some studies suggest that in ChR2, deprotonation of DC gate residue D156 (D195 in C1C2) occurs concurrent with reprotonation of the Schiff base upon P_{390} depletion, and therefore D156 was assigned as the putative internal proton donor to the Schiff base [7,11].

Other reports argue that the timing of D156 deprotonation occurs too early relative to retinal reprotonation, therefore the Schiff base must receive a proton from some other source [7,13]. In contrast, FTIR analysis in C1C2 showed that spectral bands of D195 are unaffected by H_2O/D_2O exchange, indicating that D195 is likely not part of the proton transfer pathway in C1C2 and may not become deprotonated at all during the opening process [14]. Results of pK_a analyses reported in our previous molecular modeling study of the C1C2 open state monomer support this scenario, since the pK_a values of the DC gate residues were among those least affected by changes in pore structure or hydration upon channel opening [15].

An alternative source for the RSB proton could be either H173 or H304 that form part of the intracellular gate. This behavior would make channel opening in C1C2 more similar to that of bacteriorhodopsin, where the inner gating residue D96 (H173 in C1C2) serves as the primary proton donor to the Schiff base [16]. Initially, the conserved H173 residue was ruled out as a potential RSB proton donor when a study in ChR1 found that the H173D mutation completely abolished the light-induced current, whereas the H173R and H173Y mutants retained channel function [17]. A similar effect was seen in ChR2, where the analogous mutation H134R reduced proton conductance but also generated larger photocurrents by increasing the channel's open state lifetime [18–20].

However, it was found that in C1C2, the H173R mutation does not increase photocurrent amplitude, severely reduces proton and cation selectivity, and interferes with channel closing that causes a leak current in the dark [21]. This data suggests that H173 is important for proton conductance in C1C2, and that transient protonation of this residue may help to open the inner gate, whether or not it serves as the RSB proton donor. In line with this reasoning, we speculate that the adjacent solvent-accessible histidine, H304, could also perform this function as part of the inner gate. However, the functional role of H304 has not been deeply explored in the literature.

Therefore, since the protonation states of residues D292, E129, and the $RSBH^+$ are reasonably well established for the P_{520}/O_1 open state, all six models of this state for wild-type C1C2 channel were configured with protonated D292, E129, and $RSBH^+$, while residues whose protonation states are unresolved- H173, H304, and D195 -were varied in a heuristic manner until a fully open and continuous water-filled pore was achieved in both protomers.

Modeling of *syn*-cycle states

P_{480}/C_2 state: The fully equilibrated dark-adapted D_{470}/C_1 closed state system was used as a starting point to build the light-adapted closed state of the *syn*-cycle, P_{480}/C_2. To model the P_{480}/C_2 state, the all-*trans* retinal was replaced by the structure of the retinal in the 13-*cis*, 15-*syn* conformation from the TR-SFX crystal structure of C1C2 (PDB: 7E6X) [6]. This was done in both protomers. In accordance with experimental data, residue D195 and the Schiff base were modeled as protonated, E129 was modeled as deprotonated, and all other residues were modeled in their standard states at physiological pH [9,22,23].

13-*cis*, 15-*syn* → 13-*trans*, 15-*syn* retinal isomerization: The retinal in the 13-*cis*, 15-*syn* conformation of the P_{480}/C_2 state is photoactive [22–24], and undergoes isomerization to 13-*trans*, 15-*syn* upon exposure to blue light (λ = 480 nm) to form the light-adapted low-conducting I_{530}/O_2 open state [9,23]. The fully equilibrated P_{480}/C_2 closed state system including the protein dimer, membrane lipids, water, and ions was used to approximate the photoisomerization of the retinal from 13-*cis*, 15-*syn* → 13-*trans*, 15-*syn*. The same procedure was carried out in both protomers. The dihedral angle formed by atoms C12-C13=C14-C15 was rotated in 20º steps from 0º to 180º while the C15=N dihedral angle was held at 0º. Between each rotation step, dihedral restraints were placed on the retinal polyene chain to maintain planarity while the rest of the system was allowed to relax for 1 ns. Once isomerization was complete, the system was relaxed for an additional 9 ns.

Steered molecular dynamics

Preliminary exploration of the permeation pathway was performed by pulling the ion through the channel pore at constant velocity using steered molecular dynamics (SMD). These simulations provided an initial estimate of the primary free energy barriers along the pathway, and the resulting trajectories were used to generate starting states for subsequent multiple walker metadynamics simulations. All SMD simulations were performed in NAMD 2.14 using the built-in SMD module to apply steering forces. Starting with a pre-equilibrated open-state system, Na^+ or Ca^{2+} was placed just outside the pore entrance on the extracellular side of protomer B formed by residues E140, V156, and R159. The system was briefly equilibrated again with the ion held in place. Positional restraints in the z-direction with a force constant of 5.0 kcal/mol·Å^2 were applied to several backbone C_α atoms on the periphery of the protein to prevent drift due to pulling forces, while no restraints were implemented in the x- or y-directions. The ion was then pulled through the water-filled pore formed by transmembrane helices I, II, III, and VII toward the intracellular gate at a constant pulling speed of 0.005 Å/ps and spring constant of 5.0 kcal/mol·Å^2. Results are plotted in S4 Fig.

Computational approach for calculating the PMF

One way to gain insight into the energetics of the transport process and relevant ion-protein interactions is through free energy calculations. Determination of the free energy surface, or the potential of mean force profile (PMF), of ion translocation through an aqueous channel pore can reveal crucial details about its mechanism, where free energy wells can indicate the locations of key ion binding sites in the channel, and peaks can identify the free energy barriers the ion encounters along the pathway.

However, obtaining a meaningful PMF profile for cation conduction in C1C2 requires overcoming two main challenges. The first challenge pertains to the need for practical simulation times with limited compute power. As the rate of cation passage through channelrhodopsins is estimated to be quite slow with a turnover number of just 2.2 x 10^4 ions per second for Na^+ [20], a direct simulation of this process would require prohibitively long simulation times and a large computational resource. Furthermore, most enhanced sampling methods that are typically employed to accelerate PMF calculations for such rare events, such as umbrella sampling [25] or free energy perturbation [26], require a detailed knowledge of the reaction coordinate, or collective variable (CV), and the free energy barriers along it *a priori*.

Considering the open questions regarding the permeation pathway discussed earlier, this requirement presents our second challenge of choosing a suitable CV, since an incorrect choice of reaction coordinate can lead to large errors in the resulting PMF. One technique that tackles both of the aforementioned problems simultaneously is metadynamics [27], specifically the well-tempered and multiple-walker variants of the method [28,29]. In metadynamics, the sampling along one or two slow-moving CVs is accelerated by adding a history-dependent bias potential on top of the underlying potential energy function in the form of repulsive Gaussian hills. Throughout the simulation, hills are periodically deposited at the current location in the CV space and accumulate to produce a flat biased free energy surface that leads to even sampling throughout all regions of the CV.

This process allows an ion trapped in a local free energy well to overcome the lowest adjacent free energy barrier and diffuse into the next local free energy minimum. In this way, the ion is encouraged to progress along the channel into previously unexplored regions while building up the bias potential for each new point in the CV along the way. The unbiased PMF is then estimated directly from the sum of all deposited Gaussian hills as a function of the CV value.

In the well-tempered (WT) variation, the CV is sampled according to the well-tempered distribution which effectively samples the CV at a higher temperature ($T+\Delta T$) than the system simulation temperature (T) [28]. The height of the added Gaussians also decreases as the simulation proceeds at a rate dependent on the set value of ΔT. If the value of ΔT is set according to the height of the typical free energy barrier of the event under study, then the exploration of the CV space is limited to regions where the free energy is not much higher than the typical barrier. Consequently, well-tempered metadynamics simulations can overcome the difficulties presented by an uncertain reaction coordinate because it automatically identifies and samples the lowest free energy path for cations through the channel, thereby doubling as an efficient path sampling method [30,31]. The method also avoids artifacts from overfilling of free energy wells and ensures convergence of the final PMF [32].

Lastly, the multiple-walker variant of metadynamics runs several replicas, or "walkers", of the system in tandem where each walker has a different starting point along the CV [29]. Gaussian hills deposited by each walker are combined at regular intervals to form a single underlying biasing potential that acts on all walkers in the system, and all walkers contribute to the final PMF. Combining the multiple-walker and well-tempered metadynamics variants substantially reduces the wall time required to complete simulations of rare events by accelerating sampling across the entire CV space and makes efficient use of available computational resources.

References

1. VanGordon MR, Gyawali G, Rick SW, Rempe SB. Atomistic Study of Intramolecular Interactions in the Closed-State Channelrhodopsin Chimera, C1C2. Biophys J. 2017 Mar;112(5):943–52.
2. Kato HE, Zhang F, Yizhar O, Ramakrishnan C, Nishizawa T, Hirata K, et al. Crystal structure of the channelrhodopsin light-gated cation channel. Nature. 2012 Jan 22;482(7385):369–74.
3. Becker-Baldus J, Bamann C, Saxena K, Gustmann H, Brown LJ, Brown RCD, et al. Enlightening the photoactive site of channelrhodopsin-2 by DNP-enhanced solid-state NMR spectroscopy. Proc Natl Acad Sci U S A. 2015 Aug 11;112(32):9896–901.
4. Inaguma A, Tsukamoto H, Kato HE, Kimura T, Ishizuka T, Oishi S, et al. Chimeras of Channelrhodopsin-1 and -2 from Chlamydomonas reinhardtii Exhibit Distinctive Light-induced Structural Changes from Channelrhodopsin-2. J Biol Chem. 2015 May 1;290(18):11623–34.
5. Hontani Y, Marazzi M, Stehfest K, Mathes T, van Stokkum IHM, Elstner M, et al. Reaction dynamics of the chimeric channelrhodopsin C1C2. Sci Rep. 2017 Aug 3;7(1):7217.
6. Oda K, Nomura T, Nakane T, Yamashita K, Inoue K, Ito S, et al. Time-resolved serial femtosecond crystallography reveals early structural changes in channelrhodopsin. eLife. 2021 Mar 23;10:e62389.
7. Kuhne J, Eisenhauer K, Ritter E, Hegemann P, Gerwert K, Bartl F. Early Formation of the Ion-Conducting Pore in Channelrhodopsin-2. Angew Chem Int Ed. 2015 Apr 13;54(16):4953–7.
8. Lórenz-Fonfría VA, Bamann C, Resler T, Schlesinger R, Bamberg E, Heberle J. Temporal evolution of helix hydration in a light-gated ion channel correlates with ion conductance. Proc Natl Acad Sci U S A. 2015 Oct 27;112(43):E5796–804.
9. Kuhne J, Vierock J, Tennigkeit SA, Dreier MA, Wietek J, Petersen D, et al. Unifying photocycle model for light adaptation and temporal evolution of cation conductance in channelrhodopsin-2. Proc Natl Acad Sci. 2019 May 7;116(19):9380–9.
10. Zhu S, Brown MF, Feller SE. Retinal Conformation Governs pKa of Protonated Schiff Base in Rhodopsin Activation. J Am Chem Soc. 2013 Jun 26;135(25):9391–8.
11. Lórenz-Fonfría VA, Resler T, Krause N, Nack M, Gossing M, Fischer von Mollard G, et al. Transient protonation changes in channelrhodopsin-2 and their relevance to channel gating. Proc Natl Acad Sci U S A. 2013 Apr 2;110(14):E1273–81.
12. Ritter E, Piwowarski P, Hegemann P, Bartl FJ. Light-dark Adaptation of Channelrhodopsin C128T Mutant. J Biol Chem. 2013 Apr 12;288(15):10451–8.
13. Schneider F, Grimm C, Hegemann P. Biophysics of Channelrhodopsin. Annu Rev Biophys. 2015 Jun 22;44(1):167–86.
14. Ito S, Kato HE, Taniguchi R, Iwata T, Nureki O, Kandori H. Water-Containing Hydrogen-Bonding Network in the Active Center of Channelrhodopsin. J Am Chem Soc. 2014 Mar 5;136(9):3475–82.
15. VanGordon MR, Prignano LA, Dempski RE, Rick SW, Rempe SB. Channelrhodopsin C1C2: Photocycle kinetics and interactions near the central gate. Biophys J. 2021 May;120(9):1835–45.
16. Heberle J. Proton transfer reactions across bacteriorhodopsin and along the membrane. Biochim Biophys Acta BBA - Bioenerg. 2000 May 12;1458(1):135–47.

17. Nagel G, Ollig D, Fuhrmann M, Kateriya S, Musti AM, Bamberg E, et al. Channelrhodopsin-1: A Light-Gated Proton Channel in Green Algae. Science. 2002 Jun 28;296(5577):2395–8.
18. Lin JY, Lin MZ, Steinbach P, Tsien RY. Characterization of Engineered Channelrhodopsin Variants with Improved Properties and Kinetics. Biophys J. 2009 Mar 4;96(5):1803–14.
19. Nagel G, Brauner M, Liewald JF, Adeishvili N, Bamberg E, Gottschalk A. Light Activation of Channelrhodopsin-2 in Excitable Cells of Caenorhabditis elegans Triggers Rapid Behavioral Responses. Curr Biol. 2005 Dec 24;15(24):2279–84.
20. Feldbauer K, Zimmermann D, Pintschovius V, Spitz J, Bamann C, Bamberg E. Channelrhodopsin-2 is a leaky proton pump. Proc Natl Acad Sci U S A. 2009 Jul 28;106(30):12317–22.
21. Berndt A, Lee SY, Wietek J, Ramakrishnan C, Steinberg EE, Rashid AJ, et al. Structural foundations of optogenetics: Determinants of channelrhodopsin ion selectivity. Proc Natl Acad Sci U S A. 2016 Jan 26;113(4):822–9.
22. Saita M, Pranga-Sellnau F, Resler T, Schlesinger R, Heberle J, Lorenz-Fonfria VA. Photoexcitation of the P_4^{480} State Induces a Secondary Photocycle That Potentially Desensitizes Channelrhodopsin-2. J Am Chem Soc. 2018 Aug 8;140(31):9899–903.
23. Becker-Baldus J, Leeder A, Brown LJ, Brown RCD, Bamann C, Glaubitz C. The Desensitized Channelrhodopsin-2 Photointermediate Contains 13-cis,15-syn Retinal Schiff Base. Angew Chem Int Ed. 2021 May 11;16442–7.
24. Bruun S, Stoeppler D, Keidel A, Kuhlmann U, Luck M, Diehl A, et al. Light–Dark Adaptation of Channelrhodopsin Involves Photoconversion between the all-*trans* and 13-*cis* Retinal Isomers. Biochemistry. 2015 Sep 8;54(35):5389–400.
25. Kästner J. Umbrella sampling. WIREs Comput Mol Sci. 2011;1(6):932–42.
26. Zwanzig RW. High-Temperature Equation of State by a Perturbation Method. I. Nonpolar Gases. J Chem Phys. 1954 Aug 1;22(8):1420–6.
27. Laio A, Parrinello M. Escaping free-energy minima. Proc Natl Acad Sci U S A. 2002 Oct 1;99(20):12562–6.
28. Barducci A, Bussi G, Parrinello M. Well-Tempered Metadynamics: A Smoothly Converging and Tunable Free-Energy Method. Phys Rev Lett. 2008 Jan 18;100(2):020603.
29. Raiteri P, Laio A, Gervasio FL, Micheletti C, Parrinello M. Efficient Reconstruction of Complex Free Energy Landscapes by Multiple Walkers Metadynamics. J Phys Chem B. 2006 Mar 1;110(8):3533–9.
30. Zhang Y, Voth GA. Combined Metadynamics and Umbrella Sampling Method for the Calculation of Ion Permeation Free Energy Profiles. J Chem Theory Comput. 2011 Jul 12;7(7):2277–83.
31. Nishihara Y, Hayashi S, Kato S. A search for ligand diffusion pathway in myoglobin using a metadynamics simulation. Chem Phys Lett. 2008 Oct 23;464(4):220–5.
32. Dama JF, Parrinello M, Voth GA. Well-Tempered Metadynamics Converges Asymptotically. Phys Rev Lett. 2014 Jun 18;112(24):240602.
