# Supplementary material for: Metadynamics simulations reveal mechanisms of Na+ and Ca2+ transport in two open states of the channelrhodopsin chimera, C1C2
Source: PLoS One. 2024 Sep 6;19(9):e0309553. doi: 10.1371/journal.pone.0309553 (PMC11379304; doi:10.1371/journal.pone.0309553)
Supplement: S2 Text — Presents modeling results for photocycle states D470/C1, P500, P390, and P480/C2 for the dimeric wild-type C1C2 and N297D mutant channels. (PDF) [file pone.0309553.s018.pdf]

Supporting information: Results and discussion

Structural analysis of photocycle state models

Here, we present modeling results of dimeric wild-type C1C2 and N297D mutant channels for all non-conducting and intermediate photocycle states of the *anti*- and *syn*-cycles. For a discussion of the modeling results for the conductive P_{520}/O_1 and I_{530}/O_2 open states of the dimeric wild-type C1C2 and N297D mutant channels, please see the main text. Configurations of all simulated models are listed in S3-S6 Tables. Simulations of all protein models converged within the first 60 ns of unrestrained production run (S2 Fig).

Dark-adapted closed state D_{470}/C_1. Residues in the disordered first intracellular loop and C- and N-termini that were missing from the crystal structure of the closed state C1C2 (PDBID: 3UG9) were modeled using the I-TASSER server [1–3]. The server gave the final model a confidence score of 0.00 (range [-5, 2]), where a score > -1.5 is indicative of an accurate structure prediction [2]. The completed dimeric protein was then inserted into a DOPC lipid bilayer with water and ions and equilibrated as described in the Methods. For the wild-type channel, the helix backbone RMSD of the fully equilibrated protein plateaued at 1.31 ± 0.01 Å against the crystal structure, indicating they are in good agreement (S2 Fig). This is an improvement over our previous closed state model of the C1C2 monomer, which had a backbone RMSD of 1.7 Å against the crystal structure [4]. This result suggests that dimerization plays a modest role in stabilizing the protein's tertiary structure in the D_{470}/C_1 closed state.

In the present model, the distance between pore-forming helices II and VII at the intracellular side was 5.79 ± 0.02 Å in protomer A and 6.36 ± 0.02 Å in protomer B (S3 Table). These values are nearly identical to the 5.78-Å helix separation measured from the closed state X-ray crystal structures of C1C2 (PDBID: 3UG9 and 7C86) [5,6]. During the simulation, additional water molecules entered the channel from the bulk solvent on the extracellular side (S3 Fig). This brought the total number of waters in the pore up from 10 in protomer A and 11 in protomer B identified in the crystal structure to an average of 48 ± 0.2 in protomer A and 47 ± 0.2 in protomer B (S3 Table).

Results were similar for the D_{470}/C_1 state of the N297D mutant which had an average backbone helix RMSD of 1.47 ± 0.01 Å against the wild-type crystal structure and average pore water count of 47 ± 0.2 in protomer A and 40 ± 0.2 in protomer B (S4 Table). The separation between helices II and VII at the intracellular side was slightly larger compared to the wild-type channel and measured 7.06 ± 0.02 Å in protomer A and 6.47 ± 0.03 in protomer B.

In the central gating region of the wild-type channel, the structure of the hydrogen bonding network in protomer A closely resembled that of the crystal structure, while some variations were seen in protomer B (S4 Fig). In protomer A, the protonated Schiff base formed an H-bond with counterion D292. The other counterion to the $RSBH^+$, E162, formed a salt bridge with K132. The carbonyl oxygen of the N297 sidechain formed a strong H-bond with protonated E129, while the amide nitrogen of N297 donated a weak H-bond with the oxygen on S102. The interactions between S102, N297, and E129 maintained a close connection between helices I, II, and VII that occluded the pore and kept the channel closed.

A schematic representation of the interaction network in protomer B is provided in S4 Fig. The central gating structure in protomer B was similar to protomer A, except K132 formed a salt bridge with E136 in the extracellular vestibule instead of with E162. This left E162 free to accept

an H-bond from T166, which placed its sidechain closer to the $RSBH^+$. As a result, the retinal Schiff base in protomer B frequently alternated H-bonding between E162 and D292 throughout the trajectory. Ground state heterogeneity between the two protomers in this manner was suggested previously by QM/MM calculations of the C1C2 active site, [7] and may help explain the apparent conflicting reports in the literature that identify either E162 or D292 as the primary proton acceptor based on the ground state structure [8–10].

In the N297D mutant, sidechains in the central gating region were arranged in a similar manner to the wild-type channel but had a more interconnected hydrogen bonding network (S4 Fig). In both protomers, additional H-bonds were observed between sidechains E129 and K132. The sidechain of K132 formed additional salt bridges with E162 and occasionally D292, while the other carboxylate oxygen of E162 hydrogen bonded with T166 one helix turn away. In protomer A, the $RSBH^+$ formed a hydrogen bond with only D292, while in protomer B the $RSBH^+$ frequently alternated between D292 and E162 throughout the trajectory. Also of note was the presence of double H-bonds between E136 and R159 (located in the extracellular vestibule) in both protomers that were absent in the wild-type channel.

The last region of interest discussed here is the so-called "DC-gate" formed by residues C167 and D195 (Fig 2E). Despite their location outside of the permeation pathway in the retinal binding pocket, these two residues are critically important for transitions between photocycle states, unitary channel conductance, and cation selectivity in most ChRs [11–14]. Early FTIR studies showed evidence that C167 and D195 interact via H-bond either directly or through a water molecule in the ground state (D_{470}/C_1), [15] but no such interaction was observed in the C1C2 crystal structure of this state [6].

In our ground state model for the wild-type channel, although no water molecules entered the vicinity of the DC-gate during the 100-ns trajectory, the two residues were close enough to intermittently form a weak electrostatic interaction in one protomer. In protomer B, the D195-O-H···S-C167 bond had an average bond distance of 2.56 ± 0.02 Å and bond angle of 145 ± 1° and meets the criteria for a weak hydrogen bond [16]. In protomer A, however, their sidechains were much further apart (> 4 Å) and could not form a hydrogen bond.

In the N297D mutant, the two residues formed hydrogen bonds in both protomers. In protomer A, the C167 thiol group donated a bifurcated H-bond to the two oxygens of the D195 sidechain. The interaction in protomer B resembled that of the wild-type channel where the thiol group was the H-bond acceptor and had a D195-O-H···S-C167 bond distance of 2.54 ± 0.02 Å and a bond angle of 147 ± 1°. This behavior contrasts with our previous closed state model of the C1C2 monomer, where C167 and D195 were too far apart (> 4 Å) to interact in both the wild-type and N297D channels [4].

***Anti*-cycle photointermediate P_{500}.** Starting from the D_{470}/C_1 state for both proteins, retinal isomerization from all-*trans*, 15-*anti* à 13-*cis*, 15-*anti* formed the first photointermediate state of the *anti*-cycle, P_{500}. This transition altered the orientation of the Schiff base proton to point toward the intracellular gate instead of the extracellular vestibule, and its H-bond with D292 was disrupted. The equilibrated structures of the P_{500} state had a protein backbone RMSD of 0.66 ± 0.01 Å for the wild-type channel and 0.68 ± 0.01 Å for the N297D mutant against the unequilibrated structures, indicating only minimal movement of the protein backbone due to retinal isomerization for both proteins. This movement was limited to a small, local distortion of helix 7 in the immediate vicinity of the retinal.

No significant changes to internal water distribution or hydrogen bonding among sidechains were observed, either, aside from the loss of the $RSBH^+ \cdots D292$ salt bridge due to $RSBH^+$ proton reorientation. In fact, D292 still remained in close contact with the retinal polyene chain (< 3.5 Å) after isomerization in both protomers of both proteins, while E162 did not. Since deprotonation of the $RSBH^+$ occurs at the end of the P_{500} state lifetime, this result provides further support for our assignment of D292 as the primary proton acceptor from our previous monomeric model of a C1C2 open state [4].

Previous time-resolved FTIR and UV/Vis flash photolysis studies showed that ultrafast isomerization of retinal and formation of the P_{500} state occur within 450 fs of photon absorption in ChR2 and C1C2 [7,8,17]. This state has a relatively short lifetime of just ~700 ns, [8] during which only small shifts in the protein backbone were seen in the TR-SFX study, [5] and no helix hydration was apparent in vibrational spectra until after the formation of the P_{390} state at a much later timepoint [17]. This is consistent with electrophysiology measurements that suggest the P_{500} state is nonconductive [17,18]. Although the present study does not aim to replicate the time course of such events, our results from simulations of the P_{500} state models are in excellent agreement with these experimental findings.

***Anti*-cycle "pre-open" intermediate P_{390}.** The P_{390} state structures for dimeric wild-type C1C2 and N297D mutant channels were constructed from their respective equilibrated P_{500} state models. This state corresponds to an early open state that conducts protons but not larger cations in electrophysiology studies [17]. Therefore, this state is often called the "pre-open" intermediate in the literature.

Slight shifts in the protein backbone and rearrangement of key hydrogen bonding interactions were observed upon equilibration of the P_{390} state of the wild-type C1C2 channel. Results were similar for both protomers. Moving the proton from the retinal Schiff base to D292 triggered a downward movement of the retinal polyene chain, increasing the distance between the two residues. This motion caused the local distortion of the helix VII backbone near the retinal that was initiated in the P_{500} state to become more pronounced. Protonated D292 initially became more mobile as it moved up and away from the RSB, but ultimately settled in a new position stabilized by two hydrogen bonds: one with the NH-group on W163, and another indirectly with E162 through a bridging water molecule (S4 Fig). In the central gate, the E129···N297 H-bond was disrupted and E129 moved to form a new H-bond with E162, while the S102···N297 interaction was preserved.

Throughout the simulation, the sidechain of C167 of the DC-gate gradually turned further toward D195 to create a more stable interaction between them. Small shifts in the protein backbone were also observed in helices I and II that increased the separation between helices II and VII, predominantly on the intracellular side. This resulted in a marked weakening and increased instability of the hydrogen bonding network among residues of the inner gate. Specifically, the sidechain of E121 separated from its hydrogen bonding partner R307, while the E122···R307 and E122···H304 interactions stayed intact. This resulted in partial opening of the intracellular gate that allowed some water molecules to enter the channel on the cytosolic side, although a continuous water-filled pore did not form (S3 Fig).

Overall, there were about 5-8 fewer water molecules inside the pore in both protomers at the end of the P_{390} state simulation compared to the D_{470}/C_1 closed state model (S3 Table). This was due to a loss of pore water in the extracellular vestibule, as made evident by inspection of the water distribution in the channel shown in S3 Fig. As shown in the figure, the central region of the

channel remained dry at the end of the 100-ns simulation even though the pathway was no longer occluded by the E129···N297 interaction. Time-resolved FTIR studies tracking helix hydration in ChR2 and C1C2 demonstrated that water influx starts from the intracellular side upon formation of the P_{390} state and increases until a continuous water-filled pore is formed with a time constant of about $\tau \sim 200$ μs [17,18]. Therefore, results from our comparatively short 100-ns simulation are consistent with structure and hydration changes expected during the very early stages of channel opening in the P_{390} state.

In the P_{390} state of the N297D mutant channel, shifts in the protein backbone and rearrangement of sidechains closely resembled those of the wild-type protein, but with some variations (S4 Fig). Protonated D292 initially became more mobile as it separated from the retinal as in the wild-type protein and formed a new H-bond indirectly with E162 through a water molecule in one protomer, but directly to E162 in the other protomer. Rearrangement of hydrogen bonds among central gating residues were also like the wild-type, with the exception that the S102···D297 H-bond was not preserved in the N297D channel. Interestingly, the sidechain of R159 in the conserved cluster (see Fig 2D) repositioned to form a new H-bond with E136 in the extracellular vestibule, whereas these residues remained in their closed state (D_{470}/C_1) positions in the wild-type channel.

At the intracellular gate, weakening of electrostatic interactions among gating residues led to the influx of an additional 6-10 water molecules from the cytosolic side in both protomers compared to the D_{470}/C_1 closed state. This brought the total number of waters in the pore up to 57 ± 0.3 in protomer A and 46 ± 0.3 in protomer B (S4 Table). Unlike the wild-type protein, the additional water permeated the central region of the channel to form a continuous water-filled pore. However, the pore was only wide enough to accommodate a chain of water molecules in a single file that was frequently interrupted due to the dynamics of the partially closed inner gate.

Formation of a very narrow pore in this state is consistent with electrophysiology measurements that show that ion flow begins once the pore helices are fully hydrated during the latter part of the P_{390} state lifetime, but only protons are conducted in this state [8,17,19]. The onset of photocurrent carried by larger cations such as Na^+ and Ca^{2+} is correlated with the transition from the P_{390} state to the high-conducting P_{520}/O_1 open state and occurs within ~2-3 ms of photon absorption [17].

***Syn*-cycle light-adapted closed state P_{480}/C_2.** In the wild-type C1C2 channel, the transition from the first closed state, D_{470}/C_1, to the second closed state, P_{480}/C_2, resulted in some rearrangement of sidechain interactions among central gating residues (S5 Fig) and small shifts in the protein backbone. Unlike in the *anti*-cycle, the $RSBH^+$ proton of retinal in the 13-*cis*, 15-*syn* conformation stays oriented toward the extracellular side of the channel. Thus, the $RSBH^+$···O-D292 interaction was kept intact in both protomers during the P_{480}/C_2 closed state simulation.

Deprotonation of E129 disrupted its interaction with N297. Instead, E129 formed two new H-bonds with S102 and K132 (S5 Fig). This partially opened up the CG and allowed an additional ~6-8 water molecules to enter the channel from the extracellular side and occupy the central region of the channel down to the inner gate (S3 Fig). The sidechain of K132 also formed a new H-bond with the free oxygen on D292 such that E129, E162, and D292 all formed strong interactions with K132. Sidechain interaction networks were the same in both protomers except for the position of the R159 sidechain in the extracellular vestibule. In protomer A, R159 moved down to form salt bridges with E136 and E162. While in protomer B, R159 remained hydrogen bonded to T285 as in the D_{470}/C_1 closed state.

The retinal polyene chain adopted a slight twist toward helix III that moved C167 closer to D195 to form a stronger H-bond between them with D195 as the H-bond donor. This induced a lateral movement of the middle part of helix III that translated to a slight outward shift of the intracellular end of helix II by ~0.7-2.3 Å (S5 Table). The increased distance between helices II and VII led to weakening of H-bond interactions among sidechains of the ICG, but the ICG remained closed.

Results for the N297D mutant channel in the P_{480}/C_2 closed state were very similar to wild-type C1C2. Double-isomerization of the retinal to the 13-*cis*, 15-*syn* conformer led to a slight twist in the retinal polyene chain toward helix III that ultimately translated to an outward shift of the intracellular end of helix II by ~1.4-2 Å (S6 Table). In the central region of the channel, the $RSBH^+$ proton remained oriented toward the extracellular side and H-bonded with D292 only (S5 Fig).

Rearrangement of sidechain interactions within the central gate allowed an additional ~9-14 water molecules from the extracellular vestibule to fill the central region of the channel. The outward movement of helix II weakened the H-bonding network among sidechains of the inner gate, and E121 broke away from H173 and R307 to form a new salt bridge with K186 of the opposing protomer. The ICG stayed closed, however, due the remaining H304···E122···R307 sidechain interactions that prevented water influx from the intracellular side.

References

1. Zhang Y. I-TASSER server for protein 3D structure prediction. BMC Bioinformatics. 2008 Jan 23;9(1):40.
2. Roy A, Kucukural A, Zhang Y. I-TASSER: a unified platform for automated protein structure and function prediction. Nat Protoc. 2010 Apr;5(4):725–38.
3. Yang J, Yan R, Roy A, Xu D, Poisson J, Zhang Y. The I-TASSER Suite: protein structure and function prediction. Nat Methods. 2015 Jan;12(1):7–8.
4. VanGordon MR, Prignano LA, Dempski RE, Rick SW, Rempe SB. Channelrhodopsin C1C2: Photocycle kinetics and interactions near the central gate. Biophys J. 2021 May;120(9):1835–45.
5. Oda K, Nomura T, Nakane T, Yamashita K, Inoue K, Ito S, et al. Time-resolved serial femtosecond crystallography reveals early structural changes in channelrhodopsin. eLife. 2021 Mar 23;10:e62389.
6. Kato HE, Zhang F, Yizhar O, Ramakrishnan C, Nishizawa T, Hirata K, et al. Crystal structure of the channelrhodopsin light-gated cation channel. Nature. 2012 Jan 22;482(7385):369–74.
7. Hontani Y, Marazzi M, Stehfest K, Mathes T, van Stokkum IHM, Elstner M, et al. Reaction dynamics of the chimeric channelrhodopsin C1C2. Sci Rep. 2017 Aug 3;7(1):7217.
8. Kuhne J, Eisenhauer K, Ritter E, Hegemann P, Gerwert K, Bartl F. Early Formation of the Ion-Conducting Pore in Channelrhodopsin-2. Angew Chem Int Ed. 2015 Apr 13;54(16):4953–7.
9. Lórenz-Fonfría VA, Resler T, Krause N, Nack M, Gossing M, Fischer von Mollard G, et al. Transient protonation changes in channelrhodopsin-2 and their relevance to channel gating. Proc Natl Acad Sci U S A. 2013 Apr 2;110(14):E1273–81.
10. Sineshchekov OA, Govorunova EG, Wang J, Li H, Spudich JL. Intramolecular Proton Transfer in Channelrhodopsins. Biophys J. 2013 Feb 19;104(4):807–17.

11. Berndt A, Yizhar O, Gunaydin LA, Hegemann P, Deisseroth K. Bi-stable neural state switches. Nat Neurosci. 2009 Feb;12(2):229–34.
12. Bamann C, Gueta R, Kleinlogel S, Nagel G, Bamberg E. Structural Guidance of the Photocycle of Channelrhodopsin-2 by an Interhelical Hydrogen Bond. Biochemistry. 2010 Jan 19;49(2):267–78.
13. Ritter E, Piwowarski P, Hegemann P, Bartl FJ. Light-dark Adaptation of Channelrhodopsin C128T Mutant. J Biol Chem. 2013 Apr 12;288(15):10451–8.
14. Hososhima S, Sakai S, Ishizuka T, Yawo H. Kinetic Evaluation of Photosensitivity in Bi-Stable Variants of Chimeric Channelrhodopsins. PLoS ONE. 2015 Mar 19;10(3):e0119558.
15. Nack M, Radu I, Gossing M, Bamann C, Bamberg E, Mollard GF von, et al. The DC gate in Channelrhodopsin-2: crucial hydrogen bonding interaction between C128 and D156. Photochem Photobiol Sci. 2010 Feb 3;9(2):194–8.
16. Grabowski SJ. Chapter 1 Hydrogen Bond – Definitions, Criteria of Existence and Various Types. In: Understanding Hydrogen Bonds: Theoretical and Experimental Views [Internet]. Cambridge: The Royal Society of Chemistry; 2021. p. 1–40. (Theoretical and Computational Chemistry Series). Available from: http://dx.doi.org/10.1039/9781839160400-00001
17. Lórenz-Fonfría VA, Bamann C, Resler T, Schlesinger R, Bamberg E, Heberle J. Temporal evolution of helix hydration in a light-gated ion channel correlates with ion conductance. Proc Natl Acad Sci U S A. 2015 Oct 27;112(43):E5796–804.
18. Krause BS, Kaufmann JCD, Kuhne J, Vierock J, Huber T, Sakmar TP, et al. Tracking Pore Hydration in Channelrhodopsin by Site-Directed Infrared-Active Azido Probes. Biochemistry. 2019 Mar 5;58(9):1275–86.
19. Kuhne J, Vierock J, Tennigkeit SA, Dreier MA, Wietek J, Petersen D, et al. Unifying photocycle model for light adaptation and temporal evolution of cation conductance in channelrhodopsin-2. Proc Natl Acad Sci. 2019 May 7;116(19):9380–9.
